# A Comparative Study on the Iron and Copper Binding Properties of 8‑Hydroxyquinoline-Derived Mannich Bases Targeting Multidrug-Resistance Cancer Cells

**DOI:** 10.1021/acsomega.5c06872

**Published:** 2026-01-06

**Authors:** Hilda Kovács, Bálint Hajdu, Nóra V. May, Norbert Lihi, István Szatmári, Gergely Szakács, Éva A. Enyedy

**Affiliations:** † Department of Molecular and Analytical Chemistry, Interdisciplinary Excellence Centre, University of Szeged, Dóm tér 7-8, H-6720 Szeged, Hungary; ‡ Centre for Structural Science, 280964 HUN-REN Research Centre for Natural Sciences, Magyar tudósok körútja 2, H-1117 Budapest, Hungary; § HUN-REN-DE Mechanisms of Complex Homogeneous and Heterogeneous Chemical Reactions Research Group, Department of Inorganic and Analytical Chemistry, 37599 University of Debrecen, Egyetem tér 1, H-4032 Debrecen, Hungary; ∥ Institute of Pharmaceutical Chemistry and HUN REN-SZTE Stereochemistry Research Group, University of Szeged, Eötvös u. 6, H-6720 Szeged, Hungary; ⊥ Center for Cancer Research, 27271 Medical University of Vienna, Borschkegasse 8a, A-1090 Vienna, Austria; # Institute of Molecular Life Sciences, HUN-REN Research Centre for Natural Sciences, Magyar tudósok körútja 2, H-1117 Budapest, Hungary; ¶ National Laboratory for Drug Research and Development, Magyar tudósok körútja 2, H-1117 Budapest, Hungary

## Abstract

The efficacy of 8-hydroxyquinoline (HQ) Mannich bases against multidrug-resistant (MDR) cancer cells is thought to be linked to the complexation with essential metal ions such as iron and copper. Here, the complex formation equilibria of five MDR-selective HQs with Fe­(II), Fe­(III), and Cu­(II) were studied by UV–visible spectrophotometry, complemented by electron paramagnetic resonance, circular dichroism, and electrospray ionization mass spectrometry techniques. Cyclic voltammetry and spectroelectrochemistry were used to map the redox characteristics of the complexes, and their direct reactivity with glutathione was also monitored. Single-crystal X-ray diffraction was applied to determine the structures of one of the selected ligands (HQCl-l-Pro) and its bis-ligand Cu­(II) complex. The ligands are coordinated to Fe­(II) and Fe­(III) via the (N,O^–^) donor set in all cases, leading to the formation ofmono-, bis-, and tris-ligand complexes. For Cu­(II) complexes of amino acid conjugates, several species have been identified. In addition to mono- and bis-ligand complexes, dimeric species are also formed, supported by density functional theory calculations. At pH 7.4, the carboxylate-containing ligands bind metal ions in the stability order of: Fe­(II) < Fe­(III) < Cu­(II). Ligands without carboxylate side chains (HQCl-pyr and HQCl-pip) and with increased MDR-selectivity showed a higher preference for Fe­(II) than for Fe­(III), also reflected in the more positive redox potentials.

## Graphical abstract

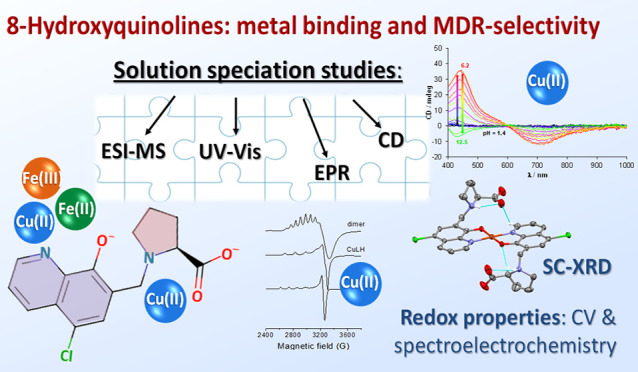

## Introduction

Chemotherapy remains a common treatment for various types of cancer, despite its serious side effects.^1^ However, multidrug resistance (MDR) of cancer cells poses a major challenge to effective treatment, frequently resulting in disease relapse and poor patient prognosis.^2^ The development of multidrug resistance is strongly linked to the upregulated expression of ATP-binding cassette transporters, especially P-glycoprotein (P-gp/ABCB1), which can efflux a broad range of anticancer drugs from cancer cells.^2,3^ Attempts to overcome clinical multidrug resistance using transporter inhibitors have largely failed, necessitating the continued development of alternative therapeutic strategies. An innovative approach to overcoming MDR is to exploit the paradoxical hypersensitivity of multidrug-resistant cells. MDR-selective compounds leverage the collateral sensitivity of these cells, a phenomenon in which MDR cells show increased susceptibility to certain drugs by targeting vulnerabilities associated with P-gp function. Intriguingly, MDR-selective compounds often contain a metal-chelating moiety.^4–6^ Several thiosemicarbazones, as well as 1,10-phenanthroline and 8-hydroxyquinoline (HQ) derivatives, have been identified with MDR-selective activity, all containing metal-chelating groups.^4,5,7^

It is also known that the effect of certain anticancer drugs is linked to their interaction with essential metal ions.^8,9^ In normal tissues, the intracellular concentrations of labile iron and copper pools are low as these metals predominantly exist in complexed forms. Evidence suggests that the increased uptake and retention of iron in cancer cells is metabolically driven, with iron levels often elevated compared to their noncancerous counterparts.^10^ The mechanism of action of at least a subset of MDR-selective compounds was reported to involve complexation with essential metal ions such as copper or iron.^6,11–13^ According to this model, iron depletion is one proposed mechanism, which is more pronounced in MDR cells due to P-gp-mediated efflux of intracellularly formed iron complexes.^11^ On the other hand, chelators that form copper complexes often exert their effects by triggering oxidative stress, as their complexes can generate reactive oxygen species (ROS);^14^ however, other mechanisms are also reported for anticancer copper complexes, including DNA binding, enzyme inhibition, disruption of mitochondrial membrane potential, and interference with protein–protein interactions.^15,16^

A number of HQ-based compounds display a range of biological properties, such as antibacterial, antifungal, and anticancer effects,^17–19^ and these activities are closely connected to their capacity to chelate metal ions. In particular, the planar heterocyclic scaffold with (N,O^–^) donor atoms demonstrates a high affinity for different transition metal ions.^19^ This property also enables HQ derivatives to act as ionophores, facilitating the transfer of metal ions across biological membranes.^20^ For certain HQs, their anticancer activity is modulated by complexation with nonessential metal ions such as gallium­(III), ruthenium­(II), platinum­(II), palladium­(II), or organometallic Rh­(III)­(η^5^-C_5_Me_5_) and Ru­(II)­(η^6^-*p*-cymene).^21–26^

Highly potent anticancer agents have been reported among HQ Mannich bases.^27,28^ In the case of these derivatives, incorporation of a CH_2_–N subunit and a halogen substituent at positions 7 and 5 of the HQ backbone, respectively, is thought to play an important role in their MDR-selective toxicity.^12,13^ However, other substituents on the ligand scaffold can also influence the physicochemical properties and selectivity profile. The acid–base properties and metal-chelating ability of these compounds have previously been identified as key factors contributing to MDR-selective anticancer activity,^12,13^ and we confirmed that there is a correlation between MDR-selectivity and the proton dissociation constants (p*K*_a_) of the compounds.^13^

In this work, we selected five MDR-selective Mannich base derivatives^29,30^ possessing different types of heterocycles at position 7: 5-chloro-7-(pyrrolidin-1-ylmethyl)­8-hydroxyquinoline (HQCl-pyr), 5-chloro-7-(piperidin-1-ylmethyl)­8-hydroxyquinoline (HQCl-pip), (S)-5-chloro-7-((proline-1-yl)­methyl)­8-hydroxyquinoline (HQCl-l-Pro), (R)-5-chloro-7-((proline-1-yl)­methyl)­8-hydroxyquinoline (HQCl-d-Pro), and (R)-5-chloro-7-((homoproline-1-yl)­methyl)­8-hydroxyquinoline (HQCl-d-hPro) (Chart 1). To better understand the structure–property-bioactivity relationship and the mechanism of action of these types of HQ compounds, we performed solution equilibrium studies and investigated their interactions with essential metal ions (copper­(II), iron­(II), and iron­(III)). Although iron­(III) and copper­(II) are not the dominant intracellular oxidation states, they are relevant from both thermodynamic and mechanistic perspectives. The redox state of metal ions in biological systems is dynamic, so both iron­(II)/iron­(III) and copper­(I)/copper­(II) pairs can interconvert readily under physiological conditions, depending on the actual redox potential and ligand environment.

**Chart 1:**
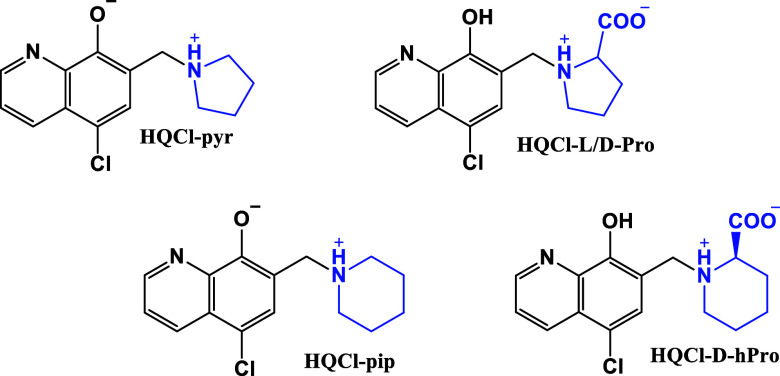
Chemical structure of studied ligands: HQCl-pyr, HQCl-pip, HQCl-l-Pro, HQCl-d-Pro, and HQCl-d-hPro in their neutral form (HL for HQCl-pyr, HQCl-pip, and H_2_L for the amino acid conjugates)

Therefore, the redox behavior of the resulting iron and copper complexes was also investigated, and the interaction of the Cu­(II) complexes with glutathione, a physiologically relevant reductant, was directly followed as well. The properties of the forming iron and copper complexes are compared with those of non-MDR-selective HQs to identify the differences in their solution equilibrium properties.

## Results and Discussion

### Proton Dissociation Processes of the Studied Mannich Derivatives of 8-Hydroxyquinoline

The p*K*_a_ value is a key physicochemical parameter that can influence the pharmacokinetic behavior of a compound, as it determines the actual protonation state and charge of a molecule at a given pH. The p*K*_a_ values of the selected HQ derivatives (Chart 1) were already determined in water in our previous works.^31,32^ Herein, 30% (*v/v*) DMSO/H_2_O was used for the measurements due to the rather low solubility of the forming metal complexes. To elucidate the proton dissociation in this medium, pH-potentiometric and UV–visible (UV–vis) titrations were carried out. UV–vis spectra measured for HQCl-d-hPro are shown in Figure 1a, and the proton dissociation constants obtained by the various methods are summarized in Table 1. The completely protonated forms of HQCl-pyr and HQCl-pip (Chart 1) have three, while HQCl-L/d-Pro and HQCl-d-hPro (Chart 1) have four dissociable protons; however, only three deprotonation processes could be followed (resulting in three p*K*_a_ values with acceptable standard deviations) in the studied pH range (1.0–12.2), namely, those of the quinolinium nitrogen, the OH and the ammonium moiety of the aliphatic ring. The lowest p*K*_a_ (estimated to be <2) of HQCl-L/d-Pro and HQCl-d-hPro corresponds to the deprotonation of the carboxylic group, which could not be determined, as the pH cannot be measured reliably at pH <2. For all five compounds, the deprotonation of the quinolinium nitrogens (N_q_H^+^) occurs in the strongly acidic pH range in this solvent mixture, completing at around pH 3; however, their p*K*_a_ values could be determined only with high uncertainty. Based on the p*K*_a_ values, at pH 7.4, the derivatives containing a carboxylic group show a zwitterionic structure in their H_2_L (N_quinoline_, OH, COO^–^, NH^+^) and HL^–^ (N_quinoline_, O^–^, COO^–^, NH^+^) forms (Figure 1b), and due to this fact, they have better aqueous solubility than HQCl-pyr and HQCl-pip, which are present in their H_2_L^+^ (N_quinoline_, OH, NH^+^) and HL (N_quinoline_, O^–^, NH^+^) forms.

**Figure 1:**
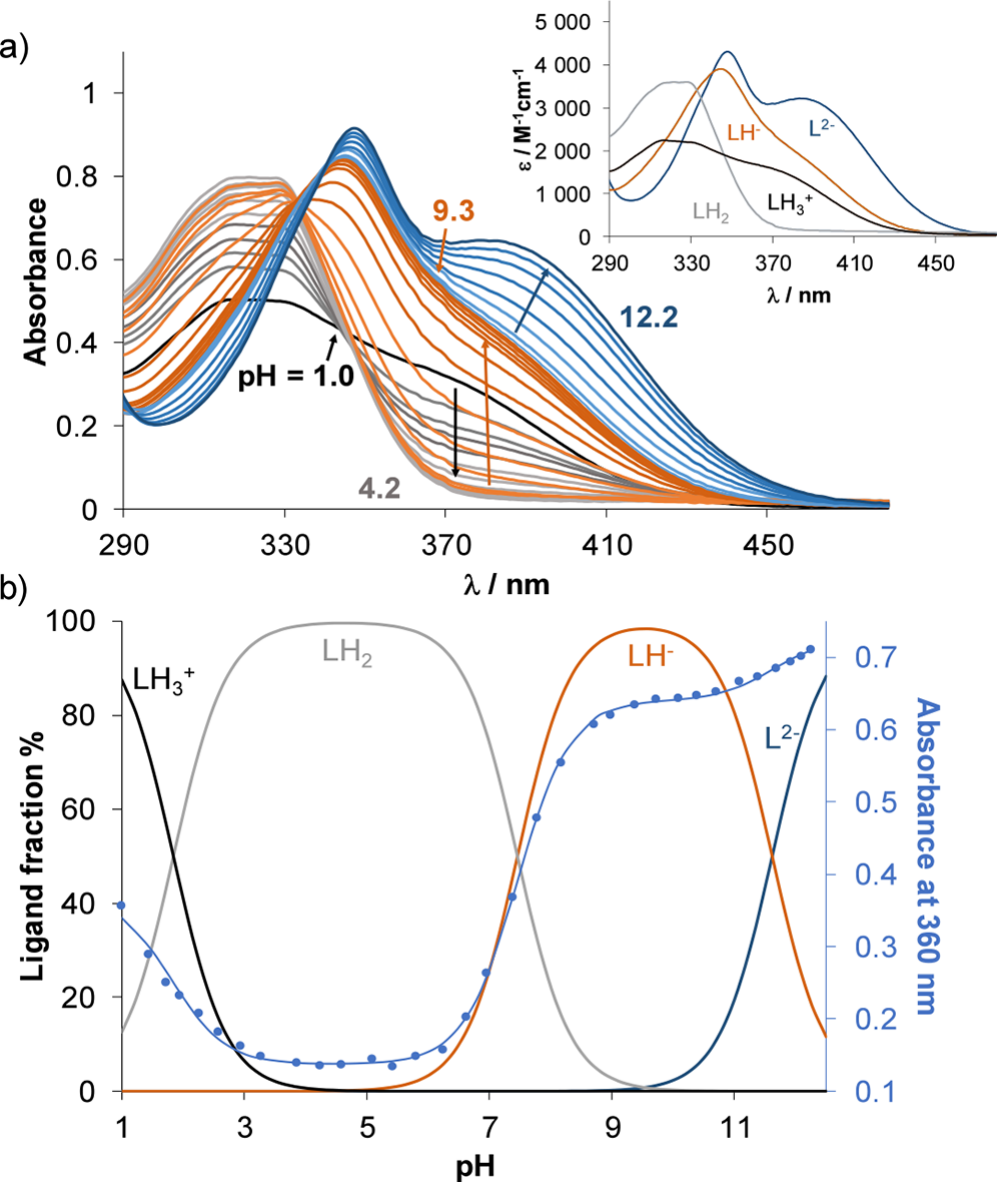
(a) UV–vis absorption spectra measured for HQCl-d-hPro over the pH range 1.0–12.2, and the inset displays the calculated molar absorbance spectra of the ligand in the various protonation states. (b) Concentration distribution curves generated using the p*K*_a_ values determined by UV–vis titrations, and absorbance values at 360 nm (●, cyan) across the pH range shown together with the fitted curve (solid line) {*c*_HQCl‑D‑hPro_ = 108 μM; *l* = 2 cm; *I* = 0.1 M (KCl); 30% (*v/v*) DMSO/H_2_O; *T* = 25.0 °C}.

**Table 1:** Proton Dissociation Constants of 8-Hydroxyquinoline Ligands (Chart 1) Obtained by pH-Potentiometric (pH-Pot.) and UV–Vis Spectroscopic Titrations^a^

	method	p*K*_a_ (N_q_H^+^)	p*K*_a_ (OH)	p*K*_a_ (NH^+^)
HQCl-l-Pro^b^	pH-pot	–	7.61 ± 0.05	11.80 ± 0.03
	UV–vis	1.26 ± 0.05	7.68 ± 0.03	11.89 ± 0.03
HQCl-d-Pro^c^	pH-pot	–	7.60 ± 0.06	11.71 ± 0.08
	UV–vis	1.69 ± 0.05	7.67 ± 0.03	11.82 ± 0.03
HQCl-d-hPro^d^	pH-pot	–	7.42 ± 0.04	11.65 ± 0.03
	UV–vis	1.84 ± 0.05	7.46 ± 0.03	11.62 ± 0.03
HQCl-pyr	pH-pot	–	–^e^	–^e^
	UV–vis	0.9 ± 0.1	7.42 ± 0.03	11.14 ± 0.03
HQCl-pip	pH-pot	–	–^e^	–^e^
	UV–vis	1.0 ± 0.1	7.27 ± 0.03	11.06 ± 0.03

a {30% (*v/v*) DMSO/H_2_O; *I* = 0.1 M (KCl); *T* = 25 °C} p*K*_a_ (N_q_H^+^), p*K*_a_ (OH), and p*K*_a_ (NH^+^) denote the negative decadic logarithm of the proton dissociation constant of the quinolinium nitrogen, the phenolic OH, and the ammonium moiety, respectively.

b p*K*_a_ (N_quinolinium_H^+^) = 2.36; p*K*_a_ (OH) = 7.76; p*K*_a_ (NH^+^) > 11.5 determined by pH-potentiometry in water.^31^

c p*K*_a_ (N_q_H^+^) < 2; p*K*_a_ (OH) = 7.71; p*K*_a_ (NH^+^) > 11 determined by pH-potentiometry in water.^32^

d p*K*_a_ (N_q_H^+^) = 2.42; p*K*_a_ (OH) = 7.52; p*K*_a_ (NH^+^) > 11 determined by pH-potentiometry in water.^32^

e Could not be determined by this method due to limited solubility of the compounds.

We conclude that the p*K*_a_ values obtained in H_2_O or 30% (*v/v*) DMSO/H_2_O solvent mixtures are similar, with only a slight decrease observed in the latter. p*K*_a_ (N_q_H^+^) shows a minor difference in the different solvents, as we described in our previous work, this phenomenon can be explained by the Born electrostatic solvent model.^33^ The p*K*_a_ values of cationic acids (N_q_H^+^) are reduced as the fraction of DMSO is increased owing to the isoelectronic and charge-neutralizing character of their deprotonation. Regarding this model, the p*K*_a_ of OH that gives anionic base is expected to be increased; however, this did not happen in our case. This unusual behavior may be due to the intramolecular hydrogen bond between the deprotonated hydroxyl group and the protonated pyrrolidinium or piperidinium nitrogen.^32^ In our earlier work, we observed a correlation between toxicity in MDR cells and the p*K*_a_ values of the OH group, based on experimentally determined values of 19 compounds, extended with predicted p*K*_a_ values for nearly 100 additional compounds.^12^ The determined p*K*_a_ (OH) values of the five compounds investigated here are significantly lower than that of the non MDR-selective HQ (p*K*_a_ (OH) = 10.15 (30% (*v/v*) DMSO/H_2_O)),^33^ in accordance with their high MDR-selective toxicity. The IC_50_ values in parental and MDR human cancer cell lines with selectivity ratios are collected in Tables S1 and S2.^29,30^

### Complex Formation Equilibria of HQ-Based Mannich Bases with Fe­(II) and Fe­(III)

Based on the previous results, the presence of a metal-chelating group in the HQ ligands is necessary, but not sufficient for MDR-selective toxicity.^6,13^ In addition to acid–base properties, the stability of the forming iron complexes in solution and their redox potential are thought to be key in determining toxicity.^12,13^ To date, only limited stability data are available for the iron complexes of HQ-based Mannich bases,^34,35^ particularly for Fe­(II) complexes. In order to characterize the binding strength of the five HQ derivatives toward Fe­(II) and Fe­(III), complexation equilibria were studied by UV–vis spectrophotometry in 30% (*v/v*) DMSO/H_2_O. For Fe­(II) complexes, the measurements were carried out in a laboratory glovebox to ensure the inert atmosphere. Based on the absorption spectra recorded at different pH values (see Figure 2a and 3a as representative spectra), formation of mono-, bis-, and tris-ligand complexes was found with both iron ions; the obtained overall stability constants (β) are shown in Table 2. The UV–vis spectrophotometry titration data revealed that complex formation reaction with Fe­(III) starts at pH <2, thus spectra were also measured between pH 1 and 2 to obtain the formation constants of the monoligand complexes (in this pH range, the KCl content of the samples was replaced gradually by HCl to keep the ionic strength constant). In the presence of Fe­(III) at pH ca. 8, the absorbance values decrease in the wavelength range of the charge transfer bands associated with the HQ complexes, since hydrolysis suppressed complex formation at alkaline pH. For all the iron complexes, based on spectral characteristics closely resembling those of corresponding HQ (e.g., tris-complex: λ_max_ = 455 and 582 nm) or 8-hydroxyquinoline-5-sulfonate (HQS) complexes,^36–39^ it is suggested that the ligands are coordinated through the (N,O^–^) donor set by forming the following complexes: [M­(LH)], [M­(LH)_2_], [M­(LH)_3_]. The proton in these protonated species can be assigned to the noncoordinating ammonium moiety in the side chain.

**Figure 2:**
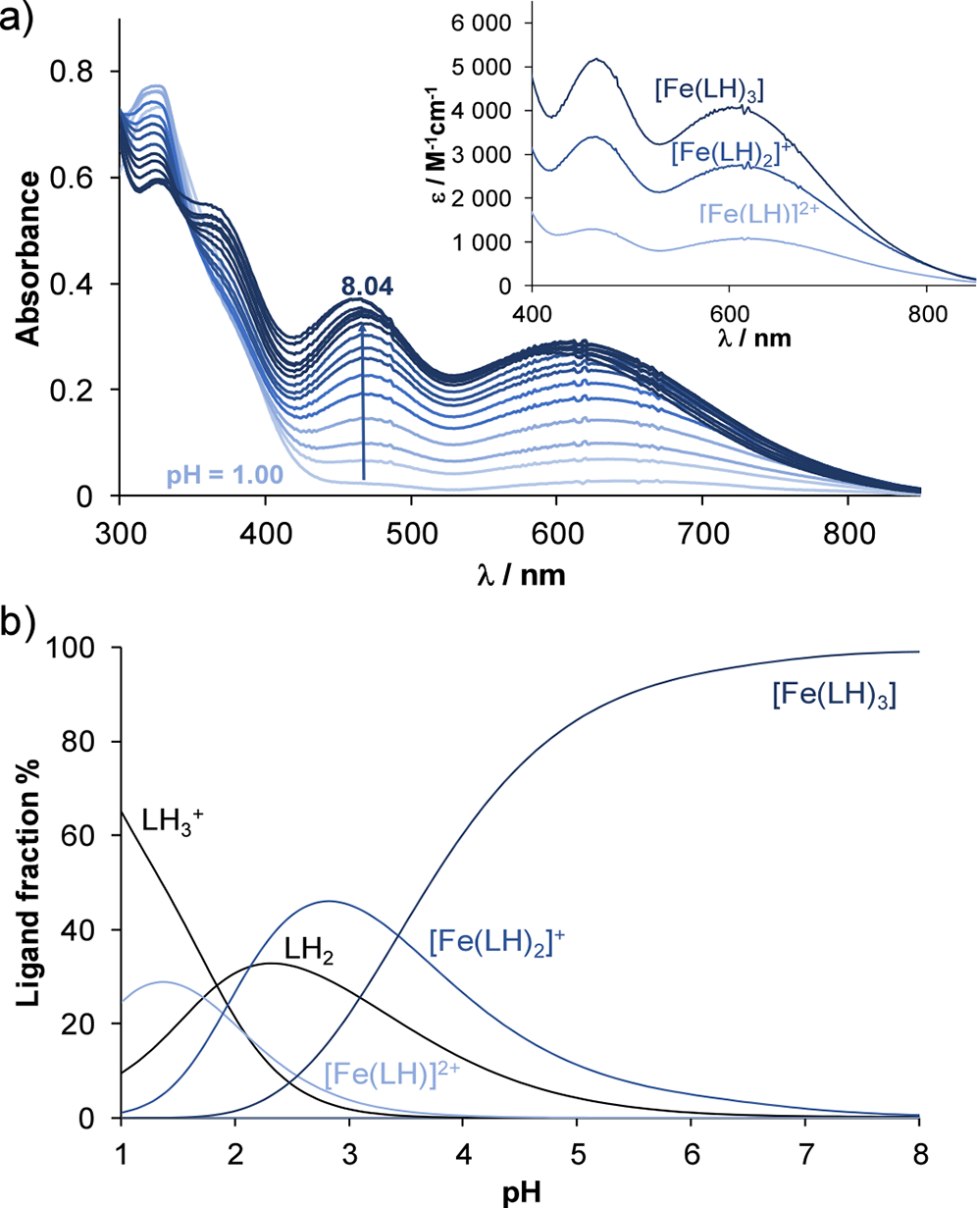
(a) Absorption spectra recorded for the Fe­(III)–HQCl-d-hPro (1:3) system in the pH range 1.0–8.0, and the inserted figure shows the individual UV–vis molar absorption spectra calculated for the complexes. (b) Concentration distribution curves for the same system {*c*_HQCl‑D‑hPro_ = 108 μM; *c*_Fe(III)_ = 35.5 μM; *I* = 0.1 M (KCl); *l* = 2 cm; 30% (*v/v*) DMSO/H_2_O; *T* = 25.0 °C}.

**Figure 3:**
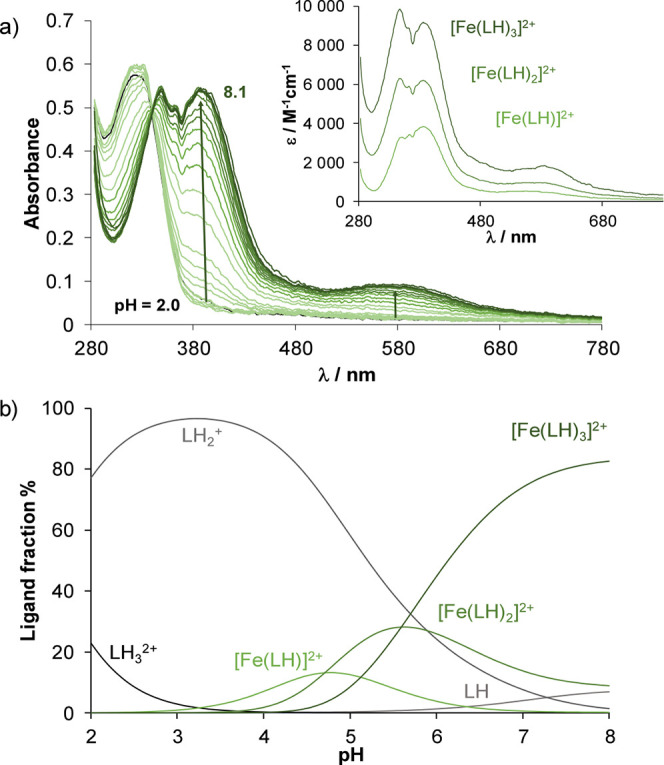
(a) Absorption spectra recorded for the Fe­(II)–HQCl-pip (1:3) system in the pH range 2.0–8.1, and the inserted figure displays the individual UV–vis molar absorption spectra calculated for the different complexes. (b) Concentration distribution curves for the same system {*c*_HQCl‑pip_ = 180 μM; *c*_Fe(II)_ = 58 μM; *l* = 1 cm; *I* = 0.1 M (KCl); 30% (*v/v*) DMSO/H_2_O; *T* = 25.0 °C}.

**Table 2:** Formation Constants (β) of HQCl-L-Pro, HQCl-d-Pro, HQCl-d-hPro, HQCl-Pyr, and HQCl-Pip Complexes Formed with Fe­(III), Fe­(II), and Cu­(II) Obtained by UV–Vis Spectrophotometric Titrations with pM_7.4_ (−log­[M]) Values Calculated at *c*_L_ = 10 μM and *c*_M_ = 1 μM; pH = 7.4^a^

log β	HQCl-l-Pro	HQCl-d-Pro	HQCl-d-hPro	HQCl-pyr^b^	HQCl-pip^b^
[Fe(III)(LH)]	23.28 ± 0.03	23.70 ± 0.03	23.53 ± 0.03	21.64 ± 0.06	21.80 ± 0.03
[Fe(III)(LH)_2_]	45.26 ± 0.06	45.75 ± 0.06	44.93 ± 0.06	42.30 ± 0.04	42.47 ± 0.03
[Fe(III)(LH)_3_]	66.04 ± 0.06	66.01 ± 0.06	65.07 ± 0.09	61.19 ± 0.05	61.67 ± 0.04
[Fe(II)(LH)]	18.61 ± 0.03	18.56 ± 0.03	18.05 ± 0.03	17.11 ± 0.03	17.58 ± 0.03
[Fe(II)(LH)_2_]	36.45 ± 0.03	36.38 ± 0.03	35.74 ± 0.03	34.13 ± 0.03	34.80 ± 0.03
[Fe(II)(LH)_3_]	53.65 ± 0.03	53.75 ± 0.03	52.93 ± 0.03	51.18 ± 0.03	51.55 ± 0.03
[Cu(II)(LH)]	22.51 ± 0.03	22.50 ± 0.03	22.69 ± 0.03	21.53 ± 0.03	21.54 ± 0.03
[Cu(II)_2_(LH)_2_]	–	–	49.53 ± 0.03	–	–
[Cu(II)_2_(LH)_2_(L)]	–	–	68.42 ± 0.03		
[Cu(II)_2_(L)_2_]	38.85 ± 0.03	38.76 ± 0.03	39.23 ± 0.03	–	–
[Cu(II)(LH)_2_]	44.66 ± 0.03	44.49 ± 0.03	44.14 ± 0.15	42.41 ± 0.03	41.72 ± 0.03
[Cu(II)(LH)(L)]	–	–	38.62 ± 0.03		
[Cu(II)(L)_2_]	24.79 ± 0.04	25.81 ± 0.03	28.24 ± 0.03	–	–
[Cu(II)(L)H_–1_]	6.49 ± 0.04	5.95 ± 0.03	6.22 ± 0.03	–	–
pFe(III)^c^	19.5	19.7	19.7	17.4	18.3
pFe(III)*^d^	9.5	9.7	9.7	7.4	8.3
pFe(II)	7.8	8.0	8.0	7.6	8.4
pCu(II)	15.8	15.7	17.5	15.2	14.6

a 30% (*v/v*) DMSO/H_2_O; *I* = 0.1 M (KCl); *T* = 25.0 °C. Charges of the complexes are not shown for clarity. Fully deprotonated form of HQCl-l/d-Pro and HQCl-d-hPro is L^2–^ and HQCl-pyr and HQCl-pip is L^–^.

b Data were evaluated at pH <8.

c pFe value when the Fe­(III) hydroxido species are not considered as unbound species, thus pFe­(III) = −log­[Fe^3+^].

d pFe­(III)* = −log­([Fe^3+^] + [Fe­(OH)]^2+^+[Fe­(OH)_2_]^+^+[Fe­(OH)_3_] + [Fe­(OH)_4_]^−^+2­[Fe_2_(OH)_2_]^4+^).

The concentration distribution curves (Figures 2b and 3b) clearly show that the tris-ligand complexes predominate at pH 7.4 in the case of both metal ions. The log β values (Table 2) are significantly higher for the Fe­(III) than for Fe­(II) complexes and are higher for the amino acid conjugates (HQCl-l/d-Pro, HQCl-d-hPro) than for the ligands without the carboxylate side chain (HQCl-pyr, HQCl-pip). However, direct comparison of these formation constants is inappropriate due to the different basicity of the ligands and the hydrolysis of the metal ions. Thus, pM values (= −log­[unbound metal ion]) were calculated for comparative purposes (often, only the concentration of the aqua complexes of the metal ions is considered when calculating pM; however, in the case of the strongly hydrolyzing Fe­(III), the formed hydroxido species were also included as part of the unbound fraction; see pFe­(III) and pFe­(III)* in Table 2). These data show that the amino acid conjugates have a slightly higher affinity for Fe­(III) than for Fe­(II) at pH 7.4, whereas the pFe­(III) and pFe­(II) values are comparable in the case of HQCl-pyr and HQCl-pip. In contrast, the non-MDR selective HQS (Table S3) binds significantly more weakly Fe­(II) than Fe­(III), indicating a different preference for the iron oxidation states compared to compounds that show greater activity against MDR cells than their parental counterparts.

#### Solution equilibrium of the Cu­(II) complexes of HQ-based Mannich bases

The HQs are efficient binders of Cu­(II) and can act as copper ionophores. Certain MDR-selective derivatives significantly enhance the uptake of coadministered copper in both P-gp-negative MES-SA and P-gp-positive MES-SA/Dx5 and MES-SA/B1 cells, proportionally to their MDR-selective toxicity.^12^ To gain insights into the complexation with Cu­(II), the interaction of the selected five MDR-selective HQ ligands with this metal ion was investigated by UV–vis, electron paramagnetic resonance (EPR), circular dichroism (CD), and electrospray ionization mass spectrometry (ESI-MS). UV–vis spectra recorded for the Cu­(II)–HQCl-d-Pro system are shown in Figure 4. Complex formation reactions with Cu­(II) start at pH <2, thus UV–vis spectra were also measured at pH 1–2, similarly as for Fe­(III). Table 2 summarizes the formation constants, providing the best fits to the experimental data. For HQCl-pyr and HQCl-pip, the speciation models were relatively simple and similar to those of HQ^40^ and HQS,^36^ showing formation of mono- and bis-ligand complexes. However, the amino acid conjugates displayed more complex speciation patterns, including the formation of a dimeric species.

**Figure 4:**
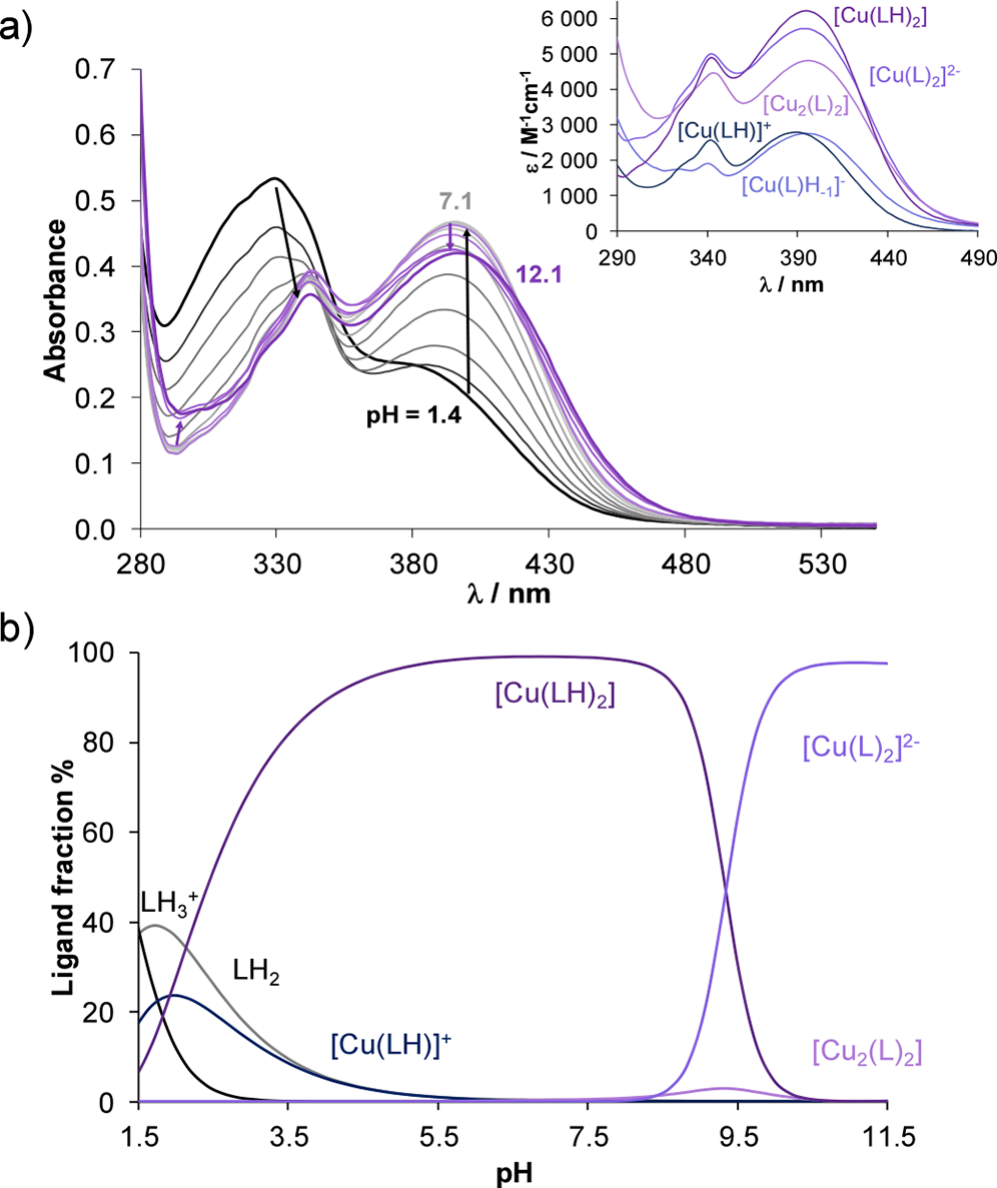
(a) UV–vis spectra measured for the Cu­(II)–HQCl-d-Pro (1:2) system in the pH range 1.4–12.1, and the inserted figure displays the individual UV–vis molar absorption spectra calculated for the various complex species. (b) Concentration distribution curves for the same system {*c*_HQCl‑_ _d_ _‑Pro_ = 50 μM; *c*_Cu(II)_ = 25 μM; *I* = 0.1 M (KCl); *l* = 4 cm; 30% (*v/v*) DMSO/H_2_O; *T* = 25.0 °C}.

In order to confirm the stoichiometry of the copper complexes and to obtain information on their structures, anisotropic (“frozen solution”, 77 K) CW-EPR spectra were recorded for the Cu­(II)–HQCl-pip/pyr systems at different pH values. Similar CW-EPR spectra were recorded in the two systems, which could be simulated by assuming the same component spectra, confirming that different pip/pyr groups do not cause differences in the coordination of Cu­(II). Of the two, the results for the Cu­(II)–HQCl-pip are shown in Figure 5. In these systems, the measured spectra were deconvoluted into the individual spectra of mono complex [Cu­(LH)] and bis-ligand complex [Cu­(LH)_2_] similar to the previously reported Cu­(II)–HQ systems (morpholine (7-(morpholinomethyl)­quinolin-8-ol (Q-2) and piperidine (7-(piperidin-1-ylmethyl)­quinolin-8-ol (Q-3)).^34^ Moreover, a dimeric species [(Cu­(LH)_2_)_2_]^4+^ derived from the corresponding bis-ligand complex (Figures 5c and S1) was also detected at 77 K. The anisotropic EPR parameters of the components are collected in Table 3 and compared with those of previously determined HQ complexes. The very similar EPR parameters to those of other HQ complexes (Q-2, Q-3 in Table 3)^34^ suggest that the side chain nitrogen is not involved in the coordination of Cu­(II), the ligands HQCl-pyr and HQCl-pip are coordinated to Cu­(II) exclusively via (N, O^–^) donor atoms. Also, the very similar Cu–Cu distance of 4.09 Å and the previously detected 3.88 Å for Cu­(II)–Q-2 ^34^ obtained for the dimeric species support that the two copper centers are above and below each other, linked by axial ligation through the phenolato oxygen; thus, a similar structure is proposed for the dimeric species in our case. The doublet peaks observed around 3000 and 3500 G, along with the half-field signal peak near 1500 G (Figure S2), unambiguously point to an *S* = 1 coupled spin system, consistent with interactions between two neighboring Cu­(II) ions. During freezing, the motion of the molecules decreases, allowing these planar aromatic molecules to stack on top of each other through a weaker axial bond. Based on our previous experience, where room-temperature measurements were also possible, this dimer was not detectable in room-temperature solutions. At higher pH values, precipitation occurred at the 1:1 and 1:2 concentration ratios at pH >8, leading to diminished EPR signal intensity. The precipitation was most likely caused by the formation of the neutral [Cu­(L)_2_] complex.

**Figure 5:**
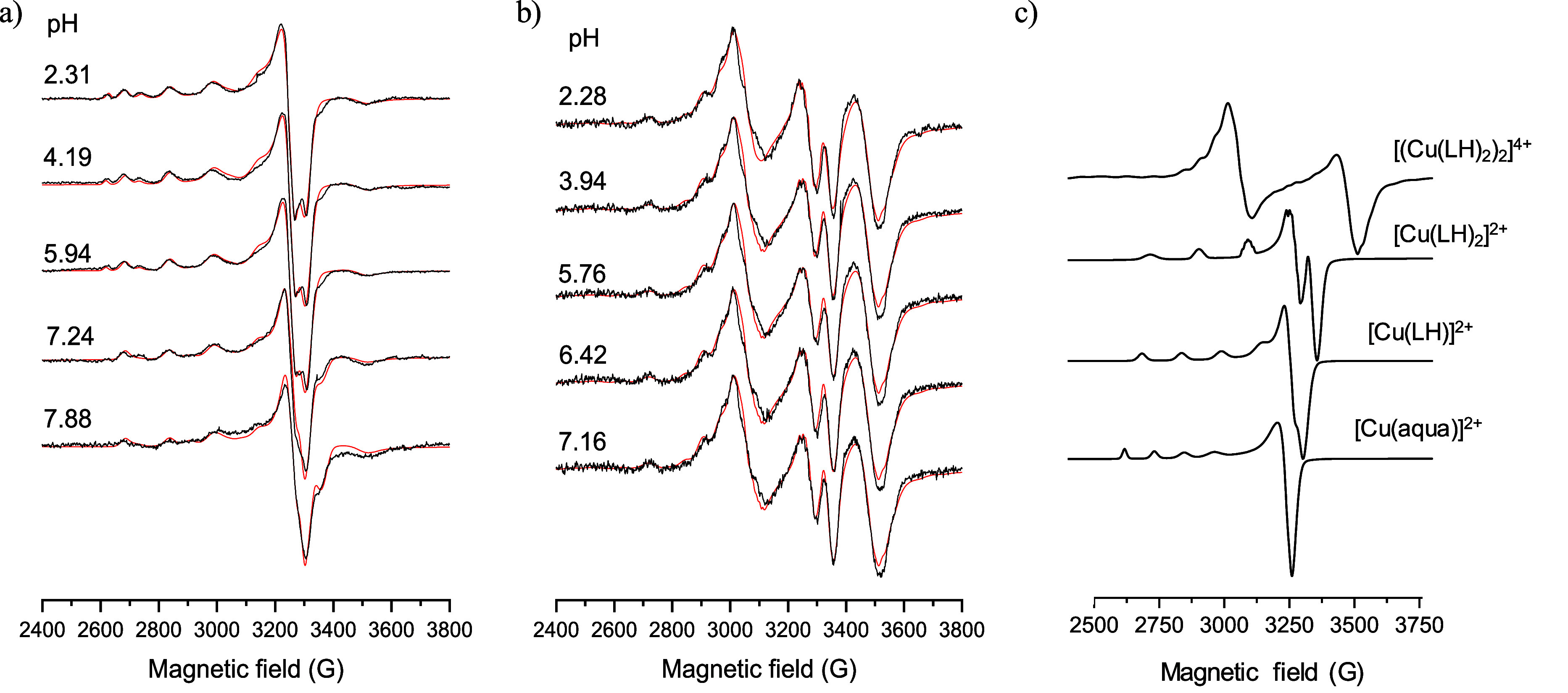
Experimental (black) and simulated (red) frozen solution EPR spectra recorded at different pH values for the Cu­(II)–HQCl-pip system at (a) 1:1 and (b) 1:2 ratios. The spectral intensities were normalized. (c) Component EPR spectra derived from the simulation of frozen solution spectra recorded for the Cu­(II)–HQCl-pip equilibrium system. The EPR spectral parameters are shown in Table 3 {*c*_HQCl‑Pip_ = 200 or 400 μM; *c*_Cu(II)_ = 200 μM; *I* = 0.1 M (KCl); 30% (*v/v*) DMSO/H_2_O; *T* = 77 K}.

**Table 3:** Anisotropic EPR Parameters of the Complexes Determined by Simulating the Frozen Solution EPR Spectra Measured in Cu­(II)–HQCl-Pyr/Pip Solution Equilibrium Systems^a^

	*g*_⊥_	*g*_∥_	*A*_⊥_	*A*_∥_	*a*_0_^N1^, *a*_0_^N2^	*g*_0,calc_ ^b^
[Cu(aqua)]^2+^	2.082	2.415	14	129		2.193
[Cu(LH)]^2+^	2.065	2.313	12	164		2.148
[Cu(LH)_2_]^2+^	2.053	2.248	13	197	9, 13	2.118
[(Cu(LH)_2_)_2_]^4+^ ^c^	2.050	2.240	15	192		2.113
[Cu(Q-3)]^2+^ ^d^	2.051, 2.070	2.287	13, 1	161		2.136
[Cu(Q-2)_2_ ^d^	2.040, 2.044	2.235	27, 5	192		2.106
[(Cu(Q-2)_2_)_2_]^4+^ ^d^	2.023, 2.052	2.220	0, 25	193		2.098

a Coupling values are reported in 10^–4^ cm^–1^ units. Experimental errors: ±0.002 for *g*_⊥_ and ±0.001 for *g*_∥_, ±2 × 10^–4^ cm^–1^ for *A*_⊥_ and ±1 × 10^–4^ cm^–1^ for *A*_∥_.

b Obtained using the equation *g*_0,calc_ = (2*g*_⊥_+*g*_∥_)/3.

c Dimeric signals were described with parameters: *D* = 305 G, *J* >1500 G, Euler angles: 0°,0°,0°, polar angles: χ = 20°, ψ = 0°, calculated Cu–Cu distance: 4.09 Å.

d Data taken from ref 34, parameters for the dimeric species: *D* = 335.5 G, *J* >1500, Euler angles: 0°,3.3°,–5°, polar angles: χ = 28.5°, ψ = −5.0°; Cu–Cu distance: 3.88 Å.

**Table 4:** Anisotropic EPR Spectral Parameters of the Complexes Obtained by Simulating the Frozen Solution EPR Spectra Measured in Cu­(II)–HQCl-l-Pro and Cu­(II)–HQCl-d-hPro Equilibrium Systems^a^

	*g*_⊥_	*g*_∥_	*A*_⊥_	*A*_∥_	*D* ^b^	Cu···Cu(Å)^c^	*g*_0,calc_ ^d^
[Cu(aqua)]^2+^	2.081	2.408	5	128			2.190
[Cu(LH)]^+^	2.068	2.316	11	165			2.151
[Cu(L)]	2.055	2.250	24	184			2.120
[Cu(L)H_–1_]^−^	2.052	2.240	18	188			2.115
[Cu_2_(L)_2_]^e^	2.055	2.281	38	152	30	8.8	2.130
[(Cu(LH)_2_)_2_]^f^	2.055	2.238	14	188	320	4.0	2.116
[(Cu(L)_2_)_2_]^4–^ ^g^	2.050	2.250	19	184	270	4.3	2.117
[Cu_2_(LH)_2_(L)]^h^	2.065	2.065	19	19			2.065

a Coupling values are shown in 10^–4^ cm^–1^ values. The EPR parameters for the same species were identical in both systems. EPR parameters could be determined for complexes [Cu_2_(L)_2_] and [Cu_2_(LH)_2_(L)] only with HQCl-l-Pro, while for [(Cu­(L)_2_)_2_]^4–^, only in the case of HQCl-d-hPro. Experimental errors: ±0.002 for *g*_⊥_ and ±0.001 for *g*_∥_, ±2 × 10^–4^ cm^–1^ for *A*_⊥_ and ±1 × 10^–4^ cm^–1^ for *A*_∥_.

b Dipolar coupling (G).

c Cu···Cu distance calculated by the point-dipol approximation based on dipolar coupling values.

d Calculated by the equation *g*_0,calc_ = (2*g*_⊥_ + *g*_∥_)/3.

e Further simulation parameters: *J* >2000 G, Euler angles: α = γ = 0°, β = 10°, Polar angles: χ = 28°, ψ = 0°.

f Further simulation parameters: *J* >2000 G, Euler angles: α = γ = 0°, −β = 30°, Polar angles: χ = 23°, ψ = 16°.

g Further simulation parameters: *J* >2000 G, Euler angles: α = β = γ = 0°, Polar angles: χ = 35°, ψ = 0°.

h Spectrum described as an isotropic singlet line.

In the case of carboxylate-containing ligands, the corresponding Cu­(II) systems exhibit more composite equilibrium features; thus, more components could be identified based on the frozen solution EPR spectra. The EPR spectra recorded at different pH values together with the simulation curves of Cu­(II)–HQCl-l-Pro and Cu­(II)–-HQCl-d-hPro systems are shown in Figures S3 and S4, respectively. The component spectra obtained by decomposition of the measured spectra are shown in Figure 6. Again, the two systems could be treated with the same component curves for the mono complexes [Cu­(LH)]^+^, [Cu­(L)], and [Cu­(L)­H_–1_]^−^, but there were significant differences in the parameters and structures of their dimeric complexes. When the Cu­(II)–HQCl-l-Pro (1:1) system was investigated, a dimeric [Cu_2_(L)_2_] species was identified (Figure 6b), where both polar angles and Euler angles are very close to zero, and the Cu–Cu distance of 8.8 Å was calculated. It is noteworthy that this Cu–Cu distance is much longer than that found in the dimeric species [(Cu­(LH)_2_)_2_]^4+^ formed in the Cu­(II)-HQCl-pip system, in which the phenolato oxygens act as bridging atoms. This result suggests a different coordination mode.

**Figure 6:**
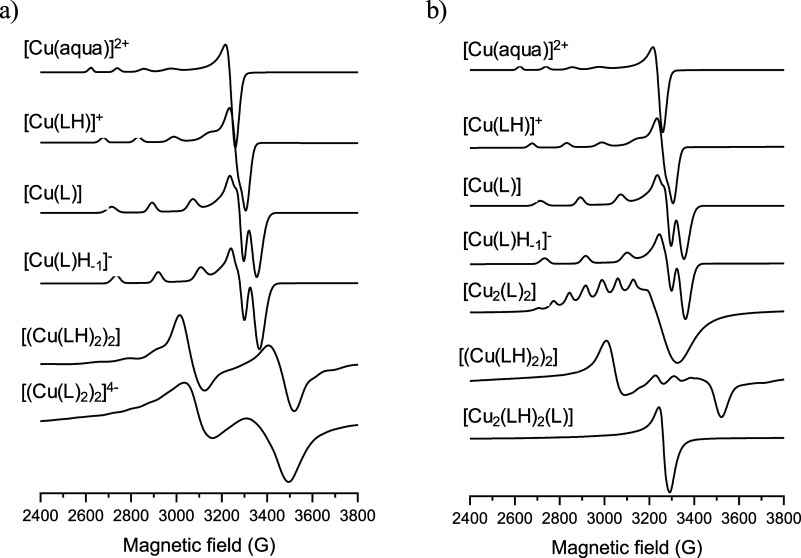
Component spectra calculated by simulating the frozen solution EPR spectra recorded for both the (a) Cu­(II)–HQCl-d-hPro and (b) Cu­(II)–HQCl-l-Pro equilibrium systems. EPR parameters are listed in Table 4.

Therefore, we assume in this case that the carboxylate group of the side chain also participates in bridging the two Cu­(II) centers. In contrast, for the Cu­(II)–HQCl-d-hPro (1:1) system, a decrease in signal intensity was observed at pH >5 without precipitation (Figure S4a). This phenomenon suggests that HQCl-d-hPro forms a dimeric [Cu_2_(L)_2_] complex exhibiting antiferromagnetic exchange coupling and leads to the formation of an EPR-inactive (*S* = 0) species. By recording the anisotropic EPR spectra at 77 K in a 2-fold ligand excess, for both HQCl-l-Pro and HQCl-d-hPro ligands, we were able to identify a dimeric species formed by the weak axial ligation of two bis-ligand complexes as described above for the Cu­(II)–HQCl-pip system (see spectra of [(Cu­(LH)_2_)_2_] and [(Cu­(L)_2_)_2_]^4–^ in Figure 6). Moreover, for HQCl-d-hPro at the 2-fold ligand excess, the EPR spectra indicated the appearance of a new species, which caused the disappearance of the parallel lines in the spectrum. This could be assigned to a remote copper–copper-bonded oligomer, which does not give a well-defined structure. Based on the CD and ESI-MS measurements (*vide supra*), this phenomenon was assigned to the formation of a [Cu_2_(LH)_2_(L)] species.

Since carboxylate-containing ligands have a chiral center, CD spectroscopic titrations were also performed. Spectra obtained for the equimolar Cu­(II)–HQCl-l-Pro system in the pH range 1.4–12.5 display a significant increase in the CD signal at pH 5 (Figure 7). This process may indicate the formation of a complex in which the chiral part of the molecule is involved in the coordination. The findings correlate well with the EPR measurements, since in the equimolar solution, the appearance of a new component was also observed at pH ca. 5 (Figure 6a), where the formation of the dimeric species [Cu_2_(L)_2_] is assumed.

**Figure 7:**
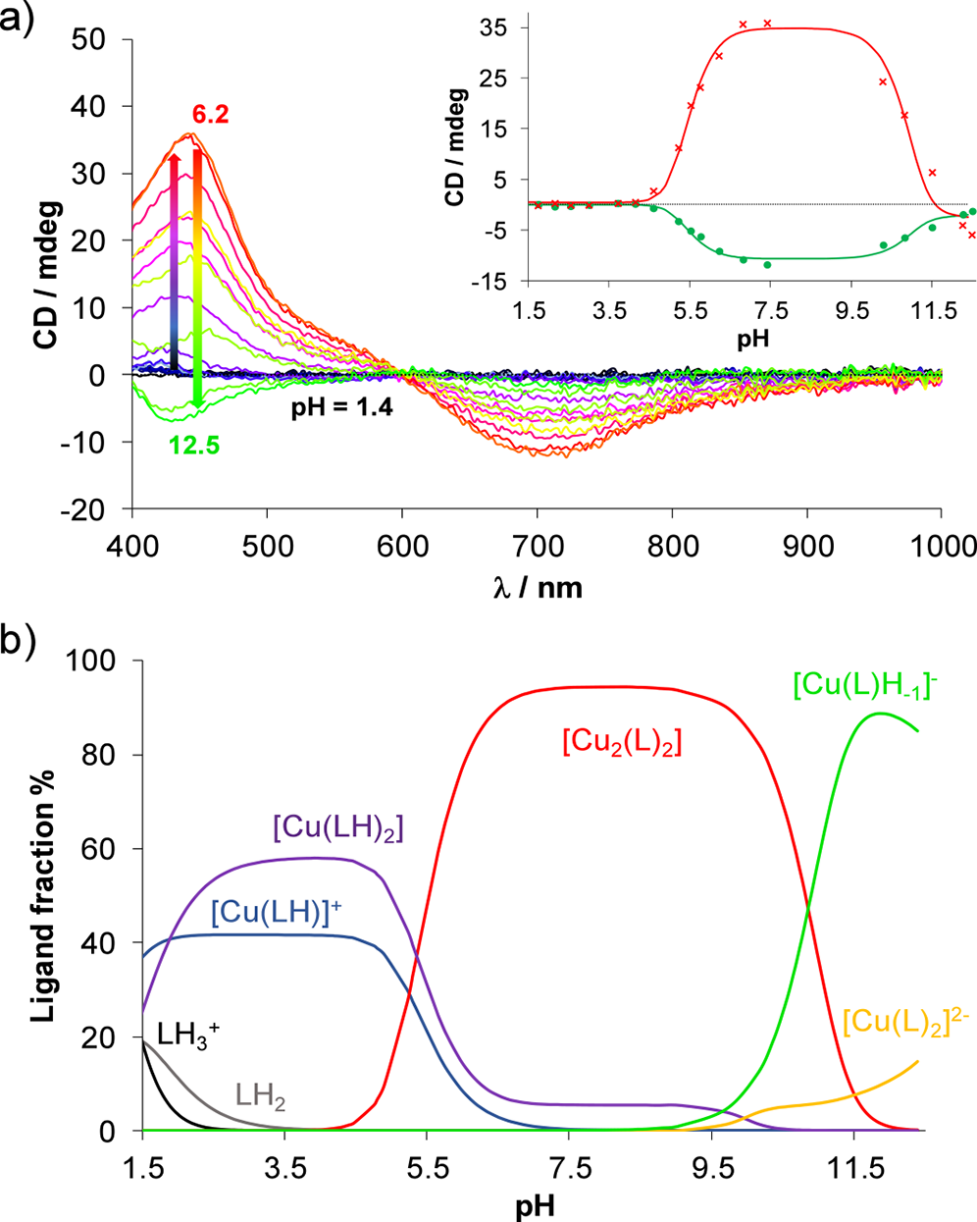
(a) Circular dichroism spectra measured for the Cu­(II)–HQCl-l-Pro (1:1) system in the pH range 1.4–12.5, and the inserted figure represents the measured at 444 (×, red) and 720 (●, cyan) nm together with the fitted values (solid lines). (b) Concentration distribution curves for the same system {*c*_HQCl‑_ _l_ _‑Pro_ = 100 μM; *c*_Cu(II)_ = 100 μM; *I* = 0.1 M (KCl); *l* = 4 cm; 30% (*v/v*) DMSO/H_2_O; *T* = 25.0 °C}.

There is also a difference in the CD spectra of the systems containing HQCl-l/d-Pro and HQCl-d-hPro. We could not describe the Cu­(II)–HQCl-d-hPro system with the same components as for the complexes of HQCl-l-Pro or HQCl-d-Pro. A comparison of HQCl-d-Pro and HQCl-d-hPro in a 1:2 ratio (Figure S5) clearly shows that in the HQCl-d-hPro-containing system, there is at least one more CD-active component. Based on the CD and EPR spectra, after confirming that the amino acid side chain can also coordinate to Cu­(II), we hypothesize a complex structure (in which Cu:L = 2:3) where two mono complexes are linked through a ligand that acts as a bridge between the Cu­(II) centers. To confirm our hypothesis, ESI-MS measurements were carried out. For HQCl-d-hPro, all supposed species were identified at both 1:1 and 1:2 concentration ratios at pH both 4 and 7.4 (Figure S6), and even species with a Cu:L 2:3 ratio were detected, showing a relative abundance of 5–10%. The corresponding *m*/*z* values are collected in Table S4. In contrast, in the Cu­(II)–HQCl-l-Pro system, the presence of this species was only clearly observed at a 1:1 concentration ratio at pH 7.4 (Figure S7). In the sample with a 1:2 metal-to-ligand ratio at pH 7.4, the presence of this dinuclear species cannot be completely excluded due to the quality of the ESI-MS spectra, whereas they are not present at pH 4. This confirms our hypothesis that this dinuclear species is necessary to describe the Cu­(II)–HQCl-d-hPro system, whereas for the Cu­(II)–HQCl-l-Pro system, this was identified only as a minor species. The mass spectra show the presence of the protonated dimeric complex [Cu_2_(LH)_2_]^2+^ in the Cu­(II)–HQCl-d-hPro system. If this species is formed, the presence of the [Cu­(LH)]^+^ species is difficult to determine due to their overlapping formation. The protonated dimer is also observed in Cu­(II)–HQCl-l-Pro (Figure S6) but only to a minor extent. In equimolar systems at pH 7.4, [Cu_2_(L)_2_] is the dominant species in both HQCl-l-Pro and HQCl-d-hPro systems, which is seen in the MS spectra as [Cu_2_(L)_2_H^+^] in Figures S6 and S7.

The UV–vis and CD data were also taken into account in the determination of the speciation model and the overall stability constants of the Cu­(II) complexes of HQCl-l-Pro, HQCl-d-Pro, and HQCl-d-hPro (Table 2). Figure S8 shows a comparison of the different species distributions of Cu­(II)–HQCl-l-Pro and HQCl-d-hPro systems at a 1:1 metal-to-ligand ratio.

Based on the results of spectroscopic and ESI-MS studies and the formation constants determined, we conclude that the bis-Cu complexes of all HQ ligands are the predominant species under biologically relevant conditions (room temperature, in solution at pH 7.4), when the Cu­(II)-to-ligand ratio is 1:2. However, in the equimolar solution, the speciation differs significantly, the dimeric species [Cu_2_(L)_2_] is the predominant species across a broad pH range (6–9.5) in the case of the amino acid conjugates, while HQCl-pyr and HQCl-pip form only monomeric complexes (Figure S9).

The pM values (Table 2) show that the ligands have significantly higher affinity for Cu­(II) compared to Fe­(II) and Fe­(III), and these HQ-based Mannich bases form more stable complexes under the applied conditions than a simple HQ such as HQS (Table S3).

### Density Functional Theory (DFT) Calculations for Selected Dimeric Species

The significant difference obtained in the frozen solution EPR spectroscopic properties of the dimers found in equimolar solution, namely, that an EPR inactive dimer was detected in the case of HQCl-d-hPro, while an EPR-active dimer was found for HQCl-l-Pro, prompted us to investigate and compare their possible structures by DFT calculations. The calculations were performed for the dimeric Cu­(II) complexes formed in the HQCl-l-Pro, HQCl-d-Pro, and HQCl-d-hPro systems to gain insights into the structures of these species. Geometry optimizations were carried out for the protonated dimeric species formed in the Cu­(II)–HQCl-d-hPro (1:1) system ([Cu_2_(LH)_2_]), as well as for the neutral copper­(II) complexes observed in the HQCl-l-Pro, HQCl-d-Pro, and HQCl-d-hPro (1:1) systems ([Cu_2_(L)_2_]).

For the Cu­(II)–HQCl-d-hPro 1:1 system, CD spectroscopy results clearly show the contribution of the chiral moiety of the ligand to the metal binding; therefore, it is plausible that the carboxylate group is involved in coordinating the Cu­(II) ion, while the homoproline nitrogen is protonated in complex [Cu_2_(LH)_2_] in the acidic pH range. Consequently, the formation of three distinct coordination isomers is plausible if the HQ moiety binds via the (N,O^–^) donor set: (i) the carboxylate bridges the copper centers in a monodentate manner; (ii) the carboxylate bridges in a bidentate manner; or (iii) the carboxylate coordinates monodentately, and the phenolato-O of HQ acts as a bridging donor atom, connecting the Cu­(II) centers. The energy values, Cartesian coordinates, and representative structures are reported in the SI (Tables S5–S7 and Figure S10). Based on the relative energies, DFT predicts that the phenolato-bridged dimeric species is more stable than the other two species (with ca. 96.6 kJ/mol). The calculated distance between the Cu­(II) centers is 2.750 Å, which may lead to the conclusion that an EPR-inactive dimeric species is formed in an acidic solution.

For the neutral dimeric species formed in the Cu­(II)–HQCl-l-Pro or HQCl-d-Pro (1:1) systems, we calculated the relative energies of three plausible isomers. In all cases, the (N, O^–^) donor set of the HQ moiety binds to the metal ion, while the carboxylate group coordinates either monodentate or bidentate modes, and the binding of the proline nitrogen is also altered (Figures 8a and S11, Tables S8–S12). In these calculations, the incorporation of phenolate-O as a bridging donor atom was not feasible as optimization of such complexes was not converged. On the basis of relative free energies, the most stable structure in both systems is the complex in which, in addition to the (N, O^–^) coordination of the HQ moiety, the carboxylate group and the proline nitrogen also participate in metal coordination. The distances between the Cu­(II) centers were predicted to be 5.784 Å, confirming the presence of an EPR-active species as it was observed in frozen solution samples. Furthermore, the involvement of the proline nitrogen in the coordination sphere also accounts for the increased CD intensity occurring in the pH range where this complex predominates.

**Figure 8:**
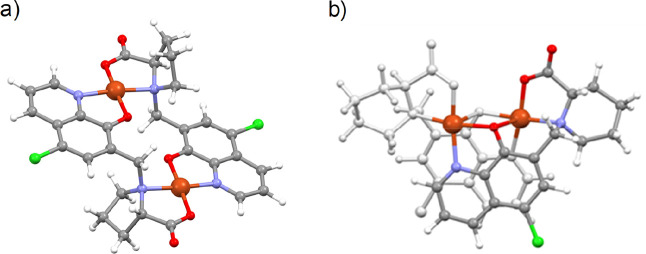
Most stable DFT-calculated structure for the (a) [Cu_2_(HQCl-d-Pro)_2_] and (b) [Cu_2_(HQCl-d-hPro)_2_] dimer. The other structures are shown in Figures S11 and S12.

The neutral complex formed in the 1:1 HQCl-d-hPro system was also studied by using DFT methods. Two plausible isomers were optimized (Figures 8b, S12, Tables S13 and S14); however, the most stable structure is the phenolato-O bridged Cu­(II) complex (Δ*G*_rel_ = −229.2 kJ/mol). In this species, the Cu­(II) centers are located parallel to each other, with one phenolato-O coordinating in the equatorial plane and the other coordinating in the axial position of the Cu­(II). This coordination arrangement combined with the short distance between the metal centers (*d*_Cu(II)–Cu(II)_ = 3.655 Å) results in an EPR-inactive species as it was observed experimentally.

The noted difference between the dimers formed in the HQCl-L/d-Pro and HQCl-d-hPro systems is the presence of phenolate-bridged Cu­(II) species. These were predicted only for the 1:1 Cu­(II)–HQCl-d-hPro 1:1 system. A reasonable explanation is that the incorporation of the homoproline moiety increases the flexibility of the ligand, allowing the simultaneous binding of phenolato oxygen atoms and resulting in a more relaxed structure. In contrast, Cu­(II) dimers of HQCl-L/d-Pro cannot accommodate the phenolato-O as a bridging donor atom, as the 5-membered ring of proline restricts such structural flexibility.

### Structural Studies of HQCl-l-Pro and [Cu­(HQCl-l-ProH_–1_)_2_]

To achieve a deeper structural understanding of the complexes, we made an attempt to obtain single crystals suitable for X-ray crystallographic analysis. Crystals were obtained for the ligand HQCl-l-Pro × MeOH (I) and its Cu­(II) bis-ligand complex [Cu­(HQCl-l-ProH_–1_)_2_] × 3H_2_O (II). Crystal data and structure refinement parameters are listed in Table S15. HQCl-l-Pro crystallized in the orthorhombic crystal system in the *P*2_1_2_1_2 space group with the inclusion of one methanol solvate molecule per asymmetric unit. The ORTEP representation of the complex is depicted in Figure 9a. The unit cell and packing arrangements viewed from the three crystallographic axes are shown in Figures S13 and S14. The proline nitrogen and the hydroxyl group of HQ are protonated, while the carboxylate group of the proline molecule is deprotonated in the crystal structure. The conformation of the molecule is stabilized by an intramolecular hydrogen bond between the proline and HQ parts by C15–H15···O1 (Figure 9). Selected bond lengths and angles together with some torsion angles are listed in Table S16. Two molecules form pairs by O1–H1O···O3 and vice versa (Figures S14 and S15) and pairs are arranged by π···π stacking interactions between neighboring HQ aromatic rings. HQ nitrogen and the carboxylate oxygen atoms take part as acceptors in hydrogen bonds with the proton of proline nitrogen (N2H2) and methanol (H4O). Details of the main hydrogen bonds are summarized in Table S17.

**Figure 9:**
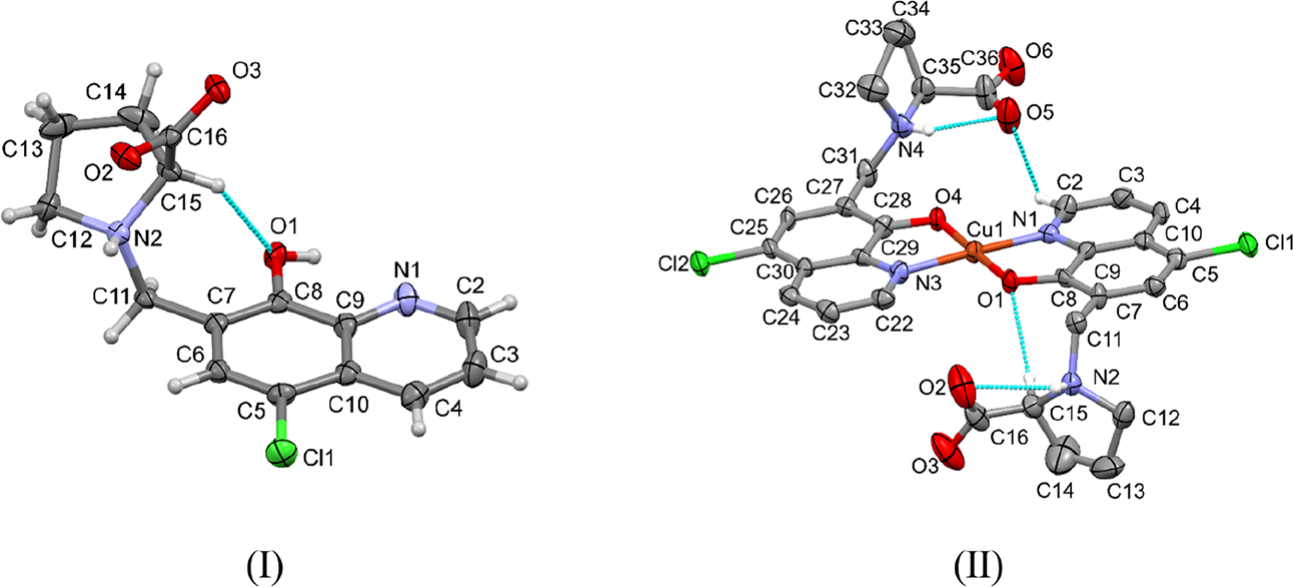
ORTEP representation of structures HQCl-l-Pro × MeOH (I) and [Cu­(HQCl-l-ProH_–1_)_2_] × 3·H_2_O (II) showing atom numbering and intramolecular H-bond connections. Displacement parameters are shown at 50% probability for (I) and 30% for (II). Solvent molecules have been omitted in both structures, and hydrogen atoms are omitted for (II) for clarity.

The bis-ligand complex of HQCl-l-Pro with Cu­(II) resulted in crystal [Cu­(HQCl-l-ProH_–1_)_2_]×3·H_2_O (II) in the *P*1 space group. The unit cell contains one complex in which the two ligands coordinate to the Cu­(II) ion in square planar geometry in a *trans* position via the expected (N, O^–^) donor set and three water molecules of crystallizations (Figure S16). Packing arrangements in crystal (II) viewed from the crystallographic directions “a”, “b”, and “c” are shown in Figure S17. The ORTEP representation of the complex is shown in Figure 9b and the bond lengths and angles of the coordination sphere are collected in Table S18. The conformation of the complex is stabilized by two intramolecular hydrogen bonds (C2–H2···O5 and C15–H15···O1, see Figure 9b). The conformation of the two ligands is not identical, as the proline ring conformation differs considerably, participating in different secondary interactions. Table S16 shows the comparison of bond lengths and angles of the ligand in crystal (I) and the two ligands in crystal (II) and their conformation is compared in Figure S18. The complex molecules are placed below and above each other slightly offset so that their equatorial planes are parallel to each other and the Cl substituent of the HQCl-l-Pro molecule aligned in an axial position to the copper center with a Cu–Cl distance of 3.25 Å (Figure S19a). Adjacent molecules are linked together by hydrogen bonds, which are formed directly between the proline moieties or through water molecules (Figure S18 and Table S19). The water molecules of crystallization are placed in voids with a volume of 65.0 Å^3^ which is 8.4% of the unit cell volume, determined by Mercury software (Figure S19b).

### Redox Properties of Iron and Copper Complexes

In addition to solution speciation studies, the redox properties of the iron and copper complexes of Mannich bases were also investigated, as these complexes are expected to be redox active under physiological conditions on the basis of the reported redox potentials of related HQs.^34–36^ First, cyclic voltammetric measurements were carried out at pH 7.4, and different solvents were used depending on the solubility of the complexes, namely, water (for the amino acid conjugates) or DMF/H_2_O solvent mixture (for HQCl-pyr and HQCl-pip). At pH 7.4, it was suggested that all ligands form a tris–ligand complex with both Fe­(II) and Fe­(III) ions. Cyclic voltammograms of the Fe­(III) – HQCl-d-Pro (1:3) system (Figure 10a) display clearly reversible electrochemical processes. Spectroelectrochemical measurements were also performed for the same solution to verify the reversibility of the processes, as the UV–vis spectra measured at different potential values shown in Figure 10b. The formal potential values for iron and copper complexes are collected in Table 5, and for comparison, data for the HQ and HQS are also included. It is important to note that in all systems studied, reversible processes were observed. The formal potential values calculated for the iron­(III)/iron­(II) redox pair are significantly more positive than those of the iron complexes of the reference compounds HQ and HQS.

**Figure 10:**
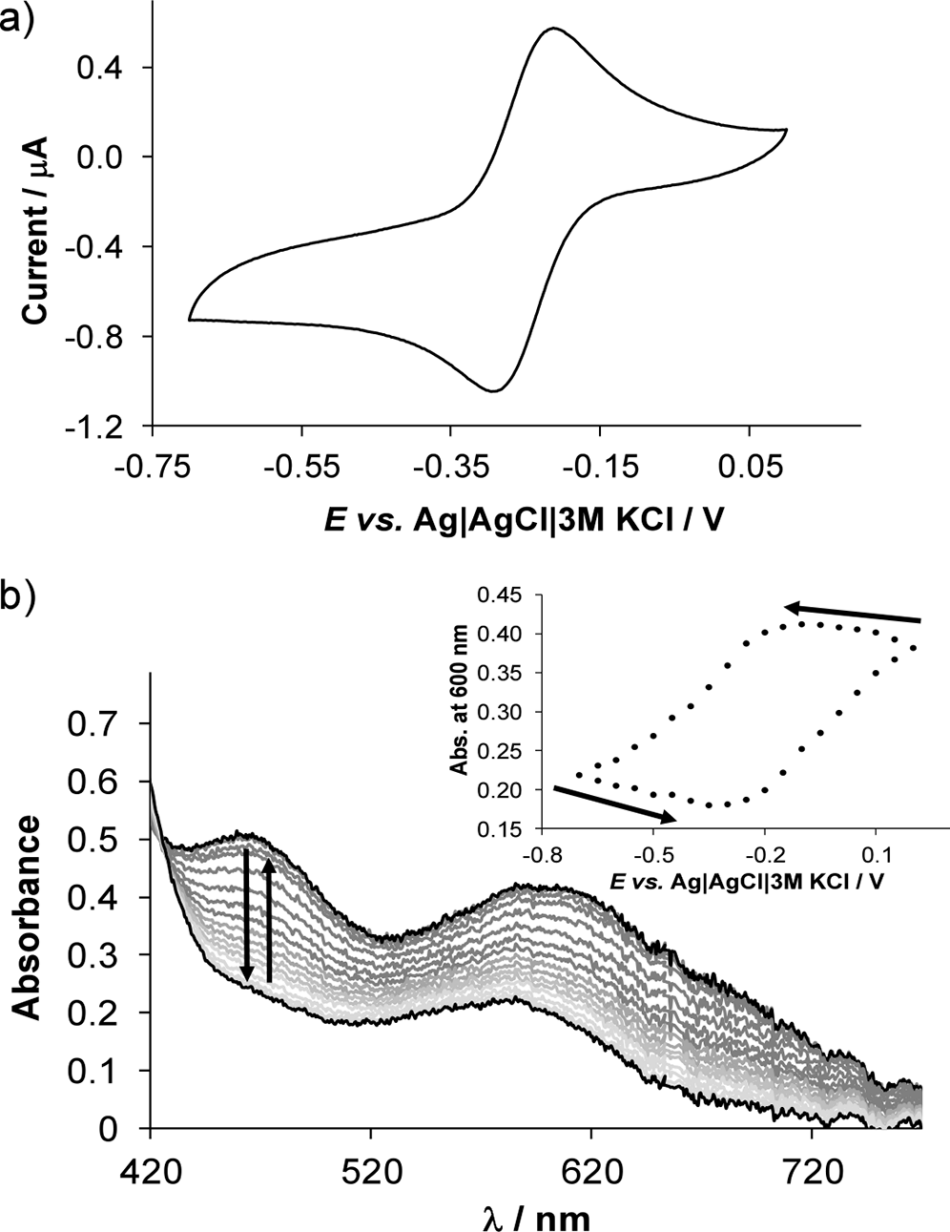
(a) Cyclic voltammogram of the iron­(III)–HQCl-d-Pro (1:3) chemical system. (b) UV–vis absorption spectra measured for the iron­(III)–HQCl-d-Pro (1:3) system at the different potential values using a spectroelectrochemical cell, and the inserted figure displays the absorbance values at 600 nm plotted against the potential {*c*_HQCl‑_ _d_ _‑Pro_ = 1.5 mM; *c*_Fe(III)_ = 0.5 mM; pH = 7.4; scan rate = 10 mV/s; *I* = 0.1 M (KNO_3_); *l* = 1.70 mm; *T* = 25 °C}.

**Table 5:** Electrochemical Data Summarized for the Fe­(III/II)–Ligand (1:3) and Cu­(II/I)–Ligand (1:2) Systems Measured by Cyclic Voltammetry^a^

*E*_1/2_ (Δ*E*)/mV	iron	copper
HQCl-l-Pro	–12 (83)^b^	–
HQCl-d-Pro	–28 (76)^b^	–
HQCl-d-hPro	+6 (85)^b^	–
HQCl-pip	+169 (76)^d^	–205 (88)^d^
HQCl-pyr	+118 (81)^d^	–209 (76)^d^
HQ	–222 (63)^c^	–
HQS	–61 (114)^b^	–350 (81)^d^
	–92 (81)^c^	
	–140 (86)^d^	

a scan rate = 10 mV/s; *c*_M_ = 0.5 mM; *T* = 25 °C; *I* = 0.1 M (KNO_3_); pH = 7.4;.

b Measured in water.

c Measured in 30% (*v/v*) DMF/H_2_O mixture.

d Measured in 60% (*v/v*) DMF/H_2_O mixture.

For the iron complexes formed with HQCl-pyr and HQCl-pip, characterized by outstanding MDR selectivity (Table S1),^29^ more positive redox potential values were obtained, indicating that these ligands have a higher affinity for the +2 oxidation state of iron, as it was also reflected in the more positive difference between pFe­(II) and pFe­(III) values in Table 2 compared to those of the amino acid hybrids. Higher affinity for Fe­(II) might be linked to the mechanism of action underlying MDR-selective toxicity, assuming that such iron complexes are indeed formed in cancer cells.

Based on the Nernst equation, formal potential values could be estimated for the complexes using the formation constants (Table 2) and the standard electrode potential of the aqua complex Fe­(III)/Fe­(II) redox pair.^41^ As shown in Figure 11, the estimated formal potential values are in excellent agreement with the measured data. There is a linear correlation between the formal potentials and the quotient of the overall stability constants of Fe­(III) and Fe­(II) tris-ligand complexes, confirming the correctness of the ratio of these equilibrium constants. Two other HQ derivatives, HQS and (2S)-1-((8-hydroxy-5-nitroquinolin-7-yl)­methyl)­pyrrolidin-1-ium-2-carboxylate (HQNO_2_–Pro), which is also a Mannich base, were also included in the analysis. For those ligands showing lower affinity for Fe­(III) ions based on determined overall stability constants, more positive formal potential values were measured.

**Figure 11:**
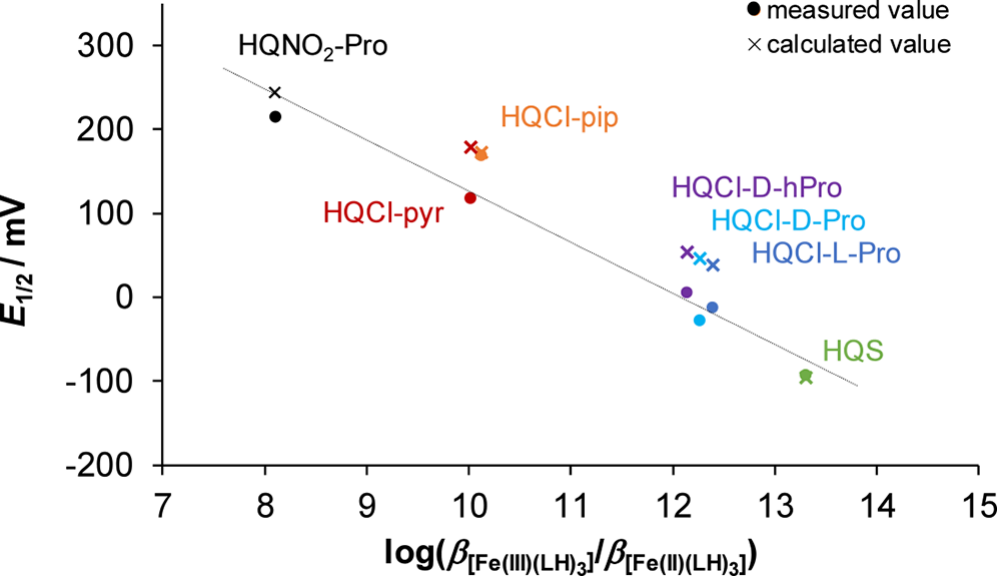
*E*_1/2_ values plotted against log­(β [Fe­(III)­(LH)_3_]/β [Fe­(II)­(LH)_3_]) for the iron complexes of HQs: HQNO_2_–Pro,^35^ HQCl-pyr, HQCl-pip, HQCl-l-Pro, HQCl-d-Pro, HQCl-hPro, HQS. Formal potential values (×) calculated from stability constants (Table 2), and measured formal potential values (•) are given in Table 5.

Due to the low solubility of the copper complexes HQCl-l-Pro, HQCl-d-Pro, and HQCl-d-hPro, we could not obtain their formal potential values. Comparing the redox potential values obtained for the copper complexes of HQCl-pyr, HQCl-pip, and HQS (Table 5), the higher potential values obtained for the Mannich bases indicate their slightly stronger affinity for Cu­(I) ions compared to HQS.

GSH is a tripeptide (γ-l-glutamyl-l-cysteinylglycine), present at high intracellular concentrations (5 mM), functioning as a low-molecular-mass reducing agent in the human body. GSH plays a key role in protecting cells against free radicals and reactive oxygen species. In order to characterize the interaction of Cu­(II) complexes with GSH, UV–vis absorption spectra were recorded under strictly anaerobic conditions using a tandem cuvette. Spectra were only analyzed at λ >300 nm, where neither the reduced nor the oxidized GSH species exhibit detectable absorbance. Cu­(II)–ligand (1:2) systems were investigated at various GSH concentrations (Figure 12), and in all cases, clear evidence of interaction between GSH and the complexes was observed. A significant and rapid decrease in absorbance at ca. 400 nm was observed for the Cu­(II) complex of HQCl-pyr (Figure 12a), attributed to the reduction of the complex and the subsequent release of the ligand from the resulting Cu­(I) complex, which is replaced by GSH. The redox behavior of the complexes differs depending on the presence of a carboxylate group: for the ligand containing a carboxylate moiety (amino acid hybrids), 10 equiv of GSH is insufficient for complete reduction, whereas in its absence (HQCl-pyr, HQCl-pip), the same amount of GSH is sufficient to fully reduce the complex.

**Figure 12:**
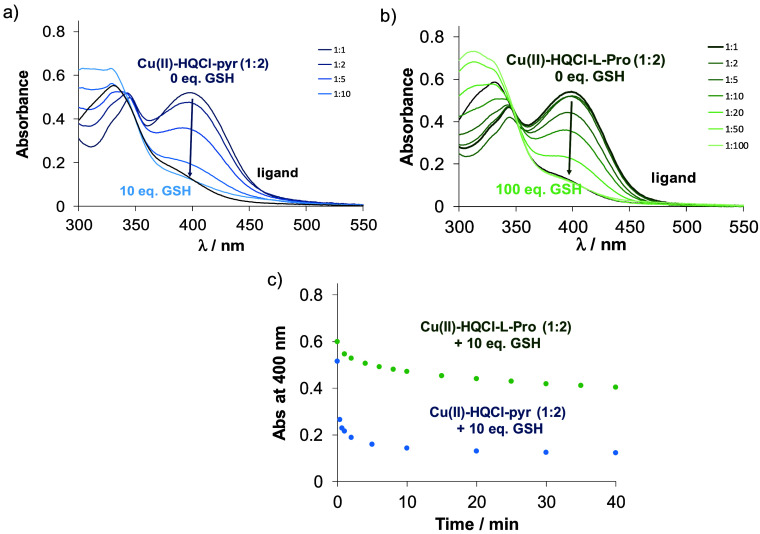
UV–vis spectra of (a) Cu­(II)–HQCl-pyr (1:2) and (b) Cu­(II)–HQCl-l-Pro (1:2) systems with various GSH concentrations, after 40 min reaction time. (c) Absorbance changes at 400 nm on Cu­(II)–HQCl-pyr (1:2) (●, cyan) and Cu­(II)–HQCl-l-Pro (1:2) systems (●, gray) in the presence of 10 equiv of GSH {30% (*v/v*) DMSO/H_2_O; pH = 7.4 (HEPES); *c*_ligand_ = 200 μM; *c*_Cu(II)_ = 100 μM; c_GSH_ = 0 – 10 mM; *l* = 1 cm; *T* = 25.0 °C}.

## Conclusions

The 8-hydroxyquinoline derivatives investigated in this study, containing a methyleneamine subunit at position 7 and a chlorine substituent at position 5, show high anticancer efficacy, particularly against multidrug-resistant cancer cells. Solution phase chemical properties including ligand proton dissociation and lipophilicity, metal binding ability, and the redox properties of their complexes formed in the biofluids can influence the biological activity of these compounds. Complex formation equilibrium processes of the selected HQ derivatives were investigated with Fe­(II), Fe­(III), and Cu­(II), mainly by UV–vis spectrophotometric titrations to achieve a more comprehensive insight into the mechanism of action of these Mannich base derivatives. All the ligands studied form mono-, bis-, and tris-ligand complexes with both Fe­(II) and Fe­(III). Based on the character of the obtained spectra, the ligands coordinate via the (N,O^–^) donor group of the heterocyclic scaffold, with no involvement of the side chain. The overall stability constants of the iron complexes indicate that the tris-ligand complexes are the predominant species at physiological pH. For HQCl-pyr and HQCl-pip, the speciation model for the Cu­(II) complexes turned out to be quite simple. Similar to HQ, these derivatives form mono- and bis-ligand complexes coordinated by the (N,O^–^) donor group. In the frozen state, dimerization of two bis-ligand complexes occurs, linked through the phenolato-oxygen, forming the [(Cu­(LH)_2_)_2_]^4+^ species. More complex equilibrium systems were found for the Cu­(II) complexes of ligands containing carboxyl groups (HQCl-L/d-Pro, HQCl-d-hPro). Through a combination of experimental methods, we provide evidence showing that the carboxylate moiety can be involved in coordination. In addition to the formation of mono- and bis-ligand complexes, these ligands can also form dimeric species [Cu_2_(L)_2_] by bridging two monoligand complexes. Furthermore, they can generate oligomers [Cu_2_(LH)_2_(L)], where two mono complexes are connected by a bridging ligand between the copper centers. The structures of the [Cu_2_(L)_2_] dimer species of the amino acid hybrids as well as for the protonated [Cu_2_(LH)_2_] complex of HQCl-d-hPro were corroborated by DFT calculations. We observed that in an equimolar solution, the speciation is different for the ligands depending on the presence of the carboxylate moiety. The formation of the dimeric species [Cu_2_(L)_2_] predominates at pH 7.4 in the case of the amino acid conjugates, while HQCl-pyr and HQCl-pip form only monomeric complexes. In contrast, when the Cu­(II)-to-ligand ratio is 1:2, all of the ligands studied form a bis-ligand complex as the major species at pH 7.4.

Comparison of the complex stability based on the calculated pM values reveals that the ligands exhibit a preference for Cu­(II) among the metal ions studied. Ligands containing carboxyl groups show a higher affinity for Fe­(III) over Fe­(II), whereas those with increased MDR selectivity, such as HQCl-pyr and HQCl-pip, lacking carboxylate side chains show a higher preference for Fe­(II).

The redox behavior of the iron and copper complexes was characterized by cyclic voltammetry and spectroelectrochemical measurements. A higher preference of HQCl-pyr and HQCl-pip for Fe­(II) is also shown by the more positive redox potentials obtained for the iron complexes, which may contribute to their strong MDR-selective toxicity. The analysis of the copper complexes of HQCl-pyr, HQCl-pip, and HQS revealed the higher potential values obtained for the Mannich bases, indicating that they have a slightly stronger affinity for Cu­(I) than HQS. The reaction of Cu­(II) complexes with the natural reducing agent GSH shows distinct differences between the ligands. In all cases, spectral changes indicate that GSH can reduce the complex, releasing the ligand from the Cu­(I) complex. For the Cu­(II) complexes of HQCl-pyr and HQCl-pip, 10 equiv of GSH is sufficient for the reduction, whereas the carboxyl-containing ligands require a minimum of ca. 100 equiv. This pronounced difference indicates that the presence of a carboxylate group significantly stabilizes the Cu­(II) oxidation state against reduction by glutathione. Our findings highlight the critical influence of ligand design on both the metal binding properties and the MDR-selective toxicity of HQ-based compounds.

## Experimental Section

### Chemicals

GSH, DMF, DMSO, TBACl, glutathione, and 4,4-dimethyl-4-silapentane-1-sulfonic acid (DSS) were purchased from Sigma-Aldrich in puriss quality. KOH, KCl, HCl, KNO_3_, FeCl_3_, CuCl_2_, and potassium hydrogen phthalate were obtained from Reanal (Budapest. Hungary) and used without any further purification steps. The synthesis of HQ derivatives, namely, HQCl-pyr, HQCl-pip, HQCl-l/d-Pro, and HQCl-d-hPro, is described in our previous works.^29,31,32^ Stock solutions of copper­(II) and iron­(III) were prepared by dissolving the required quantities of the respective metal chlorides in a HCl solution. Their metal concentrations were determined by EDTA titration through a complexometric analysis. The iron­(II) stock solution was produced by dissolving fine iron powder in HCl under rigorously oxygen-free argon. After dissolution, the solution was filtered and subsequently handled and stored exclusively under anaerobic conditions inside a glovebox (GP­(Campus) Jacomex, O_2_ content ≤ 1 ppm). The absence of iron­(III) contamination was verified using KSCN. The iron­(II) concentration was quantified by permanganometric titrations under acidic conditions. The strong acid content of each metal stock solution was determined by pH-potentiometric titrations. Analytical-grade solvents were used and were applied without additional purification. Milli-Q-grade water was used for preparing all of the solutions.

### pH Potentiometry

pH-potentiometric measurements were performed to determine the p*K*_a_ values of the ligands in a 30% (*v/v*) DMSO/H_2_O solvent mixture. The measurements were carried out using carbonate-free 0.10 M KOH solution, dissolved in a 30% (*v/v*) DMSO/H_2_O mixture at 25.0 ± 0.1 °C. During the titrations, ionic strength was 0.10 M (KCl). The exact concentrations of KOH and HCl were established by pH-potentiometric titrations. Titrations were carried out using a Metrohm 665 Dosimat buret and an Orion 710A pH-meter equipped with a Metrohm combined electrode (type 6.0234.100). The electrode assembly was standardized on the pH = −log­[H^+^] scale through blank titrations of HCl with KOH, following the protocol of Irving et al.^42^ The mean value obtained for the water ionization constant (p*K*_w_) was 14.53 ± 0.05.^43^ The pH-potentiometric titrations were conducted over the pH interval 2.0–12.5. Prior to each titration, the solutions were purged with purified argon for approximately 10 min to remove the dissolved oxygen. Each sample had an initial volume of 10.0 mL and contained the ligand at a concentration of 4 mM. The acceptable deviation between the experimental and fitted titration curves was kept below 0.01 mL. The exact ligand stock concentration together with the p*K*_a_ values were determined by pH-potentiometric titrations using the computer program HYPERQUAD.^44^

### UV–vis Spectrophotometry and Circular Dichroism Spectroscopy

An Agilent Cary 8454 diode array spectrophotometer was employed to measure the UV–vis spectra at an interval of 200–800 nm. We also used the Thermo Evolution 220 between 190 and 1100 nm range. The path length was 1, 2, or 4 cm. Spectrophotometric titrations were carried out in 30% (*v/v*) DMSO/H_2_O using ligand solutions in the 40–200 μM concentration range. Measurements were performed between pH 1.5 and 12.5, both for the free ligands and in the presence of 1.0, 0.5, or 0.33 equiv of copper­(II), iron­(III), or iron­(II) ions. Experiments involving iron­(II) were conducted inside a laboratory glovebox under strictly anaerobic conditions (O_2_ content ≤ 1 ppm). In this case, we used an Avantes spectrophotometer with an AvaLight-DHc light source and transmission dip probe. Spectra were also recorded in the pH range 1–2 for samples in which the sum of the concentrations of KCl and HCl was always 0.10 M, and the actual pH was calculated based on the hydrochloric acid concentration. The p*K*_a_, the formation constants (log β­(M_*p*_L_*q*_H_*r*_)), and the individual molar absorbance spectra of the compounds in the different forms were calculated with the computer program HypSpec.^44^ Chemical equations for the overall stability (formation) constants of metal complexes and protonated forms of the ligands are shown in the Supporting Information. Literature data were used for Fe­(III) hydroxido species (logβ for [FeH_–1_] = −2.20, [FeH_–2_] = −5.71, [FeH_–3_] = −12.26, [FeH_–4_] = −21.60, [Fe_2_H_–2_] = −2.91),^45^ and for the Fe­(II) hydroxide complexes (logβ values, MH_–1_ = −9.43, MH_–2_ = −20.73, MH_–3_ = −32.68).^45^

CD spectroscopic measurements were carried out on a JASCO-J-1500 (ABL&E–JASCO Hungary Ltd., Budapest, Hungary) spectrometer 4 cm optical path. Titrations were performed between pH 1.4 and 12.5 in a 30% (*v/v*) DMSO/H_2_O solvent mixture, and concentration of Cu­(II) was between 25 and 100 μM using 1:1 and 1:2 metal-to-ligand ratios in samples.

### Cyclic Voltammetry and Spectroelectrochemistry

Cyclic voltammograms were recorded for the Fe­(III/II) ligand systems in water or in 30 or 60% (*v/v*) DMF/H_2_O and Cu­(II/I) ligand systems in 60% (*v/v*) DMF/H_2_O at 25.0 ± 0.1 °C at pH 7.4 (using HCl and KOH solutions to adjust it) using 0.1 M KNO_3_ as a supporting electrolyte. The metal ion concentration was set to 0.5 mM and metal-to-ligand ratios of 1:3 or 1:2 were applied. Electrochemical measurements were carried out using a conventional three-electrode arrangement under an argon atmosphere with an Autolab PGSTAT 204 potentiostat/galvanostat controlled by Metrohm’s Nova software (version 2.0, Metrohm Autolab B.V.). A glassy carbon electrode served as the working electrode, while platinum and Ag|AgCl|3 M KCl were used as the auxiliary and reference electrodes, respectively. Prior to each measurement, the solutions were deaerated by argon bubbling for approximately 10 min. The electrochemical setup was calibrated using an aqueous K_3_[Fe­(CN)_6_] standard solution (*E*_1/2_ = +0.458 V vs. NHE).^35^

UV–vis spectroelectrochemical experiments were carried out in situ using a spectrometer system (Avantes, Model AvaLight-DHc light source combined with an AvaSpec-UL2048XL-EVO detector). Measurements were performed in a dedicated spectroelectrochemical cell kit (AKSTCKIT3) equipped with a Pt-microstructured honeycomb working electrode (Pine Research Instrumentation). The quartz cell was placed in a cuvette holder and optically coupled to a diode-array spectrometer via fiber-optic connections. Data acquisition and spectral processing were accomplished by using the AvaSoft 8.1.1 software package (Avantes).

### EPR Measurements and Evaluation of the Spectra

All EPR spectra were measured using a BRUKER EleXsys E500 spectrometer (microwave frequency around 9.5 GHz, microwave power 13 mW, modulation amplitude 5 G, and modulation frequency 100 kHz). For all Cu­(II) ligand systems (HQCl-pyr, HQCl-pip, HQCl-l-Pro, and HQCl-d-hPro), 0.2 mM Cu­(II) and 0.2 or 0.4 mM ligand concentrations were used. The titrations were carried out with a KOH solution in 30% (*v/v*) DMSO/H_2_O. Samples of 0.25 mL were transferred into quartz EPR tubes and measured in a dewar containing liquid nitrogen (77 K) to obtain the frozen solution EPR spectra at various pH values. The spectrum of the solvent mixture as a background spectrum was subtracted from the measured spectra to correct the baseline. The frozen solution spectra were simulated by a designated EPR software.^46^ Spectra were simulated using axial *g*-tensor components (*g*_*⊥*_, *g*_∥_) together with the corresponding copper hyperfine coupling parameters (*A*_⊥_^Cu^, *A*_∥_^Cu^). Line width variations were modeled with orientation-dependent parameters (α, β, and γ) according to the expression σ_MI_ = α + β M_I_+γ M_I_^2^, where M_I_ represents the magnetic quantum number of copper­(II). The EPR spectrum of the dimeric species was simulated using a dedicated EPR simulation program^46^ designed for calculating spectra and dynamic nuclear polarization phenomena in coupled-spin systems such as biradicals and paramagnetic dimers. In this approach, the spectrum is obtained by full diagonalization of the two-spin Hamiltonian.^47^ Besides the two identical axial *g*- and copper hyperfine *A*-tensor values, the *D* dipolar interaction and *J* exchange interaction between the two spin centers were calculated. The relative orientation of the two centers was characterized by the three Euler angles (α, β, and γ). The relative position of the two centers is further described by two polar angles (χ, ψ), which defines the position of the connector line between the metal centers in the frame of *g*_1_.^48^ Since a natural CuCl_2_ was applied, the spectra were computed by the summation of spectra ^63^Cu and ^65^Cu weighted by their natural abundances. The hyperfine coupling constants and relaxation parameters were determined and reported in field units (Gauss = 10^–4^ T).

### Crystallization, X-ray Data Collection, Structure Solution, and Refinement

Single crystals suitable for X-ray crystallography (HQCl-l-Pro × MeOH) and [Cu­(HQCl-l-ProH_–1_)_2_] × 3·H_2_O were obtained by a slow vapor diffusion of diethyl ether to a methanolic solution of compounds at low temperature. In the case of the complex, the ligand was dissolved in water with CuCl_2_ at a 1:2 metal-to-ligand ratio, and the pH was set to around 7.0. Slow evaporation at room temperature resulted in blue needle-shaped crystals.

Single crystals were mounted on loops and placed on the goniometer. X-ray diffraction data were collected by using a Rigaku RAXIS-RAPID II diffractometer. Crystal I (HQCl-l-Pro × MeOH) was measured at 143(2) K using Mo–Kα radiation, data for crystal II ([Cu­(HQCl-l-ProH_–1_)_2_] × 3·H_2_O) were collected at 295(2) K using Cu-*K*α radiation. In both cases, numerical absorption correction^49^ was carried out using the program RAPID-AUTO.^50^ Sir2014 ^51^ has been used for structure solution and SHELX6 ^52^ program package under WinGX^53^ or Olex2 ^54^ was used for refinement. The crystal structures were solved using direct methods, and the models were refined by full-matrix least-squares procedures against F^2^. All non-hydrogen atoms were refined with anisotropic displacement parameters. Hydrogen atoms were positioned in calculated geometries and included in the structure–factor calculations, but they were not refined independently. Their isotropic displacement parameters were assigned based on the U­(eq) value of the corresponding bonded atom. Both crystals I and II crystallized in a chiral space group, and the Flack parameter values indicating the absolute configuration were found to be consistent with the expected values based on the synthesis. A summary of data collection and refinement parameters is provided in Table S5. Selected bond lengths and angles of compounds were calculated by PLATON software^55^ and are collected in Tables S6 and S7. The graphical representation and the edition of CIF files were done by Mercury^56^ and PublCif^57^ softwares. The crystallographic data files have been deposited with the Cambridge Crystallographic Database as CCDC 2442499 and 2442500 for crystals (I) and (II), respectively.

### Electrospray Ionization Mass Spectrometry

ESI-MS analyses were carried out on a Micromass Q-TOF Premier (Waters MS Technologies) instrument equipped with an electrospray ion source, operating in both positive and negative mode at a resolving power of 70,000. The copper­(II) concentration in the samples was 50 μM and a metal-to-ligand ratio of 1:1 or 1:2 was used. For samples at pH 7.4, (NH_4_)_2_CO_3_ buffer (*c* = 10 mM) was used (volatile salt); and no background electrolyte was applied.

### Density Functional Theory Calculations

The geometry of the dinuclear Cu­(II) complexes was optimized using Gaussian 16 (rev. B.01)^58^ software at the DFT level of theory. The hybrid B3LYP functional^59,60^ with the D3 version of Grimme’s dispersion (including BJ-damping^61^) was employed to accurately describe noncovalent interactions. This functional was combined with the def2-TZVP basis set. Solvent effects were considered using the Polarizable Continuum Model (PCM)^62^ for water. Frequency calculations at the single-point level were performed on the ground-state geometries employing the same functional and basis-set combination. These calculations confirmed the optimized structures as true minima on the potential energy surface, as no imaginary frequencies were found. The Cartesian coordinates and energy values were generated and reported using ESIgen.^63^

## Supplementary Material







## Notes

Additional experimental (UV–vis, EPR, CD, and ESI-MS) and computational (DFT) details of the studied compounds, and structural characterization of the HQCl-l-Pro and its copper­(II) complex (PDF)
Revised CuHQClPro_3H_2_O (CIF)
Fw_Enyedy (ZIP)
CCDC 2442499 and 2442500 contain the supplementary crystallographic data for this paper. These data can be obtained free of charge via www.ccdc.cam.ac.uk/data_request/cif, or by emailing data_request@ccdc.cam.ac.uk, or by contacting The Cambridge Crystallographic Data Centre, 12 Union Road, Cambridge CB2 1EZ, UK.

## Abbreviations

HQ: 8-hydroxyquinoline
β: overall stability constants
CD: circular dichroism
CSD: Cambridge Structural Database
DFT: density functional theory
DMF: dimethyl-formamide
DMSO: dimethyl-sulfoxide
*E*_1/2_: formal potential
EPR: electron paramagnetic resonance
ESI-MS: electrospray ionization mass spectrometry
GSH: glutathione or γ-l-glutamyl-l-cysteinylglycine
HQCl-pyr: 5-chloro-7-(pyrrolidin-1-ylmethyl)­8-hydroxyquinoline
HQCl-pip: 5-chloro-7-(piperidin-1-ylmethyl)­8-hydroxyquinoline
HQCl-d-Pro: (R)-5-chloro-7-((proline-1-yl)­methyl)­8-hydroxyquinoline
HQCl-l-Pro: (S)-5-chloro-7-((proline-1-yl)­methyl)­8-hydroxyquinoline
HQCl-d-hPro: (R)-5-chloro-7-((homoproline-1-yl)­methyl)­8-hydroxyquinoline
HQNO_2_–Pro: (2S)-1-((8-hydroxy-5-nitroquinolin-7-yl)­methyl)­pyrrolidin-1-ium-2-carboxylate
HQS: 8-hydroxyquinoline-5-sulfonate
*K*_a_: proton dissociation constant
*K*_w_: water ionization constant
MDR: multidrug resistance
P-gp: P-glycoprotein
pH-pot: pH-potentiometry
SC-XRD: single-crystal X-ray diffraction
SR: selectivity ratio
UV–vis: UV–visible.

## References

[1] Miller K. D., Nogueira L., Devasia T., Mariotto A. B., Yabroff K. R., Jemal A., Kramer J., Siegel R. L. (2022). Cancer treatment
and survivorship statistics, 2022. CA A Cancer
J. Clin..

[2] Gottesman M., Fojo T., Bates S. E. (2002). Multidrug
resistance in cancer: role
of ATP–dependent transporters. Nat. Rev.
Cancer.

[3] Szakács G., Paterson J., Ludwig J. A., Booth-Genthe C., Gottesman M. M. (2006). Targeting multidrug resistance in
cancer. Nat. Rev. Drug Discovery.

[4] Türk D., Hall M. D., Chu B. F., Ludwig J. A., Fales H. M., Gottesman M. M., Szakács G. (2009). Identification of Compounds Selectively
Killing Multidrug-Resistant Cancer Cells. Cancer
Res..

[5] Füredi A., Tóth S., Szebényi K., Pape V. F., Türk D., Kucsma N., Cervenak L., Tóvári J., Szakács G. (2017). Identification and Validation of Compounds Selectively
Killing Resistant Cancer: Delineating Cell Line-Specific Effects from
P-Glycoprotein-Induced Toxicity. Mol. Cancer
Ther..

[6] Szakács G., Hall M. D., Gottesman M. M., Boumendjel A., Kachadourian R., Day B. J., Baubichon-Cortay H., Di Pietro A. (2014). Targeting the Achilles heel of multidrug-resistant
cancer by exploiting the fitness cost of resistance. Chem. Rev..

[7] Hall M. D., Brimacombe K. R., Varonka M. S., Pluchino K. M., Monda J. K., Li J., Walsh M. J., Boxer M. B., Warren T. H., Fales H. M., Gottesman M. M. (2011). Synthesis and Structure–Activity Evaluation
of Isatin-β-thiosemicarbazones with Improved Selective Activity
toward Multidrug-Resistant Cells Expressing P-Glycoprotein. J. Med. Chem..

[8] Kruszewski M. (2003). Labile iron
pool: the main determinant of cellular response to oxidative stress. Mutat. Res..

[9] Gupte A., Mumper R. J. (2009). Elevated copper
and oxidative stress in cancer cells
as a target for cancer treatment. Cancer Treat.
Rev..

[10] Torti S. V., Torti F. M. (2020). Iron: The cancer
connection. Mol. Aspects Med..

[11] Cserepes M., Türk D., Tóth S., Pape V. F. S., Gaál A., Gera M., Szabó J. E., Kucsma N., Várady G., Vértessy B. G., Streli C., Szabó P. T., Tovari J., Szoboszlai N., Szakács G. (2020). Unshielding
Multidrug Resistant Cancer through Selective Iron Depletion of P-Glycoprotein-Expressing
Cells. Cancer Res..

[12] Pape V. F. S., Gaál A., Szatmári I., Kucsma N., Szoboszlai N., Streli C., Fülöp F., Enyedy E. ´. A., Szakács G. (2021). Relation of Metal-Binding Property and Selective Toxicity
of 8-Hydroxyquinoline Derived Mannich Bases Targeting Multidrug Resistant
Cancer Cells. Cancers.

[13] Pape V. F. S., Palkó R., Tóth S., Szabó M. J., Sessler J., Dormán G., Enyedy E. ´.A., Soós T., Szatmári I., Szakács G. (2022). Structure-Activity
Relationships of 8-Hydroxyquinoline-Derived Mannich Bases with Tertiary
Amines Targeting Multidrug-Resistant Cancer. J. Med. Chem..

[14] Barilli A., Atzeri C., Bassanetti I., Ingoglia F., Dall’Asta V., Bussolati O., Maffini M., Mucchino C., Marchiò L. (2014). Oxidative
stress induced by copper and iron complexes with 8-hydroxyquinoline
derivatives causes paraptotic death of HeLa cancer cells. Mol. Pharmaceutics.

[15] Santini C., Pellei M., Gandin V., Porchia M., Tisato F., Marzano C. (2014). Advances in Copper Complexes as Anticancer Agents. Chem. Rev..

[16] Abdolmaleki S., Aliabadi A., Khaksar S. (2024). Unveiling the promising anticancer
effect of copper-based compounds: a comprehensive review. J. Cancer Res. Clin. Oncol..

[17] Song Y., Xu H., Chen W., Zhan P., Liu X. (2015). 8-Hydroxyquinoline:
a privileged structure with a broad-ranging pharmacological potential. MedChemComm.

[18] Chan S. H., Chui C. H., Chan S. W., Kok S. H. L., Chan D., Tsoi M. Y. T., Leung P. H. M., Lam A. K. Y., Chan A. S. C., Lam K. H., Tang J. C. O. (2013). Synthesis
of 8-Hydroxyquinoline Derivatives
as Novel Antitumor Agents. ACS Med. Chem. Lett..

[19] Oliveri V., Vecchio G. (2016). 8-Hydroxyquinolines
in medicinal chemistry: A structural
perspective. Eur. J. Med. Chem..

[20] Ding W., Lind S. E. (2009). Metal ionophores
– An emerging class of anticancer
drugs. IUBMB Life.

[21] Dömötör O., Pape V. F. S., May N. v., Szakács G., Enyedy É. A. (2017). Comparative solution equilibrium studies of antitumor
ruthenium­(η^6^-*p*-cymene) and rhodium­(η^5^ -C_5_Me_5_) complexes of 8-hydroxyquinolines. Dalton Trans..

[22] Havrylyuk D., Howerton B. S., Nease L., Parkin S., Heidary D. K., Glazer E. C. (2018). Structure-activity relationships of anticancer ruthenium­(II)
complexes with substituted hydroxyquinolines. Eur. J. Med. Chem..

[23] Meng T., Tang S.-F., Qin Q.-P., Liang Y.-L., Wu C.-X., Wang C.-Y., Yan H.-T., Dong J.-X., Liu Y.-C. (2016). Evaluation
of the effect of iodine substitution of 8-hydroxyquinoline on its
platinum­(II) complex: cytotoxicity, cell apoptosis and telomerase
inhibition. MedChemComm.

[24] Mohammadi F., Mansouri-Torshizi H. (2020). Five novel palladium­(II) complexes of 8-hydroxyquinoline
and amino acids with hydrophobic side chains: synthesis, characterization,
cytotoxicity, DNA- and BSA-interaction studies. J. Biomol. Struct. Dyn..

[25] Movassaghi S., Hanif M., Holtkamp H. U., Söhnel T., Jamieson S. M. F., Hartinger C. G. (2018). Making organoruthenium complexes
of 8-hydroxyquinolines more hydrophilic: impact of a novel L-phenylalanine-derived
arene ligand on the biological activity. Dalton
Trans..

[26] Timerbaev A. R. (2009). Advances
in developing tris­(8-quinolinolato)­gallium­(iii) as an anticancer drug:
critical appraisal and prospects. Metallomics.

[27] Shaw A. Y., Chang C.-Y., Hsu M.-Y., Lu P.-J., Yang C.-N., Chen H.-L., Lo C.-W., Shiau C.-W., Chern M.-K. (2010). Synthesis
and structure-activity relationship study of 8-hydroxyquinoline-derived
Mannich bases as anticancer agents. Eur. J.
Med. Chem..

[28] Shen A.-Y., Wu S.-N., Chiu C.-T. (1999). Synthesis and Cytotoxicity Evaluation
of Some 8-Hydroxyquinoline Derivatives. J. Pharm.
Pharmacol..

[29] Pivarcsik T., Tóth S., Pósa S. P., May N. V., Kováts E. ´., Spengler G., Kántor I., Rolya A., Feczkó T., Szatmári I., Szakács G., Enyedy É. A. (2024). Organometallic
Half-Sandwich Complexes of 8-Hydroxyquinoline-Derived Mannich Bases
with Enhanced Solubility: Targeting Multidrug Resistant Cancer. Inorg. Chem..

[30] Dömötör O., Pivarcsik T., Yazdi Z. N., Bakos É., Özvegy-Laczka C., Hetényi A., Martinek T., Szatmári I., Tóth S., Szakács G., Borics A., Enyedy É. A. (2025). Comparative study of multidrug resistance-targeting 8-hydroxyquinoline-amino
acid conjugates: anticancer effect, interaction with human serum albumin
and organic anion transporting polypeptides. Eur. J. Pharm. Sci..

[31] Mészáros J. P., Poljarević J. M., Szatmári I., Csuvik O., Fülöp F., Szoboszlai N., Spengler G., Enyedy É. A. (2020). An 8-hydroxyquinoline–proline
hybrid with multidrug resistance reversal activity and the solution
chemistry of its half-sandwich organometallic Ru and Rh complexes. Dalton Trans..

[32] Pivarcsik T., Dömötör O., Mészáros J. P., May N. V., Spengler G., Csuvik O., Szatmári I., Enyedy É. A. (2021). 8-Hydroxyquinoline-Amino Acid Hybrids and Their Half-Sandwich
Rh and Ru Complexes: Synthesis, Anticancer Activities, Solution Chemistry
and Interaction with Biomolecules. Int. J. Mol.
Sci..

[33] Enyedy É. A., Dömötör O., Varga E., Kiss T., Trondl R., Hartinger C. G., Keppler B. K. (2012). Comparative Solution
Equilibrium Studies of Anticancer Gallium­(III) Complexes of 8-Hydroxyquinoline
and Hydroxy­(Thio)­Pyrone Ligands. J. Inorg. Biochem..

[34] Pape V. F. S., May N. V., Gál G. T., Szatmári I., Szeri F., Fülöp F., Szakács G., Enyedy É. A. (2018). Impact of Copper and Iron Binding
Properties on the
Anticancer Activity of 8-Hydroxyquinoline Derived Mannich Bases. Dalton Trans..

[35] Pivarcsik T., Pósa V., Kovács H., May N. V., Spengler G., Pósa S. P., Tóth S., Nezafat Yazdi Z., Özvegy-Laczka C., Ugrai I., Szatmári I., Szakács G., Enyedy É. A. (2023). Metal Complexes of a 5-Nitro-8-Hydroxyquinoline-Proline
Hybrid with Enhanced Water Solubility Targeting Multidrug Resistant
Cancer Cells. Int. J. Mol. Sci..

[36] Kovács H., Jakusch T., May N. V., Tóth S., Szakács G., Enyedy É. A. (2024). Complex Formation of ML324, the Histone
Demethylase Inhibitor, with Essential Metal Ions: Relationship between
Solution Chemistry and Anticancer Activity. J. Inorg. Biochem..

[37] Turnquist T. D., Sandell E. B. (1968). Stability constants
of iron­(III)-8-hydroxyquinoline
complexes. Anal. Chim. Acta.

[38] Gerard C., Chehhal H., Hugel R. P. (1994). Complexes of iron­(III)
with ligands
of biological interest: dopamine and 8-hydroxyquinoline-5-sulphonic
acid. Polyhedron.

[39] Huyen
Vu T., Serradji N., Seydou M., Brémond É., Ha-Duong N.-T. (2020). Electronic Spectroscopic Characterization of the Formation
of Iron­(III) Metal Complexes: The 8-HydroxyQuinoline as Ligand Case
Study. J. Inorg. Biochem..

[40] National Institute of Standards and Technology . NIST Critically Selected Stability Constants of Metal Complexes Database; National Institute of Standards and Technology, U.S. Dept. of Commerce: Gaithersburg, MD, 2004.

[41] Haynes, W. M. Electrochemical Series. In CRC Handbook of Chemistry and Physics, 95th ed.; CRC Press, 2014; pp 5 80–89.

[42] Irving H. M., Miles M. G., Pettit L. D. (1967). A Study
of Some Problems in Determining
the Stoicheiometric Proton Dissociation Constants of Complexes by
Potentiometric Titrations Using a Glass Electrode. Anal. Chim. Acta.

[43] Mészáros J. P., Kovács H., Spengler G., Kovács F., Frank É., Enyedy É. A. (2023). A Comparative Study on the Metal
Complexes of an Anticancer Estradiol-Hydroxamate Conjugate and Salicylhydroxamic
Acid. J. Inorg. Biochem..

[44] Gans P., Sabatini A., Vacca A. (1996). Investigation of Equilibria in Solution.
Determination of Equilibrium Constants with the HYPERQUAD Suite of
Programs. Talanta.

[45] Hydrolysis of Metal Ions; Brown, P. L. , Ekberg, C. , Eds.; Wiley, 2016.

[46] Rockenbauer A., Korecz L. (1996). Automatic
computer simulations of ESR spectra. Appl. Magn.
Reson..

[47] Song C., Hu K.-N., Joo C.-G., Swager T. M., Griffin R. G. (2006). TOTAPOL:
A Biradical Polarizing Agent for Dynamic Nuclear Polarization Experiments
in Aqueous Media. J. Am. Chem. Soc..

[48] Bacher F., Enyedy E. ´. A., Nagy N. V., Rockenbauer A., Bognár G. M., Trondl R., Novak M. S., Klapproth E., Kiss T., Arion V. B. (2013). Copper­(II) Complexes with Highly
Water-Soluble L- and D-Proline–Thiosemicarbazone Conjugates
as Potential Inhibitors of Topoisomerase IIα. Inorg. Chem..

[49] Higashi, T. Numerical Absorption Correction, NUMABS; Rigaku/MSC Inc., 2002.

[50] Rigaku Corporation . RAPID-AUTO, version 3.1.1., Rigaku Corporation, 2011.

[51] Burla M. C., Caliandro R., Carrozzini B., Cascarano G. L., Cuocci C., Giacovazzo C., Mallamo M., Mazzone A., Polidori G. (2015). Crystal Structure Determination
and Refinement via
SIR2014. J. Appl. Crystallogr..

[52] SHELXL-2013 Program for Crystal Structure Solution; University of Göttingen, Germany, 2013.

[53] Farrugia L.
J. (2012). WinGX and
ORTEP for Windows: An Update. J. Appl. Crystallogr..

[54] Dolomanov O. V., Bourhis L. J., Gildea R. J., Howard J. A. K., Puschmann H. (2009). OLEX2: A Complete
Structure Solution, Refinement and Analysis Program. J. Appl. Crystallogr..

[55] Spek A. L. (2003). Single-Crystal
Structure Validation with the Program PLATON. J. Appl. Crystallogr..

[56] Macrae C. F., Edgington P. R., McCabe P., Pidcock E., Shields G. P., Taylor R., Towler M., van de Streek J. (2006). Mercury: Visualization
and Analysis of Crystal Structures. J. Appl.
Crystallogr..

[57] Westrip S. P. (2010). publCIF:
software for editing, validating and formatting crystallographic information
files. J. Appl. Crystallogr..

[58] Frisch, M. J. ; Trucks, G. W. ; Schlegel, H. B. ; Scuseria, G. E. ; Robb, M. A. ; Cheeseman, J. R. ; Scalmani, G. ; Barone, V. ; Petersson, G. A. ; Nakatsuji, H. ; Li, X. ; Caricato, M. ; Marenich, A. V. ; Bloino, J. ; Janesko, B. G. ; Gomperts, R. ; Mennucci, B. ; Hratchian, H. P. ; Ortiz, J. V. ; Izmaylov, A. F. ; Sonnenberg, J. L. ; Williams-Young, D. ; Ding, F. ; Lipparini, F. ; Egidi, F. ; Goings, J. ; Peng, B. ; Petrone, A. ; Henderson, T. ; Ranasinghe, D. ; Zakrzewski, V. G. ; Gao, J. ; Rega, N. ; Zheng, G. ; Liang, W. ; Hada, M. ; Ehara, M. ; Toyota, K. ; Fukuda, R. ; Hasegawa, J. ; Ishida, M. ; Nakajima, T. ; Honda, Y. ; Kitao, O. ; Nakai, H. ; Vreven, T. ; Throssell, K. ; Montgomery, J. A., Jr. ; Peralta, J. E. ; Ogliaro, F. ; Bearpark, M. J. ; Heyd, J. J. ; Brothers, E. N. ; Kudin, K. N. ; Staroverov, V. N. ; Keith, T. A. ; Kobayashi, R. ; Normand, J. ; Raghavachari, K. ; Rendell, A. P. ; Burant, J. C. ; Iyengar, S. S. ; Tomasi, J. ; Cossi, M. ; Millam, J. M. ; Klene, M. ; Adamo, C. ; Cammi, R. ; Ochterski, J. W. ; Martin, R. L. ; Morokuma, K. ; Farkas, O. ; Foresman, J. B. ; Fox, D. J. Gaussian 16. Revision, B.01.; Gaussian Inc.: Wallingford CT, 2016.

[59] Lee C., Yang W., Parr R. G. (1988). Development
of the Colle-Salvetti
Correlation-Energy Formula into a Functional of the Electron Density. Phys. Rev. B.

[60] Becke A. D. (1993). Density-Functional
Thermochemistry. III. The Role of Exact Exchange. J. Chem. Phys..

[61] Grimme S., Ehrlich S., Goerigk L. (2011). Effect of the Damping Function in
Dispersion Corrected Density Functional Theory. J. Comput. Chem..

[62] Tomasi J., Mennucci B., Cammi R. (2005). Quantum Mechanical Continuum Solvation
Models. Chem. Rev..

[63] Rodríguez-Guerra
Pedregal J., Gómez-Orellana P., Maréchal J.-D. (2018). ESIgen:
Electronic Supporting Information Generator for Computational Chemistry
Publications. J. Chem. Inf. Model..

