# Supplementary material for: A Comparative Study on the Iron and Copper Binding Properties of 8‑Hydroxyquinoline-Derived Mannich Bases Targeting Multidrug-Resistance Cancer Cells
Source: ACS Omega. 2026 Jan 6;11(2):2554–69. doi: 10.1021/acsomega.5c06872 (PMC12824963; doi:10.1021/acsomega.5c06872)
Supplement: Supplementary file 1 [file ao5c06872_si_001.pdf]

SUPPORTING INFORMATION

A comparative study on the iron and copper binding properties of 8-hydroxyquinoline derived Mannich bases targeting multidrug resistance cancer cells

Hilda Kovács,[a] Bálint Hajdu,[a] Nóra V. May,[b] Norbert Lihi,[c] István Szatmári,[d] Gergely Szakács,[e,f,g] Éva A. Enyedy,[a*]

[a] *Department of Molecular and Analytical Chemistry, Interdisciplinary Excellence Centre, University of Szeged, Dóm tér 7-8, H-6720 Szeged, Hungary*

[b] *Centre for Structural Science, HUN-REN Research Centre for Natural Sciences, Magyar tudósok körútja 2, H-1117 Budapest, Hungary*

[c] *HUN-REN-DE Mechanisms of Complex Homogeneous and Heterogeneous Chemical Reactions Research Group, Department of Inorganic and Analytical Chemistry, University of Debrecen, Egyetem tér 1., H-4032 Debrecen, Hungary*

[d] *Institute of Pharmaceutical Chemistry and HUN-REN-SZTE Stereochemistry Research Group, University of Szeged, Eötvös u. 6, H-6720 Szeged, Hungary*

[e] *Center for Cancer Research, Medical University of Vienna, Borschkegasse 8a, A-1090 Vienna, Austria*

[f] *Institute of Molecular Life Sciences, HUN-REN Research Centre for Natural Sciences, Magyar tudósok körútja 2, H-1117 Budapest, Hungary*

[g] *National Laboratory for Drug Research and Development, Magyar tudósok körútja 2, H-1117 Budapest, Hungary*

Contents

IC_{50} values on parental and MDR human cancer cell pairs with selectivity ratios SI-3

Overall stability constants (log*β*) of the HQS complexes SI-4

Frozen solution EPR spectra for Cu(II) – HQCl-pyr 1:2 ratios systems SI-4

Frozen solution EPR spectra for Cu(II) – HQCl-pyr/pip systems (half field signal) SI-5

Frozen solution EPR spectra for Cu(II) – HQCl-L-Pro systems SI-5

Frozen solution EPR spectra for Cu(II) – HQCl-D-hPro systems SI-6

CD spectra and concentration distribution curves for Cu(II) – HQCl-D-Pro and HQCl-D-hPro (1:2) systems SI-6

Collection of measured and calculated m/z values SI-7
Mass spectra of Cu(II) – HQCl-D-hPro 1:1 and 1:2 ratio systems SI-8
Mass spectra of Cu(II) – HQCl-L-Pro 1:1 and 1:2 ratio systems SI-8
Concentration distribution curves for Cu(II) – HQCl-L-Pro and HQCl-D-hPro (1:1) systems SI-9
Concentration distribution curves for Cu(II) – HQCl-pyr and HQCl-pip (1:1) systems SI-9
Energy values and Cartesian coordinates SI-9
Crystal data and structure refinement for HQCl-L-Pro and [Cu(HQCl-L-ProH$_{–1}$)$_2$]·3H_2O SI-28
Comparison of selected bond length in crystal (I) and (II) SI-29
Hydrogen-bond geometry of HQCl-L-Pro SI-29
Selected bond lengths (Å) and angles (°) for crystal [Cu(HQCl-L-ProH$_{–1}$)$_2$]·3H_2O SI-30
Hydrogen-bond geometry of [Cu(HQCl-L-ProH$_{–1}$)$_2$]·3H_2O SI-30
Unit cell containing four molecules in the crystal HQCl-L-Pro SI-31
Packing arrangements in crystal HQCl-L-Pro SI-31
Packing arrangements from different view points in crystal HQCl-L-Pro showing the main hydrogen bond connections SI-32
Unit cell in crystal [Cu(HQCl-L-ProH$_{–1}$)$_2$]·3H_2O SI-32
Packing arrangements in crystal [Cu(HQCl-L-ProH$_{–1}$)$_2$]·3H_2O SI-33
Comparison of the conformation of ligand HQCl-L-Pro in its crystals (I) and (II) SI-33
Crystal packing of [Cu(HQCl-L-ProH$_{–1}$)$_2$]·3H_2O SI-34
Equations for stability constant determination SI-34
References SI-35

Table S1. Cytotoxicity expressed as IC_{50} values (μM) measured in triple coculture of parental (MES-SA) and MDR human cancer cell pairs (MES-SA/B1 and MES-SA/Dx5) with selectivity ratios (SR = IC_{50} (chemosensitive) / IC_{50} (MDR)).

	MES-SA		MES-SA/Dx5		MES-SA/B1		SR	SR	Ref.
	**IC_{50}**	**+SD/-SD**	**IC_{50}**	**+SD/-SD**	**IC_{50}**	**+SD/-SD**	**Dx5**	**B1**	
HQCl-pyr[a]	2.94	0.4/0.4	0.25	0.1/0.01	–		11.6	–	[SI1]
HQCl-pip	1.11	0.4/0.3	0.11	0.04/0.03	0.38	0.2/0.1	10.2	2.9	[SI1]
HQCl-L-Pro	31.3	9.9/7.5	5.6	1.5/1.2	18.4	4.5/3.6	5.6	1.7	[SI2]
HQCl-D-Pro	73.6	9.8/8.6	11.6	5.2/3.6	55.0	4.3/4.0	6.4	1.3	[SI2]
HQCl-D-hPro	35.4	7.5/6.2	6.3	2.1/1.6	30.5	5.3/4.5	5.6	1.2	[SI2]

[a] Measured in a coculture of MES-SA and MES-SA/Dx5.

Table S2. *In vitro* cytotoxicity (IC_{50} in μM) determined in Colo205 (parental) and Colo320 (MDR human cancer) cell lines with selectivity ratios (SR = IC_{50} (parental) / IC_{50} (MDR)).

	**Colo205**	**Colo320**	**SR**	**Ref.**
HQCl-pyr	1.82 ± 0.23	0.91 ± 0.19	2.0	[SI1]
HQCl-pip	1.05 ± 0.11	0.43 ± 0.03	2.4	[SI1]
HQCl-L-Pro	20.92 ± 0.80	12.87 ± 0.74	1.6	[SI3]
HQCl-D-Pro	17.0 ± 1.0	13.41 ± 0.79	1.3	[SI4]
HQCl-D-hPro	18.9 ± 1.1	11.9 ± 1.5	1.6	[SI4]

Table S3. Overall stability constants (log*β*) of the HQS complexes formed with Cu(II), Fe(II) and Fe(III) ions determined by pH potentiometric titrations in addition to pM (–log[M]) values computed at c_L = 10 µM and c_M = 1 µM at pH 7.4. Proton dissociation constants of HQS used for the calculations: pK_{a1} = 3.38 ± 0.01 and pK_{a2} = 8.93 ± 0.01. [a] {30% (*v/v*) DMSO/H_2O; *T* = 25.0 °C; *I* = 0.1 M (KCl)}

**log*β***	**Cu(II)**	**Fe(II)**	**Fe(III)**
[M(L)]	11.96 ± 0.04	7.65 ± 0.01	12.55 ± 0.03
$[M(L)_2]$	22.51 ± 0.04	14.26 ± 0.02	23.99 ± 0.05
$[M(L)_3]$	–	18.47 ± 0.07	33.50 ± 0.06
**$pM_{7.4}$**	13.4	7.4	9.4 (19.4[b])

[a] Charges of the complexes are omitted for clarity. [b] The number in the brackets shows the pM value when the Fe(III) hydroxido species are not considered as unbound species.

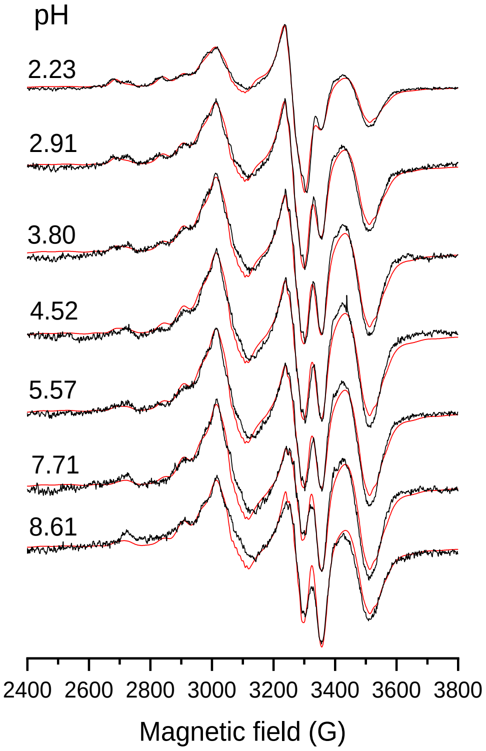

Figure S1. Experimental (black) and simulated (red) 'frozen solution' EPR spectra recorded for Cu(II) – HQCl-pyr system 1:2 ratios at different pH values. The EPR parameters of the spectra are listed in Table 4. {$c_{HQCl\text{-}pyr}$ = 400 µM; $c_{Cu(II)}$ = 200 µM; *I* = 0.1 M (KCl); 30% (*v/v*) DMSO /H_2O; *T* = 77 K}

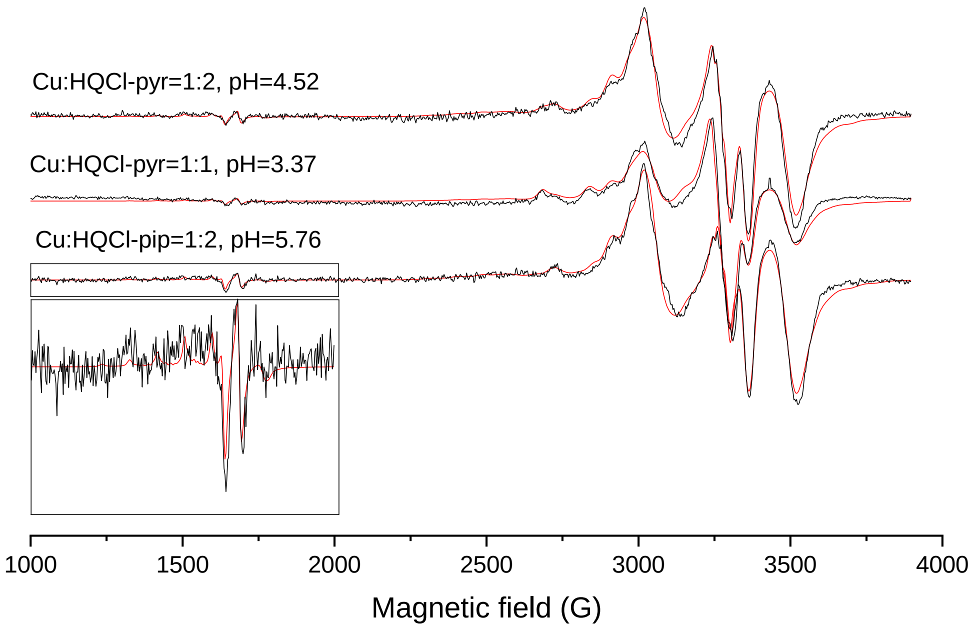

Figure S2. 'Frozen solution' EPR spectra (black) with simulation (red) recorded for Cu(II) – HQCl-pyr/pip systems at different pH and metal-to-ligand ratios showing the half field signal enlarged in the inset. EPR parameters are collected in Table 3.

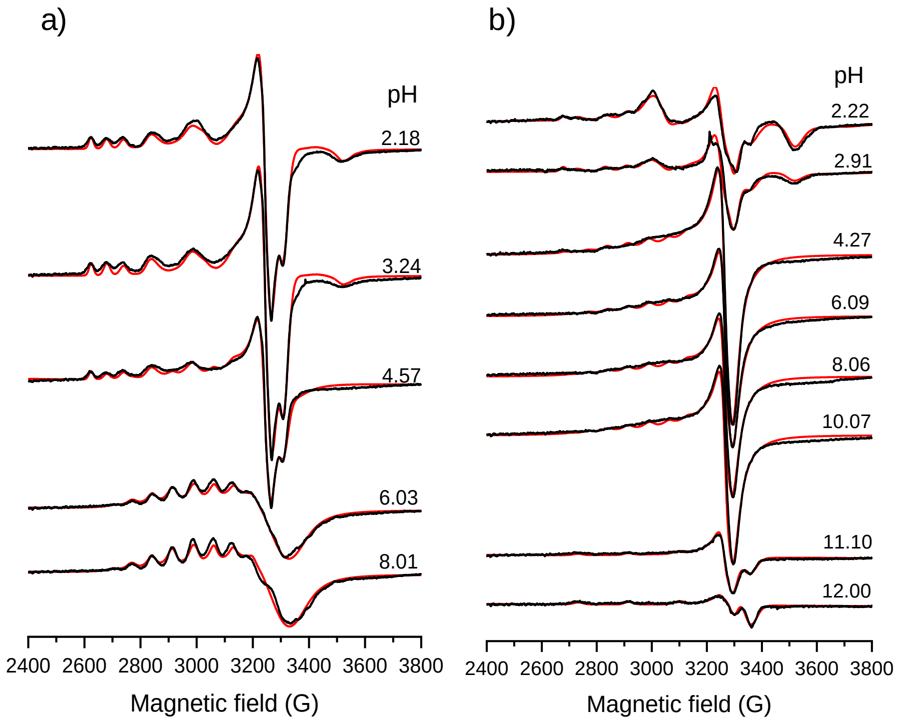

Figure S3. Experimental (black) and simulated (red) 'frozen solution' EPR spectra recorded for Cu(II) – HQCl-L-Pro system (a) 1:1 and (b) 1:2 ratios at different pH values. The EPR parameters of the spectra are listed in Table 4. {$c_{\text{HQCl-L-Pro}}$ = 200 or 400 μM; $c_{Cu(\text{II})}$ = 200 μM; I = 0.1 M (KCl); 30% (*v*/*v*) DMSO /H_2O; T = 77 K}

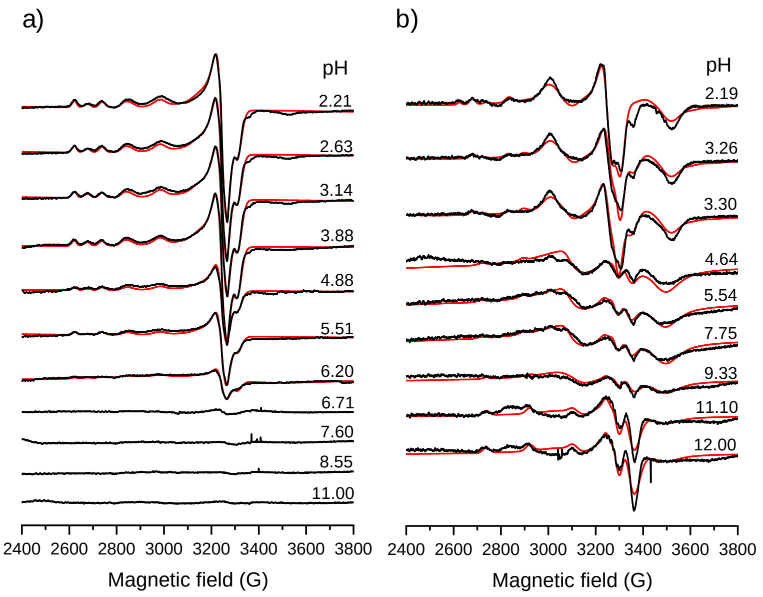

Figure S4. Experimental (black) and simulated (red) 'frozen solution' EPR spectra recorded for Cu(II) – HQCl-D-hPro system (a) 1:1 and (b) 1:2 ratios at different pH values. The EPR parameters of the spectra are listed in Table 4. {$c_{\text{HQCl-D-hPro}}$ = 200 or 400 µM; $c_{Cu(\text{II})}$ = 200 µM; I = 0.1 M (KCl); 30% (v/v) DMSO /H_2O; T = 77 K}

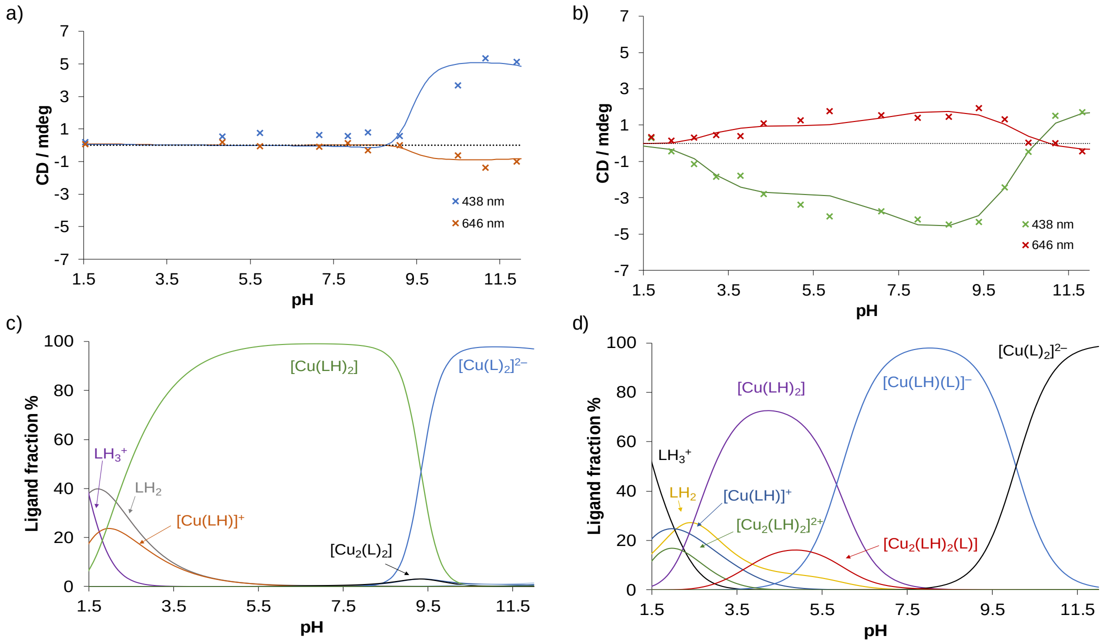

Figure S5. Circular dichroism spectra recorded for the (a) Cu(II) – HQCl-D-Pro (1:2) and (b) Cu(II) – HQCl-D-hPro (1:2) systems in the pH range 1.4 – 12.5. The figure shows the measured CD signals (×) at 438 and 646 nm together with the fitted values (solid lines). Concentration distribution curves calculated for the same systems: (c) Cu(II) – HQCl-D-Pro (1:2) and (d) Cu(II) – HQCl-D-hPro (1:2) systems. {c_{ligandum} = 50 µM; $c_{\text{Cu(II)}}$ = 25 µM; ℓ = 4 cm; I = 0.1 M (KCl); 30% (v/v) DMSO/H_2O; T = 25.0 °C}

Table S4. Collection of measured and calculated m/z values for Cu(II) – HQCl-D-hPro and Cu(II) – HQCl-L-Pro equilibrium systems.

	**HQCL-D-hPro** m/z		**HQCl-L-Pro** m/z	
	calculated	measured	calculated	measured
**LH_3^+**	321.1000	321.0996	307.0839	307.0839
**$[Cu(LH)]^+$**	382.0140	382.0132	367.9983	367.9978
**$[Cu(LH_2)_2]^{2+}$**	352.5558	352.5550	338.5402	338.5395
**$[Cu(LH)_2H]^+$**	704.1044	704.1029	676.0731	676.0721
**$[Cu(LH)(L)]^-$**	700.0922	700.0912	672.0609	672.0603
**$[Cu_2(LH)_2]^{2+}$**	382.0140	382.0132	368.9973	368.9995
**$[Cu_2(L)_2H]^+$**	765.0186	765.0169	736.9873	736.9859
**$[Cu_2(LH)_3H]^{2+}$**	543.0594	543.0586	522.0356	522.0358
**$[Cu_2(LH)_3]^+$**	1085.1115	1085.1087	1043.0644	1043.063
**$[Cu_2(LH)(L)_2]^-$**	1083.0960	1083.0968	–	–

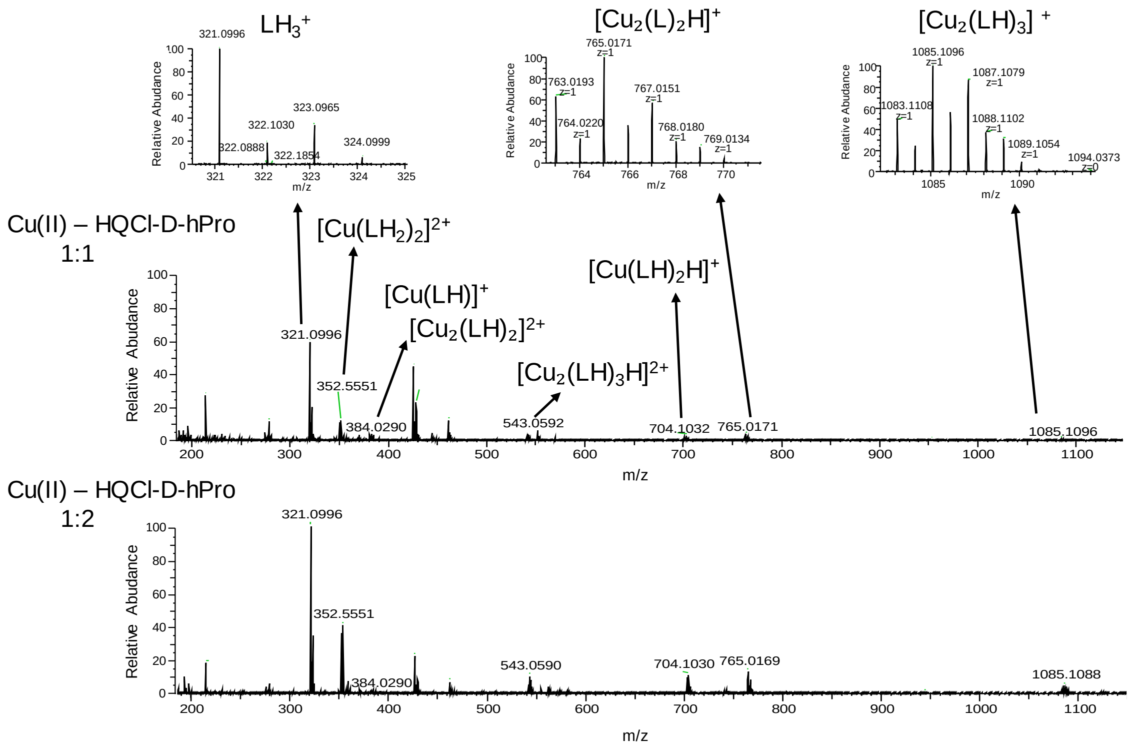

Figure S6. Mass spectra of Cu(II) – HQCl-D-hPro 1:1 and 1:2 systems, the measured isotopic patterns of LH_3^+, $[Cu_2(L)_2H]^+$ and $[Cu_2(LH)_3]^+$ particles shown in the inserted figures. The measured and calculated m/z values are collected in Table S4. {$c_{HQCl\text{-}D\text{-}hPro}$ = 50 or 100 µM; $c_{Cu(II)}$ = 50 µM; pH 7.4 ($(NH_4)_2CO_3$)}

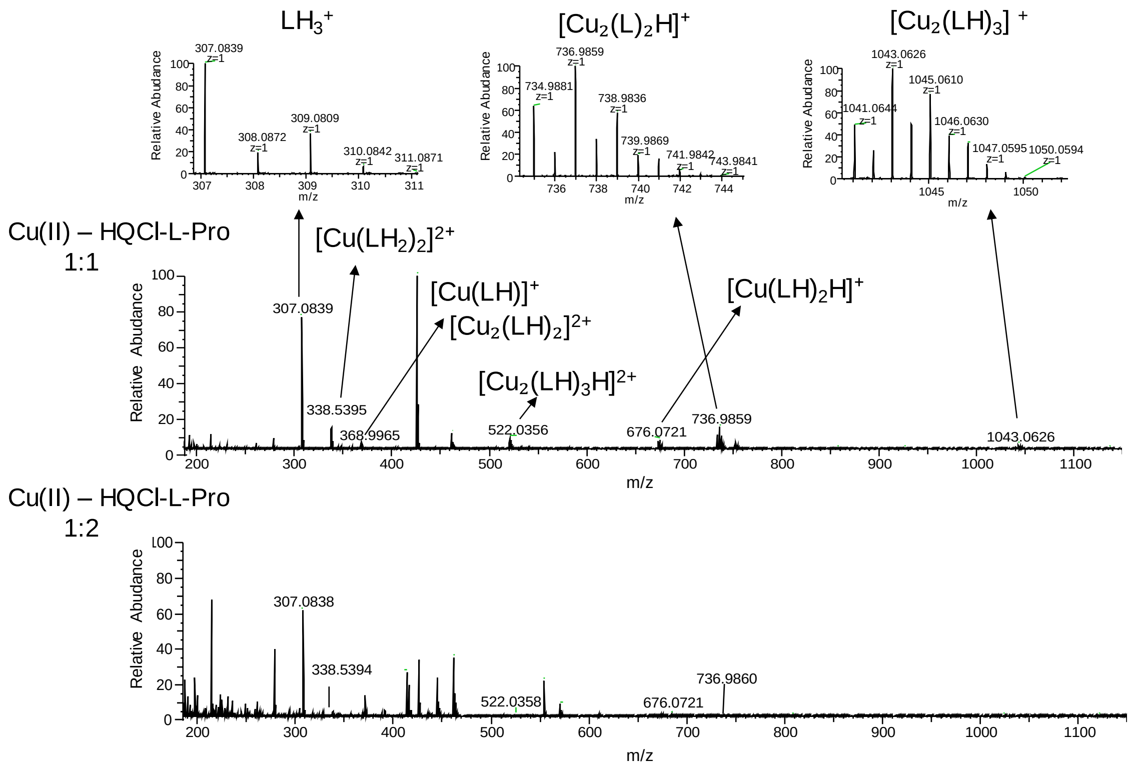

Figure S7. Mass spectra of Cu(II) – HQCl-L-Pro 1:1 and 1:2 systems, the measured isotopic patterns of LH_3^+, $[Cu_2(L)_2H]^+$ and $[Cu_2(LH)_3]^+$ particles shown in the inserted figures. The measured and calculated m/z values are collected in Table S4. {$c_{HQCl\text{-}L\text{-}Pro}$ = 50 or 100 µM; $c_{Cu(II)}$ = 50 µM; pH 7.4 ($(NH_4)_2CO_3$)}

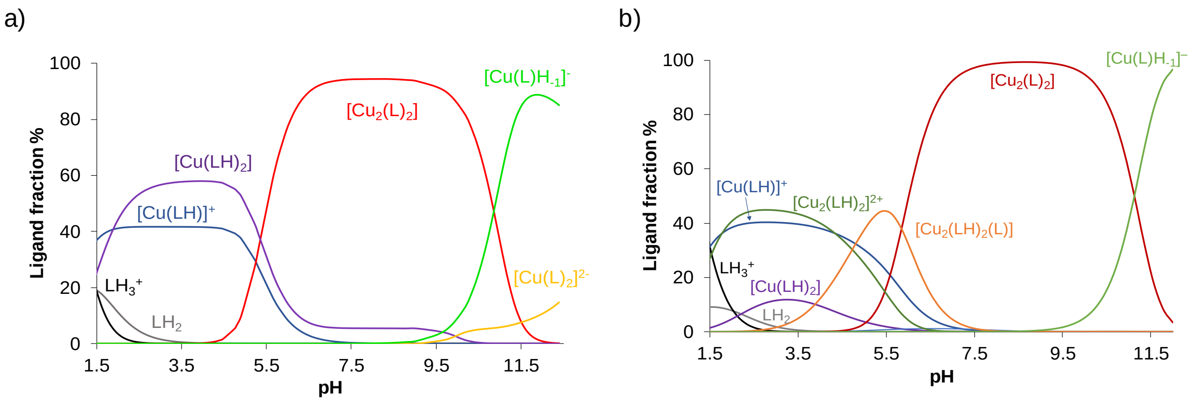

Figure S8. Concentration distribution curves for (a) Cu(II) – HQCl-L-Pro (1:1) and (b) Cu(II) – HQCl-D-hPro (1:1) systems. {c_{ligand} = 50 μM; $c_{\text{Cu(II)}}$ = 50 μM; I = 0.1 M (KCl); 30% (v/v) DMSO/H_2O; T = 25.0 °C}

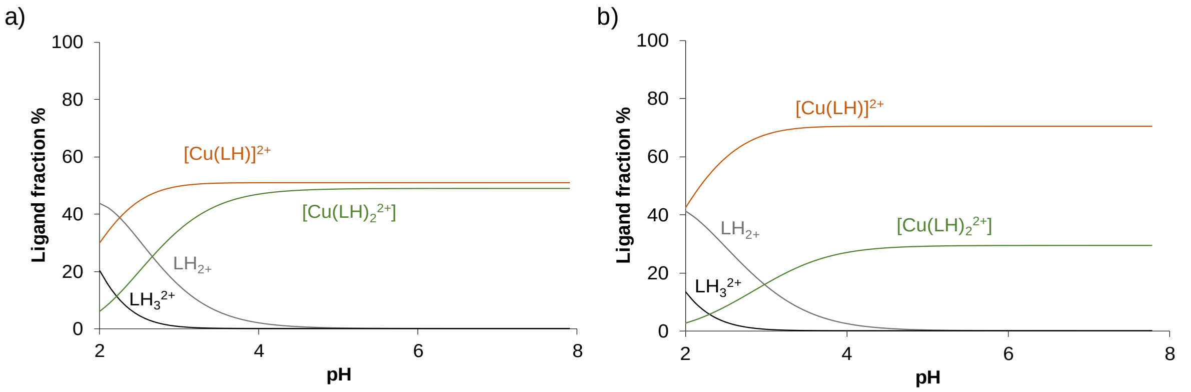

Figure S9. Concentration distribution curves for (a) Cu(II) – HQCl-pyr (1:1) and (b) Cu(II) – HQCl-pip (1:1) systems. {c_{ligand} = 40 μM; $c_{\text{Cu(II)}}$ = 40 μM; I = 0.1 M (KCl); 30% (v/v) DMSO/H_2O; T = 25.0 °C}

Table S5. Energy values and Cartesian coordinates for the [Cu_2(HQCl-D-hProH)$_2$] complex with 2x(N,O) and monodentate carboxylate coordination.

Sum of electronic and zero-point Energies (Eh)	-6110.785902
Sum of electronic and thermal Energies (Eh)	-6110.744776
Sum of electronic and enthalpy Energies (Eh)	-6110.743831
Sum of electronic and thermal Free Energies (Eh)	-6110.861556
Number of Imaginary Frequencies	0

Molecular Geometry in Cartesian Coordinates

Cu	0.123817	-2.768070	1.234738
C	-1.551039	-0.158372	-2.348267
O	-1.627242	1.076992	-1.979905
C	-2.763041	-1.034400	-1.976060

C	-3.257995	-1.913423	-3.116383
C	-4.513962	-2.679454	-2.705143
C	-5.174271	-0.280698	-2.181110
N	-3.869470	-0.120670	-1.440728
O	-0.619891	-0.678472	-2.945422
C	-1.014705	-0.527292	2.397608
C	-1.042374	0.706498	3.071348
C	-2.002945	-0.869491	1.355979
C	-1.996232	1.687179	2.636544
C	-2.887430	0.189145	0.901207
C	-2.890824	1.423512	1.602823
C	0.792707	-1.252235	3.644742
C	0.791665	-0.080369	4.400827
N	-0.079689	-1.462321	2.660998
C	-0.110131	0.914522	4.101437
O	-2.051648	-2.019777	0.894225
Cl	-2.060041	3.219072	3.406322
C	-4.075772	-0.196548	0.050799
Cu	-1.443347	1.471815	-0.010091
C	1.701785	-3.842480	-1.002087
O	1.815873	-4.628152	-1.935871
C	2.984557	-3.117010	-0.550182
C	3.610010	-3.801739	0.659810
C	5.066314	-1.724505	0.644609
C	3.700871	-1.086426	0.786993
N	2.737295	-1.642807	-0.247528
O	0.619476	-3.623283	-0.354037
C	1.087588	2.533843	-0.677799
C	2.056691	3.434727	-1.154266
C	1.329918	1.084849	-0.687926
C	3.249258	2.888720	-1.727262
C	2.521379	0.613849	-1.343882
C	3.444356	1.509643	-1.832786
C	-0.347607	4.255612	-0.116127
C	0.578467	5.216635	-0.524139
N	-0.095157	2.953498	-0.193595
C	1.779684	4.809709	-1.055726
O	0.535574	0.297595	-0.132558
Cl	4.462482	3.931200	-2.326021
C	2.710031	-0.858008	-1.535728
H	-2.465761	-1.673532	-1.147170
H	-2.449229	-2.600555	-3.355373
H	-3.434816	-1.314901	-4.011829
H	-4.251298	-3.380709	-1.911271
H	-4.867201	-3.274766	-3.545957
H	-5.897824	0.369678	-1.696447
H	-4.997395	0.101753	-3.182754
H	-3.646235	2.162809	1.372908
H	1.521560	-2.029878	3.820643
H	1.516966	0.043366	5.191011
H	-0.105004	1.847323	4.645147
H	-4.364896	-1.217395	0.283831
H	-4.908827	0.464867	0.271381
H	3.678684	-3.139776	-1.385669
H	3.670745	-4.867355	0.443187

H	2.940055	-3.696791	1.515456
H	5.444540	-1.557509	-0.365688
H	5.752067	-1.208023	1.314860
H	3.725713	-0.009146	0.658726
H	3.268896	-1.297051	1.761293
H	4.347132	1.150221	-2.306205
H	-1.313816	4.543454	0.273378
H	0.333723	6.264265	-0.433505
H	2.503874	5.532615	-1.400720
H	1.881036	-1.259359	-2.120738
H	3.641249	-1.054843	-2.057560
C	-5.630572	-1.728840	-2.212093
H	-6.496311	-1.767699	-2.872787
H	-5.986915	-2.028867	-1.226551
C	5.000651	-3.227046	0.967884
H	5.756428	-3.756628	0.388021
H	5.227384	-3.394039	2.020938
H	1.789262	-1.567104	0.144860
H	-3.499085	0.818575	-1.644657

Table S6. Energy values and Cartesian coordinates for the [Cu_2(HQCl-D-hProH)$_2$] complex with 2x(N,O) and bidentate carboxylate coordination.

Sum of electronic and zero-point Energies (Eh)	-6110.819428
Sum of electronic and thermal Energies (Eh)	-6110.778659
Sum of electronic and enthalpy Energies (Eh)	-6110.777714
Sum of electronic and thermal Free Energies (Eh)	-6110.896724
Number of Imaginary Frequencies	0

Molecular Geometry in Cartesian Coordinates

Cu	-1.763647	-2.189984	-0.007621
C	-0.419488	3.141029	0.679746
O	0.526474	3.823464	0.200605
C	-1.733324	3.814487	1.020252
C	-1.687439	4.273297	2.499000
C	-3.064100	4.188806	3.147748
C	-3.117999	1.941974	1.938683
N	-2.925693	2.899539	0.785138
O	-0.232217	1.914608	0.925561
C	-4.197883	-1.238513	-0.839468
C	-5.532844	-1.094470	-1.276162
C	-3.351038	-0.118306	-0.598747
C	-6.007238	0.228667	-1.475469
C	-3.859967	1.158012	-0.819264
C	-5.191482	1.310602	-1.258943
C	-4.330379	-3.556153	-0.817996
C	-5.665118	-3.501626	-1.249979
N	-3.627323	-2.457468	-0.620050
C	-6.263014	-2.286457	-1.477193
O	-2.135700	-0.335133	-0.176898
Cl	-7.648062	0.479913	-2.018187
C	-2.922745	2.303552	-0.630310

Cu	1.627942	2.064621	0.168495
C	0.328660	-3.167223	0.558417
O	0.092544	-1.927745	0.668852
C	1.718960	-3.660606	0.894381
C	1.747282	-4.332913	2.266607
C	4.245964	-3.848175	2.171597
C	3.680644	-2.459464	1.952612
N	2.661456	-2.468783	0.837943
O	-0.586673	-3.947750	0.197069
C	4.115349	1.342616	-0.734611
C	5.432794	1.348789	-1.246012
C	3.407611	0.141433	-0.447168
C	6.041020	0.088365	-1.467764
C	4.037873	-1.067783	-0.715323
C	5.357944	-1.073229	-1.210627
C	3.983448	3.663183	-0.747116
C	5.293269	3.756657	-1.242910
N	3.426396	2.493323	-0.502447
C	6.011790	2.613148	-1.490970
O	2.195311	0.226817	0.055779
Cl	7.673459	0.012654	-2.080235
C	3.284924	-2.349135	-0.535481
H	-1.874925	4.663261	0.356051
H	-1.292700	5.286776	2.521216
H	-0.992203	3.640396	3.052641
H	-3.769475	4.826989	2.608427
H	-3.015253	4.584286	4.160993
H	-3.869830	1.222815	1.635084
H	-2.173832	1.427003	2.066679
H	-5.577272	2.306046	-1.434652
H	-3.836179	-4.499916	-0.633831
H	-6.206801	-4.423904	-1.399197
H	-7.289548	-2.234612	-1.810581
H	-1.911816	1.957470	-0.810610
H	-3.151078	3.138393	-1.288390
H	2.039550	-4.369573	0.132951
H	1.029948	-5.151764	2.245481
H	1.395601	-3.624644	3.018286
H	4.735526	-4.186425	1.257096
H	5.029389	-3.775930	2.924583
H	4.443010	-1.739653	1.668853
H	3.173762	-2.083052	2.837974
H	5.841485	-2.022585	-1.397025
H	3.387510	4.543838	-0.550787
H	5.718276	4.732116	-1.427192
H	7.019055	2.672025	-1.877760
H	2.452773	-2.408101	-1.237773
H	3.923479	-3.215000	-0.685991
C	-3.560301	2.733675	3.175761
H	-3.161238	2.221591	4.051231
H	-4.646745	2.715089	3.259155
C	3.151430	-4.845316	2.604753
H	3.316583	-5.804311	2.113706
H	3.203564	-5.023044	3.678466
H	-3.739200	3.514665	0.805670

H	2.072887	-1.622243	0.926959

Table S7. Energy values and Cartesian coordinates for the $[Cu_2(HQCl\text{-}D\text{-}hProH)_2]$ complex with 2x(N,O), monodentate carboxylate and phenolate-O bridging coordination modes.

Sum of electronic and zero-point Energies (Eh)	-6110.824874
Sum of electronic and thermal Energies (Eh)	-6110.784984
Sum of electronic and enthalpy Energies (Eh)	-6110.78404
Sum of electronic and thermal Free Energies (Eh)	-6110.898331
Number of Imaginary Frequencies	0

Molecular Geometry in Cartesian Coordinates

C	2.023709	-2.325812	-0.165603
C	3.076479	-3.127432	-0.658520
N	0.719808	-2.663050	-0.364799
C	2.720354	-4.272252	-1.402325
C	0.407843	-3.743999	-1.051802
C	1.396251	-4.574491	-1.599780
C	3.566948	-0.808010	0.914363
C	4.623936	-1.622352	0.443697
C	2.265002	-1.143046	0.586716
C	4.398133	-2.721179	-0.337662
O	1.191827	-0.450104	0.984795
Cl	5.747702	-3.660104	-0.907811
C	3.826586	0.298349	1.891439
N	4.467717	1.574434	1.373863
C	4.393261	3.647938	-0.027967
C	5.548406	3.970385	0.913428
C	3.614211	2.390117	0.415265
C	6.569120	2.821859	0.926079
C	5.892902	1.441463	0.893981
C	3.194855	1.578787	-0.827530
O	1.931921	1.489767	-1.050256
O	4.059318	1.151281	-1.580454
C	-2.038953	2.348657	-0.189829
C	-3.086689	3.198751	-0.609392
N	-0.744773	2.609910	-0.519691
C	-2.734442	4.330379	-1.374838
C	-0.436133	3.668168	-1.242754
C	-1.419010	4.563091	-1.687865
C	-3.576558	0.864881	0.936608
C	-4.626603	1.721060	0.528403
C	-2.272569	1.176132	0.589065
C	-4.403684	2.840710	-0.222220
Cl	-5.745164	3.843070	-0.694717
C	-3.896030	-0.339606	1.767364
N	-4.774761	-1.355021	1.048910
C	-5.026840	-2.986481	-0.853149
C	-6.339879	-3.275505	-0.134450
C	-4.437593	-1.633582	-0.422253

C	-6.105258	-3.429243	1.379990
C	-4.895094	-2.626314	1.853402
C	-2.936550	-1.594379	-0.688802
O	-2.519472	-1.094739	-1.722890
O	-2.223108	-2.137505	0.241696
Cu	-0.503414	-1.351990	0.463952
Cu	0.465862	1.199364	0.128598
O	-1.195304	0.478717	0.956360
H	3.495474	-4.907790	-1.805215
H	-0.645133	-3.959471	-1.168525
H	1.100742	-5.447114	-2.162816
H	5.640350	-1.380558	0.716090
H	4.500045	-0.048395	2.673005
H	2.895673	0.607184	2.358238
H	4.791740	3.476118	-1.027421
H	3.688234	4.475125	-0.089227
H	5.174323	4.168749	1.922273
H	6.036916	4.889348	0.592993
H	2.744006	2.671547	1.001571
H	7.206128	2.901246	1.806177
H	7.214280	2.895798	0.051118
H	5.838707	1.026247	-0.105597
H	6.386023	0.737877	1.558021
H	-3.507587	5.005795	-1.711099
H	0.609679	3.806449	-1.478838
H	-1.127247	5.421562	-2.274121
H	-5.640760	1.488445	0.824170
H	-4.461764	-0.061778	2.654972
H	-2.998522	-0.860209	2.071830
H	-5.162589	-2.948778	-1.932719
H	-4.309199	-3.781903	-0.645036
H	-6.792226	-4.180622	-0.536234
H	-7.047964	-2.466111	-0.329211
H	-4.900695	-0.823931	-0.978674
H	-5.918133	-4.473155	1.631425
H	-6.993746	-3.120908	1.931228
H	-4.982050	-2.337045	2.897111
H	-3.954544	-3.150100	1.713829
H	-5.708536	-0.946548	1.025872
H	4.520185	2.153145	2.213479

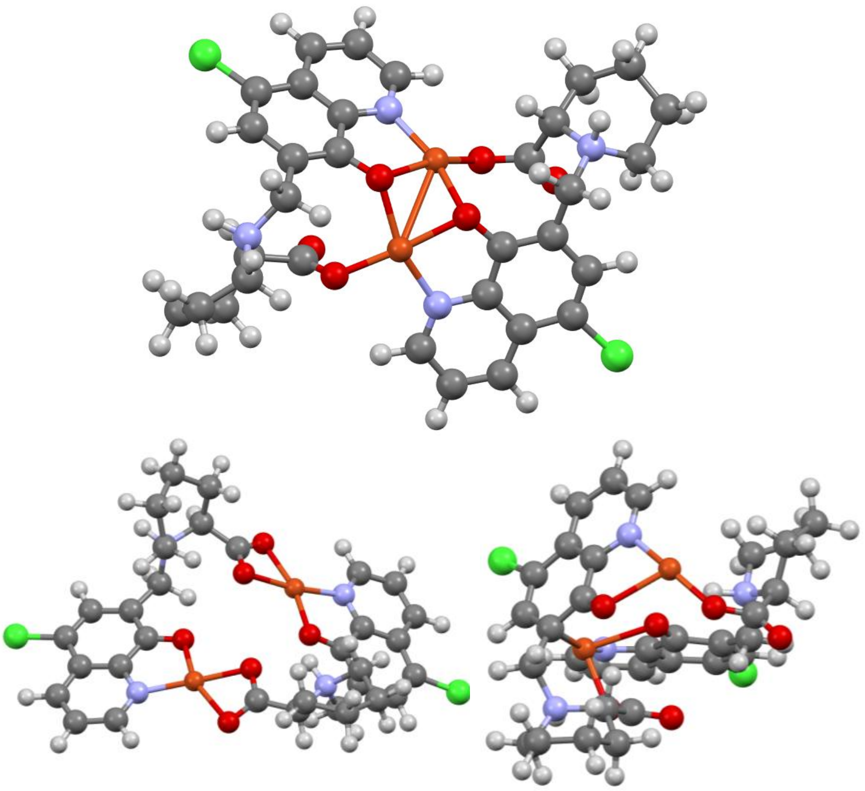

Figure S10. DFT calculated structures for the [Cu_2(HQCl-D-hProH)$_2$] dimers. The most stable structure is shown on the top. When only three donor atoms are found in the coordination sphere, the vacant positions are occupied by solvent molecules.

Table S8. Energy values and Cartesian coordinates for the [Cu_2(HQCl-L-Pro)$_2$] complex with 2x(N,O) donors of HQ moiety and 2x(N,O) donors of proline.

Sum of electronic and zero-point Energies (Eh)	-6030.556313
Sum of electronic and thermal Energies (Eh)	-6030.519161
Sum of electronic and enthalpy Energies (Eh)	-6030.518217
Sum of electronic and thermal Free Energies (Eh)	-6030.628156
Number of Imaginary Frequencies	0

Molecular Geometry in Cartesian Coordinates

Cu	1.399768	2.534325	0.066693
C	-0.211615	-4.980603	-0.545060
O	-0.018122	-6.112396	-0.962691
C	0.912138	-4.253148	0.198277
C	0.945077	-4.657051	1.693390
C	0.632365	-3.364778	2.455117
C	1.141571	-2.292372	1.514985
N	0.696927	-2.784196	0.192355
O	-1.331999	-4.350986	-0.635517
C	3.576261	0.809763	-0.156612
C	4.857512	0.232263	-0.166102
C	2.390944	0.065844	-0.450115

C	4.938347	-1.144933	-0.486480
C	2.524381	-1.286440	-0.739005
C	3.808128	-1.864225	-0.760599
C	4.390364	2.890059	0.435208
C	5.704900	2.400142	0.442378
N	3.370295	2.117572	0.144953
C	5.937972	1.085408	0.145694
O	1.256041	0.690220	-0.420276
Cl	6.494283	-1.928472	-0.533746
C	1.279982	-2.083460	-1.003140
Cu	-1.341783	-2.558663	0.023121
C	0.326951	5.014735	0.518497
O	0.181415	6.185516	0.837969
C	-0.878855	4.241109	-0.019267
C	-1.243600	4.665775	-1.445901
C	-1.911539	3.415205	-2.001745
C	-1.018193	2.316801	-1.454830
N	-0.620929	2.775343	-0.085760
O	1.445618	4.379840	0.564065
C	-3.526787	-0.831628	0.135382
C	-4.790578	-0.227863	0.026612
C	-2.361106	-0.108709	0.533092
C	-4.868413	1.155486	0.323970
C	-2.480164	1.255136	0.763577
C	-3.748835	1.859775	0.671253
C	-4.316244	-2.891241	-0.547964
C	-5.617855	-2.379421	-0.661005
N	-3.313098	-2.139425	-0.160778
C	-5.854517	-1.061686	-0.379664
O	-1.243374	-0.758041	0.627096
Cl	-6.406086	1.969732	0.231881
C	-1.225933	2.020650	1.069437
H	1.857918	-4.501519	-0.284179
H	0.238947	-5.455296	1.914713
H	1.940634	-5.016937	1.947835
H	-0.442972	-3.253882	2.615892
H	1.119291	-3.324024	3.427418
H	0.740185	-1.297586	1.689063
H	2.232875	-2.249730	1.549921
H	3.902057	-2.916346	-1.001098
H	4.168130	3.923185	0.669687
H	6.517812	3.069050	0.685261
H	6.946414	0.693929	0.150015
H	1.458748	-2.841243	-1.765360
H	0.529719	-1.393035	-1.385345
H	-1.724814	4.436426	0.640908
H	-1.879653	5.547639	-1.444876
H	-0.340838	4.894637	-2.017645
H	-2.926418	3.316461	-1.610151
H	-1.959023	3.401050	-3.089344
H	-1.481964	1.337400	-1.406101
H	-0.122325	2.222985	-2.068759
H	-3.840940	2.918915	0.879577
H	-4.090677	-3.924211	-0.779903
H	-6.417658	-3.033275	-0.977331

H	-6.851808	-0.652925	-0.472231
H	-0.490584	1.297021	1.414793
H	-1.388894	2.743811	1.867497

Table S9. Energy values and Cartesian coordinates for the [Cu_2(HQCl-L-Pro)$_2$] complex with 2x(N,O) donors of HQ moiety and bidentate carboxylate coordination.

Sum of electronic and zero-point Energies (Eh)	-6030.400587
Sum of electronic and thermal Energies (Eh)	-6030.368999
Sum of electronic and enthalpy Energies (Eh)	-6030.368055
Sum of electronic and thermal Free Energies (Eh)	-6030.464501
Number of Imaginary Frequencies	10

Molecular Geometry in Cartesian Coordinates

Cu	1.098262	-2.421122	0.507671
C	1.437987	2.647786	-0.480191
O	0.489783	3.361561	-0.031077
C	2.886869	3.081877	-0.345175
C	3.091074	4.564912	-0.656599
C	2.917860	5.284117	0.696014
C	2.917193	4.166103	1.747177
N	3.367167	2.979358	1.028962
O	1.140648	1.534331	-0.995817
C	3.664194	-1.620404	0.014988
C	4.982532	-1.522610	-0.467137
C	3.036532	-0.601720	0.798203
C	5.686850	-0.336866	-0.155027
C	3.755569	0.559836	1.024138
C	5.079389	0.659736	0.552480
C	3.348298	-3.689048	-0.985015
C	4.657246	-3.680794	-1.482548
N	2.882992	-2.695813	-0.262811
C	5.466123	-2.606754	-1.227495
O	1.821122	-0.820962	1.231194
Cl	7.329356	-0.136746	-0.702464
C	3.125851	1.730969	1.728750
Cu	-0.778885	1.904128	-0.522012
C	-1.033244	-3.355572	0.369440
O	-0.064766	-3.852592	-0.279186
C	-2.431556	-3.894988	0.184138
C	-2.888520	-4.708473	1.395929
C	-4.410991	-4.684002	1.251340
C	-4.710033	-3.357974	0.535927
N	-3.409752	-2.830723	0.118176
O	-0.788301	-2.411489	1.168710
C	-3.504405	1.681488	-0.376021
C	-4.877953	1.945120	-0.219710
C	-2.986769	0.396385	-0.726458
C	-5.761136	0.863083	-0.432187
C	-3.895701	-0.642076	-0.870526
C	-5.272726	-0.374903	-0.742989
C	-2.924518	3.864151	0.151294

C	-4.271503	4.211697	0.319425
N	-2.568039	2.646927	-0.186234
C	-5.238613	3.262058	0.134653
O	-1.692096	0.277110	-0.880457
Cl	-7.480558	1.107147	-0.291964
C	-3.410341	-2.047880	-1.107849
H	3.471761	2.430807	-0.994873
H	4.103209	4.696841	-1.037017
H	2.400511	4.924738	-1.417601
H	3.729698	5.990122	0.864500
H	1.983626	5.841355	0.731393
H	3.591596	4.362480	2.582983
H	1.908382	4.026652	2.160066
H	5.617094	1.579052	0.748137
H	2.673022	-4.512016	-1.180320
H	5.007883	-4.518851	-2.066961
H	6.476696	-2.580525	-1.612719
H	3.550543	1.838399	2.731435
H	2.053241	1.528624	1.863735
H	-2.419466	-4.529818	-0.714699
H	-2.471725	-5.714016	1.401678
H	-2.577678	-4.196460	2.307729
H	-4.738931	-5.522156	0.636087
H	-4.915582	-4.762818	2.212683
H	-5.361920	-3.528150	-0.331929
H	-5.218684	-2.640740	1.184357
H	-5.971495	-1.188114	-0.897399
H	-2.130744	4.586029	0.294038
H	-4.527398	5.224480	0.594466
H	-6.282969	3.513844	0.262240
H	-2.389093	-1.994069	-1.484903
H	-4.024121	-2.533137	-1.883320

Table S10. Energy values and Cartesian coordinates for the [Cu_2(HQCl-L-Pro)$_2$] complex with 2x(N,O) donors of HQ moiety and monodentate carboxylate coordination.

Sum of electronic and zero-point Energies (Eh)	-6030.473464
Sum of electronic and thermal Energies (Eh)	-6030.433495
Sum of electronic and enthalpy Energies (Eh)	-6030.432551
Sum of electronic and thermal Free Energies (Eh)	-6030.553562
Number of Imaginary Frequencies	0

Molecular Geometry in Cartesian Coordinates

Cu	1.325776	-2.749237	0.134524
C	1.387730	2.868249	0.396447
O	0.855226	2.994583	1.499970
C	2.884235	3.168997	0.203605
C	3.098146	4.673918	0.008821
C	3.224812	5.215515	1.441852
C	3.645620	4.004659	2.291545

N	3.744362	2.898797	1.341522
O	0.782410	2.527298	-0.680723
C	3.876406	-1.577293	-0.343596
C	5.142142	-1.402271	-0.916098
C	3.336924	-0.602627	0.630628
C	5.892385	-0.239099	-0.537071
C	4.159767	0.538219	0.953188
C	5.397828	0.686985	0.359011
C	3.540277	-3.531938	-1.513809
C	4.788358	-3.437368	-2.120452
N	3.095072	-2.627083	-0.650587
C	5.594366	-2.368174	-1.825176
O	2.219678	-0.776129	1.129479
Cl	7.445642	0.017531	-1.216846
C	3.661255	1.577086	1.918492
Cu	-1.101739	2.376126	-0.591361
C	-1.164740	-3.959625	0.306514
O	-0.800191	-4.870418	-0.438096
C	-2.606516	-3.962627	0.819895
C	-2.708615	-4.348034	2.303544
C	-3.934570	-3.566373	2.820811
C	-4.454888	-2.824048	1.592379
N	-3.269985	-2.665857	0.757175
O	-0.432405	-3.004497	0.734742
C	-3.863531	1.613093	-0.652909
C	-5.254728	1.768986	-0.694858
C	-3.242539	0.269584	-0.647118
C	-6.058280	0.583759	-0.737649
C	-4.130176	-0.873730	-0.667160
C	-5.492717	-0.682050	-0.727635
C	-3.539183	3.891178	-0.621397
C	-4.908786	4.133976	-0.658251
N	-3.029360	2.665630	-0.620729
C	-5.773719	3.071555	-0.697283
O	-2.014970	0.155980	-0.640374
Cl	-7.763931	0.720264	-0.797696
C	-3.549993	-2.256063	-0.608204
H	3.223314	2.603136	-0.666111
H	4.026346	4.824895	-0.543080
H	2.292213	5.143336	-0.554281
H	3.948269	6.027757	1.498256
H	2.268189	5.601768	1.790431
H	4.610536	4.147862	2.784972
H	2.897723	3.804497	3.066661
H	5.987640	1.561657	0.599295
H	2.880725	-4.360445	-1.731930
H	5.103353	-4.201822	-2.815382
H	6.566679	-2.267495	-2.286281
H	4.307523	1.553000	2.801489
H	2.649896	1.318787	2.249547
H	-3.157050	-4.697857	0.211934
H	-2.816849	-5.425450	2.417250
H	-1.805632	-4.043302	2.831291
H	-4.697395	-4.218113	3.243888
H	-3.637701	-2.858846	3.594305

H	-5.221395	-3.426712	1.074414
H	-4.899019	-1.857890	1.833058
H	-6.148642	-1.542571	-0.758966
H	-2.834409	4.710429	-0.591896
H	-5.270440	5.151669	-0.657442
H	-6.842243	3.230866	-0.729858
H	-2.613349	-2.267117	-1.167577
H	-4.246689	-2.955920	-1.098533

Table S11. Energy values and Cartesian coordinates for the [Cu_2(HQCl-D-Pro)$_2$] with 2x(N,O) donors of HQ moiety and 2x(N,O) donors of proline.

Sum of electronic and zero-point Energies (Eh)	-6030.556316
Sum of electronic and thermal Energies (Eh)	-6030.519163
Sum of electronic and enthalpy Energies (Eh)	-6030.518219
Sum of electronic and thermal Free Energies (Eh)	-6030.628166
Number of Imaginary Frequencies	0

Molecular Geometry in Cartesian Coordinates

Cu	-1.399664	2.534345	0.066413
C	0.212107	-4.980908	-0.544574
O	0.018945	-6.113055	-0.961395
C	-0.912058	-4.253239	0.197950
C	-0.945960	-4.656793	1.693095
C	-0.632737	-3.364606	2.454678
C	-1.141665	-2.292242	1.514350
N	-0.696971	-2.784234	0.191802
O	1.332283	-4.351019	-0.635518
C	-3.576266	0.809702	-0.156823
C	-4.857456	0.232121	-0.165805
C	-2.390941	0.065862	-0.450824
C	-4.938330	-1.145153	-0.486141
C	-2.524407	-1.286462	-0.739538
C	-3.808197	-1.864351	-0.760658
C	-4.390269	2.889976	0.435077
C	-5.704734	2.399979	0.442830
N	-3.370266	2.117538	0.144429
C	-5.937858	1.085195	0.146245
O	-1.256061	0.690291	-0.421489
Cl	-6.494230	-1.928778	-0.532752
C	-1.279968	-2.083456	-1.003697
Cu	1.341742	-2.558558	0.022642
C	-0.327250	5.014699	0.518890
O	-0.182034	6.185629	0.838006
C	0.878644	4.241396	-0.019113
C	1.243458	4.666311	-1.445623
C	1.911613	3.415888	-2.001611
C	1.018369	2.317266	-1.454939
N	0.620900	2.775583	-0.085843
O	-1.445786	4.379573	0.564531
C	3.526739	-0.831609	0.135296
C	4.790612	-0.227953	0.026873

C	2.361008	-0.108549	0.532646
C	4.868516	1.155412	0.324257
C	2.480178	1.255252	0.763444
C	3.748943	1.859786	0.671401
C	4.316241	-2.891409	-0.547342
C	5.617890	-2.379684	-0.660182
N	3.313010	-2.139452	-0.160607
C	5.854591	-1.061907	-0.378978
O	1.243153	-0.757713	0.626058
Cl	6.406269	1.969513	0.232417
C	1.225912	2.020768	1.069274
H	-1.857447	-4.501854	-0.285160
H	-0.240543	-5.455529	1.914918
H	-1.941917	-5.015846	1.947153
H	0.442669	-3.254000	2.615239
H	-1.119528	-3.323579	3.427031
H	-0.740160	-1.297490	1.688394
H	-2.232959	-2.249408	1.549226
H	-3.902092	-2.916564	-1.000759
H	-4.167984	3.923136	0.669362
H	-6.517585	3.068916	0.685826
H	-6.946277	0.693671	0.150875
H	-1.458623	-2.841263	-1.765904
H	-0.529663	-1.392996	-1.385803
H	1.724525	4.436662	0.641157
H	1.879386	5.548270	-1.444403
H	0.340718	4.895123	-2.017398
H	2.926477	3.317256	-1.609952
H	1.959195	3.401921	-3.089209
H	1.482252	1.337917	-1.406380
H	0.122625	2.223406	-2.069063
H	3.841057	2.918936	0.879673
H	4.090706	-3.924451	-0.778998
H	6.417736	-3.033659	-0.976149
H	6.851952	-0.653263	-0.471306
H	0.490581	1.297074	1.414553
H	1.388917	2.743835	1.867415

Table S12. Energy values and Cartesian coordinates for the [Cu_2(HQCl-D-Pro)$_2$] with 2x(N,O) donors of HQ moiety and monodentate carboxylate coordination.

Sum of electronic and zero-point Energies (Eh)	-6030.473464
Sum of electronic and thermal Energies (Eh)	-6030.433495
Sum of electronic and enthalpy Energies (Eh)	-6030.432551
Sum of electronic and thermal Free Energies (Eh)	-6030.553567
Number of Imaginary Frequencies	0

Molecular Geometry in Cartesian Coordinates

Cu	-1.326144	-2.749126	0.133920
C	-1.387338	2.868133	0.396617
O	-0.854680	2.994623	1.500047
C	-2.883839	3.168992	0.203937

C	-3.097687	4.673950	0.009361
C	-3.224129	5.215374	1.442473
C	-3.644940	4.004445	2.292063
N	-3.743864	2.898704	1.341913
O	-0.782143	2.527137	-0.680606
C	-3.876571	-1.577028	-0.343916
C	-5.142362	-1.401803	-0.916206
C	-3.336829	-0.602625	0.630412
C	-5.892462	-0.238663	-0.536806
C	-4.159529	0.538225	0.953330
C	-5.397683	0.687195	0.359396
C	-3.540690	-3.531458	-1.514552
C	-4.788837	-3.436666	-2.121041
N	-3.095346	-2.626804	-0.651197
C	-5.594751	-2.367488	-1.825441
O	-2.219435	-0.776256	1.128899
Cl	-7.445804	0.018238	-1.216281
C	-3.660750	1.576908	1.918693
Cu	1.102053	2.376287	-0.591521
C	1.164245	-3.959648	0.306726
O	0.799847	-4.870494	-0.437884
C	2.605934	-3.962592	0.820362
C	2.707765	-4.347718	2.304103
C	3.933666	-3.566024	2.821433
C	4.454206	-2.823902	1.592965
N	3.269465	-2.665865	0.757506
O	0.431853	-3.004419	0.734666
C	3.863761	1.612796	-0.653192
C	5.254983	1.768493	-0.695106
C	3.242593	0.269360	-0.647204
C	6.058375	0.583152	-0.737645
C	4.130078	-0.874081	-0.667018
C	5.492643	-0.682587	-0.727438
C	3.539736	3.890933	-0.622017
C	4.909373	4.133535	-0.658884
N	3.029737	2.665457	-0.621186
C	5.774158	3.070989	-0.697722
O	2.015012	0.155927	-0.640548
Cl	7.764047	0.719411	-0.797611
C	3.549706	-2.256330	-0.607903
H	-3.223041	2.603257	-0.665812
H	-4.025949	4.825048	-0.542403
H	-2.291798	5.143388	-0.553788
H	-3.947507	6.027674	1.499069
H	-2.267430	5.601499	1.790983
H	-4.609798	4.147660	2.785599
H	-2.896981	3.804132	3.067080
H	-5.987387	1.561865	0.599949
H	-2.881229	-4.359977	-1.732900
H	-5.103954	-4.200955	-2.816097
H	-6.567111	-2.266651	-2.286410
H	-4.306842	1.552745	2.801815
H	-2.649345	1.318488	2.249513
H	3.156549	-4.697965	0.212645
H	2.815961	-5.425116	2.418023

H	1.804697	-4.042863	2.831635
H	4.696395	-4.217736	3.244722
H	3.636705	-2.858363	3.594771
H	5.220815	-3.426661	1.075258
H	4.898288	-1.857700	1.833554
H	6.148467	-1.543191	-0.758596
H	2.835079	4.710289	-0.592630
H	5.271165	5.151179	-0.658216
H	6.842706	3.230145	-0.730279
H	2.613129	-2.267352	-1.167388
H	4.246377	-2.956350	-1.098036

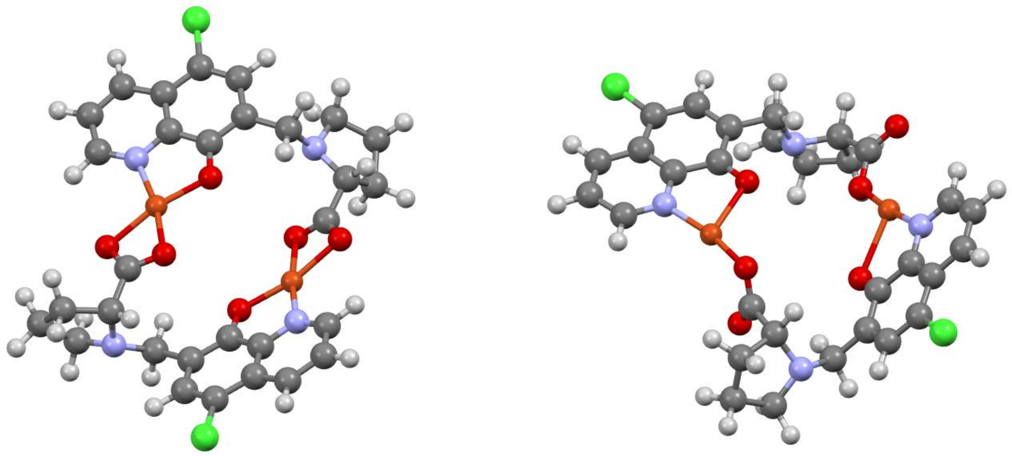

Figure S11. DFT calculated structures for the [Cu_2(HQCl-D-Pro)$_2$] dimers. The most stable structure is shown in Figure 8. When only three donor atoms are found in the coordination sphere, the vacant positions are occupied by solvent molecules.

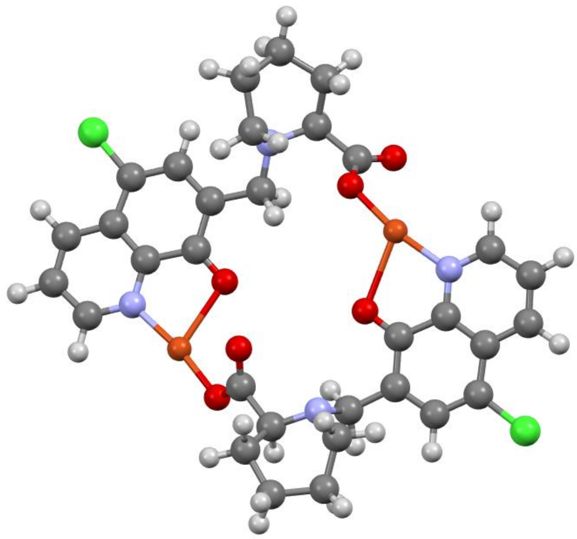

Figure S12. DFT calculated structures for less stable [Cu_2(HQCl-D-Pro)$_2$] dimer. The most stable structure is shown in Figure 9. When only three donor atoms are found in the coordination sphere, the vacant positions are occupied by solvent molecules.

Table S13. Energy values and Cartesian coordinates for the [Cu_2(HQCl-D-hPro)$_2$] with 2x(N,O) donors of HQ moiety and 2x(N,O) donors of proline.

Sum of electronic and zero-point Energies (Eh)	-6109.13816
Sum of electronic and thermal Energies (Eh)	-6109.099248
Sum of electronic and enthalpy Energies (Eh)	-6109.098304
Sum of electronic and thermal Free Energies (Eh)	-6109.208492
Number of Imaginary Frequencies	0

Molecular Geometry in Cartesian Coordinates

Cu	1.557643	-0.991040	0.955712
C	-2.666650	-3.458652	-0.426978
O	-1.640013	-2.907512	-0.959012
C	-3.855109	-2.535185	-0.165166
C	-4.823984	-2.639293	-1.341674
C	-6.127255	-1.891253	-1.049013
C	-4.430493	-0.159456	-0.427427
N	-3.398450	-1.126717	0.043689
O	-2.772928	-4.653633	-0.187007
C	-0.035229	1.239752	1.672911
C	-0.559438	2.530954	1.871307
C	-0.868901	0.082610	1.656812
C	-1.955488	2.645215	2.071592
C	-2.243011	0.259010	1.765275
C	-2.759673	1.542394	2.013078
C	2.098516	2.042356	1.374595
C	1.669756	3.359599	1.586131
N	1.282327	1.013244	1.407995
C	0.350056	3.607674	1.826030
O	-0.318776	-1.075454	1.452911
Cl	-2.660946	4.210703	2.353567
C	-3.125535	-0.923796	1.507987
Cu	-1.557764	-0.990531	-0.955971
C	2.666176	-3.459190	0.426746
O	2.772485	-4.654183	0.186828
C	3.854678	-2.535697	0.165176
C	4.823396	-2.639887	1.341810
C	5.848506	-0.650038	0.193396
C	4.429926	-0.159927	0.427756
N	3.398047	-1.127203	-0.043686
O	1.639538	-2.908008	0.958713
C	0.035483	1.239902	-1.673023
C	0.559957	2.531023	-1.871288
C	0.868942	0.082627	-1.657049
C	1.956036	2.645048	-2.071508
C	2.243096	0.258771	-1.765377
C	2.760017	1.542079	-2.013027

C	-2.098075	2.042879	-1.374603
C	-1.669073	3.360062	-1.586062
N	-1.282099	1.013604	-1.408093
C	-0.349330	3.607909	-1.825932
O	0.318526	-1.075304	-1.453286
Cl	2.661805	4.210435	-2.353286
C	3.125392	-0.924185	-1.508021
H	-4.365048	-2.883089	0.736786
H	-5.015847	-3.694271	-1.531843
H	-4.337422	-2.241297	-2.236115
H	-6.824401	-2.550226	-0.529963
H	-6.597761	-1.604940	-1.990434
H	-4.262206	0.780330	0.090317
H	-4.280228	0.017305	-1.493055
H	-3.827581	1.662431	2.148031
H	3.138036	1.847865	1.169225
H	2.393436	4.160245	1.540520
H	-0.004321	4.618586	1.973587
H	-2.628289	-1.824363	1.863981
H	-4.078741	-0.832628	2.033837
H	4.364757	-2.883549	-0.736718
H	5.015301	-3.694878	1.531866
H	4.336688	-2.242054	2.236240
H	5.987912	-0.873112	-0.866629
H	6.541193	0.157233	0.430232
H	4.261683	0.779937	-0.089871
H	4.279430	0.016674	1.493380
H	3.827958	1.661928	-2.147876
H	-3.137640	1.848599	-1.169272
H	-2.392599	4.160845	-1.540413
H	0.005240	4.618765	-1.973412
H	2.628043	-1.824652	-1.864115
H	4.078679	-0.833147	-2.033745
C	-5.848992	-0.649686	-0.192831
H	-6.541777	0.157578	-0.429408
H	-5.988150	-0.872917	0.867193
C	6.126669	-1.891722	1.049454
H	6.823931	-2.550590	0.530429
H	6.597013	-1.605521	1.990990

Table S14. Energy values and Cartesian coordinates for the [Cu_2(HQCl-D-hPro)$_2$] with 2x(N,O) donors of HQ moiety and monodentate carboxylate coordination.

Sum of electronic and zero-point Energies (Eh)	-6109.039621
Sum of electronic and thermal Energies (Eh)	-6108.997739
Sum of electronic and enthalpy Energies (Eh)	-6108.996794
Sum of electronic and thermal Free Energies (Eh)	-6109.121207
Number of Imaginary Frequencies	0

Molecular Geometry in Cartesian Coordinates

Cu	-1.973817	-3.326283	-0.582971
C	-0.709954	3.981186	-0.466021
O	-0.122272	4.631965	-1.330876
C	-2.235965	4.140064	-0.336252
C	-2.518051	5.354310	0.570093
C	-3.916686	5.265843	1.163105
C	-3.092952	2.917944	1.596998
N	-2.968618	2.975502	0.146231
O	-0.156420	3.188076	0.373802
C	-4.303097	-1.712769	-0.552361
C	-5.649939	-1.363446	-0.396290
C	-3.261124	-0.674380	-0.668969
C	-5.968779	0.031836	-0.291277
C	-3.659405	0.698804	-0.472208
C	-4.992056	1.011620	-0.307709
C	-4.800493	-3.958836	-0.532530
C	-6.162380	-3.699209	-0.409671
N	-3.893193	-2.992841	-0.600433
C	-6.592427	-2.399212	-0.332804
O	-2.094352	-0.985645	-0.927495
Cl	-7.607146	0.506174	-0.108334
C	-2.576451	1.735441	-0.471643
Cu	1.679167	2.802434	0.216510
C	0.531835	-3.341920	0.439488
O	-0.177026	-3.910730	-0.467793
C	1.983113	-3.838374	0.498138
C	2.109039	-4.865100	1.623157
C	4.578726	-4.556552	1.169252
C	4.120863	-3.131542	1.455937
N	2.942245	-2.744135	0.672764
O	0.116019	-2.517911	1.250595
C	4.235389	1.565216	-0.189313
C	5.618673	1.499902	-0.402046
C	3.389227	0.349466	-0.258297
C	6.186202	0.224202	-0.719699
C	4.050979	-0.903540	-0.560933
C	5.407551	-0.919661	-0.800789
C	4.334479	3.838588	0.166971
C	5.713555	3.856612	-0.013387
N	3.614364	2.726438	0.078713
C	6.361509	2.685101	-0.305321
O	2.172953	0.430116	-0.080025
Cl	7.868373	0.094000	-1.010976
C	3.251897	-2.171503	-0.632627
H	-2.602121	4.364022	-1.342369
H	-2.383516	6.266292	-0.011035
H	-1.786856	5.384223	1.383472
H	-4.644262	5.191850	0.351500
H	-4.151673	6.175639	1.717710
H	-3.505635	1.946775	1.873022
H	-2.109081	2.987127	2.078380
H	-5.268106	2.050723	-0.183771
H	-4.432419	-4.974621	-0.571601
H	-6.859671	-4.522822	-0.364268
H	-7.643255	-2.172396	-0.220168

H	-1.681065	1.295980	-0.015540
H	-2.303818	1.929172	-1.515926
H	2.176853	-4.364713	-0.448522
H	1.313080	-5.603965	1.520576
H	1.947238	-4.356784	2.576295
H	4.787867	-4.653877	0.100267
H	5.515468	-4.762488	1.689266
H	4.920530	-2.418970	1.261434
H	3.884263	-3.032081	2.518735
H	5.889987	-1.855850	-1.050950
H	3.795316	4.749715	0.385710
H	6.250804	4.789644	0.071810
H	7.430926	2.670150	-0.461012
H	2.302277	-1.949237	-1.124084
H	3.790972	-2.884643	-1.276685
C	-4.023093	4.037180	2.077886
H	-3.755771	4.304987	3.102620
H	-5.055413	3.681963	2.099362
C	3.481499	-5.547522	1.592730
H	3.455944	-6.392362	0.901650
H	3.700243	-5.954987	2.581521

Table S15. Crystal data and structure refinement for HQCl-L-Pro×MeOH (I) and [Cu(HQCl-L-ProH$_{-1}$)$_2$]×3H_2O (II)

	I	II
CCDC	2442499	2442500
Color/Shape	Colorless/platelet	Blue/needle
Empirical formula	$C_{16}H_{19}ClN_2O_4$	$C_{30}H_{33}Cl_2CuN_4O_9$
Moiety formula	$C_{15}H_{15}ClN_2O_3$, CH_3OH	$C_{30}H_{28}Cl_2CuN_4O_6$, 3(H_2O)
Formula weight	338.78	728.069
Temperature (K)	143(2)	295(2)
Radiation and wavelength λ (Å)	Mo-Kα, (0.71075)	Cu-Kα, (1.54187)
Crystal system	orthorhombic	triclinic
Space group	$P2_12_12$	$P1$
Unit cell dimensions		
a (Å)	15.1993(11)	7.1968(6)
b (Å)	9.7752(7)	7.8181(6)
c (Å)	10.4541(8)	14.0457(11)
α (°)	90	82.400(6)
β (°)	90	81.792(6)
γ (°)	90	85.773(6)
Volume (Å^3)	1553.2(2)	774.11(11)
Z/Z'	4/1	1/1
Density (calculated) (Mg/m^3)	1.449	1.562
Absorption coefficient, μ (mm^{-1})	0.269	3.113
F(000)	712	376
Crystal size (mm)	0.50 x 0.30 x 0.10	0.60 x 0.1 x 0.1
Absorption correction	numerical	numerical
Max. and min. transmission	0.606, 0.816	0.627, 0.926
θ–range for data collection (°)	$3.152 \leq \theta \leq 27.462$	$3.203 \leq \theta \leq 68.202$
Index ranges	$-19 \leq h \leq 19$;$-12 \leq k \leq 12$;$-13 \leq l \leq 13$	$-8 \leq h \leq 8$;$-9 \leq k \leq 9$;$-16 \leq l \leq 16$
Reflections collected	35436	7379
Completeness to 2θ	0.997	0.978
Independent reflections, (R_{int})	3549 (0.0524)	4662 (0.0402)
Reflections $I>2\sigma(I)$	3353	3816
Refinement method	full-matrix least-squares on F^2	full-matrix least-squares on F^2
Data / restraints / parameters	3549 /0 /218	4662 /10 /415
Flack parameter	0.07(7)	0.06(3)
Goodness-of-fit on F^2	1.089	1.046
Final *R* indices [$I>2\sigma(I)$], R_1, wR_2	0.0363, 0.0763	0.0572, 0.1218
R indices (all data), R_1, wR_2	0.0402, 0.0776	0.0704, 0.1312
Max. and mean shift/esd	0.000; 0.000	0.002; 0.000
Largest diff. peak and hole (e.Å^{-3})	0.265; -0.188	0.482; -0.290

Table S16. Comparison of selected bond angles in crystal (I) and (II)

I		II			
		ligand 1		ligand 2	
**Bond**	**angle (°)**	**bond**	**angle (°)**	**bond**	**angle (°)**
C7-C11-N2	112.3(2)	C7-C11-N2	114(1)	C27-C31-N4	112(1)
C11-N2-C15	114.3(2)	C11-N2-C15	116.1(8)	C31-N4-C35	115(1)
C11-N2-C12	111.1(2)	C11-N2-C12	116.0(8)	C31-N4-C32	113(1)
N2-C15-C16	110.1(2)	N2-C15-C16	111(1)	N4-C35-C36	108(1)
O3-C16-C15	113.9(2)	O3-C16-C15	117(1)	O6-C36-C35	116(1)
O2-C16-C15	118.6(2)	O2-C16-C15	118(1)	O5-C36-C35	120(1)
O2-C16-O3	127.5(2)	O2-C16-O3	126(1)	O5-C36-O6	124(1)
N2-C15-C14	103.5(2)	N2-C15-C14	105(1)	N4-C35-C34	105(1)
C15-C14-C13	102.7(2)	C15-C14-C13	109(1)	C35-C34-C33	103(1)
C12-C13-C14	103.4(2)	C12-C13-C14	108(1)	C32-C33-C34	104(1)
C15-N2-C12	107.4(2)	C15-N2-C12	106(1)	C35-N4-C32	107(1)
C14-C15-C16	111.3(2)	C14-C15-C16	118(1)	C34-C35-C36	112(1)
C13-C12-N2	105.1(2)	C13-C12-N2	104(1)	C33-C32-N4	103(1)
C7-C11-N2-C12	177.8(2)	C7-C11-N2-	64(1)	C27-C31-N4-	-56(1)
C7-C11-N2-C15	56.1(2)	C7-C11-N2-	-62(1)	C27-C31-N4-	-179(1)
N2-C15-C16-	-9.6(1)	N2-C15-C16-	-8(1)	N2-C15-C16-	28(1)

Table S17. Hydrogen-bond geometry of (I)

**D-H…A**	**D-H** (Å)	**H…A**(Å)	**D…A**(Å)	**D-H…A** (°)	**symmetry codes**
O1-H1O...N1	0.82	2.36	2.756(3)	111	Intra
O1-H1O...O3	0.82	1.92	2.638(2)	146	1-x,1-y,z
N2-H2N...O2	0.80(3)	2.31(3)	2.672(2)	108(2)	Intra
N2-H2N...O3	0.80(3)	1.99(3)	2.741(2)	156(3)	1/2-x,1/2+y,2-z
O4-H4O...O2	0.82	2.38	2.989(3)	131	1-x,1-y,z
O4-H4O...O1	0.82	2.30	3.004(3)	144	3/2-x,-1/2+y,2-z
C4-H4...Cl1	0.93	2.72	3.092(3)	105	Intra
C6-H6...O2	0.93	2.55	3.390(3)	150	1/2-x,1/2+y,2-z
C11-H11B...O1	0.97	2.41	2.785(3)	102	Intra
C15-H15...O1	0.98	2.35	3.172(3)	141	Intra

Table S18. Selected bond lengths (Å) and angles (°) of the coordination sphere of [Cu(HQCl-L-ProH$_{-1}$)$_2$] complex in crystal (II)

Bond	Distance (Å)	Bond	Angles (°)
Cu-O1	1.923(7)	O4-Cu-O1	177.6(4)
Cu-N1	1.96(1)	O1-Cu-N1	84.7(3)
Cu-O4	1.902(7)	O4-Cu-N1	96.1(3)
Cu-N3	1.975(9)	O4-Cu-N3	85.1(4)
		O1-Cu-N3	94.1(3)
		N1-Cu-N3	177.8(5)

Table S19. Hydrogen-bond geometry of (II)

**D-H…A**	**D-H** (Å)	**H…A**(Å)	**D…A**(Å)	**D-H…A** (°)	**symmetry codes**
O2W-H2WA...O2	165.9(9)	0.843(10)	1.977(11)	2.802(11)	x,y,1+z
O2W-H2WA...O3	137.1(8)'	0.843(10)	2.574(11)	3.243(11)	x,y,1+z
N2-H2...O2	101.2(5)	1.031(8)	2.315(9)	2.710(10)	Intra
N2-H2...O6	149.4(6)'	1.031(8)	1.794(8)	2.732(8)	x,y,-1+z
O2W-H2WB...O6	150.7(8)	0.840(10)	2.268(10)	3.029(10)	1+x,y,z
O1W-H1WA...O5	149.5(7)	0.841(10)	2.376(9)	3.130(10)	1+x,y,z
O1W-H1WA...O6	152.4(7)'	0.841(10)	2.148(9)	2.919(10)	1+x,y,z
O3W-H3W...O2W	157.7(9)	0.843(9)	2.013(10)	2.811(10)	x,-1+y,z
N4-H4...O4	116.1(7)	0.773(8)	2.456(8)	2.881(8)	Intra
N4-H4...O5	129.9(7)'	0.773(8)	2.047(9)	2.610(9)	Intra
O1W-H1WB...O3W	130.0(8)	0.841(11)	2.168(11)	2.784(11)	
C2-H2A...O5	136.4(10)	0.929(13)	2.303(11)	3.044(11)	Intra
C3-H3...O3W	150.9(10)	0.930(14)	2.558(12)	3.401(11)	-1+x,y,z
C4-H4A...Cl1	103.1(9)	0.930(13)	2.785(10)	3.129(9)	Intra
C6-H6...O3	150.1(9)	0.930(12)	2.480(10)	3.319(10)	-1+x,y,z
C11-H11B...O3	141.4(8)	0.970(11)	2.454(10)	3.269(10)	-1+x,y,z
C14-H14A...O3	105.4(12)	0.97(2)	2.446(15)	2.860(15)	Intra
C15-H15...O1	143.1(8)	0.981(10)	2.408(9)	3.245(9)	Intra
C24-H24...Cl2	104.1(8)	0.931(12)	2.777(9)	3.136(9)	Intra
C32-H32A...O3W	139.2(8)	0.970(13)	2.592(11)	3.386(11)	x,1+y,z
C35-H35...O2	127.4(7)	0.980(10)	2.520(11)	3.210(11)	x,y,1+z

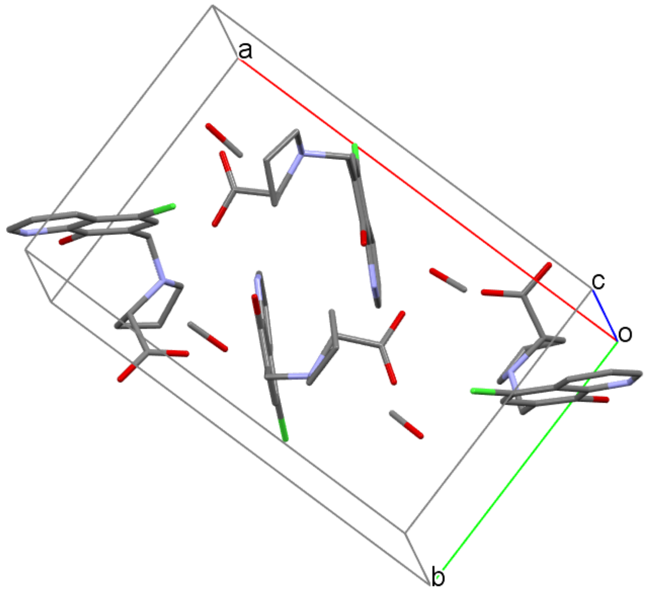

Figure S13. Unit cell containing four molecules in the crystal (I).

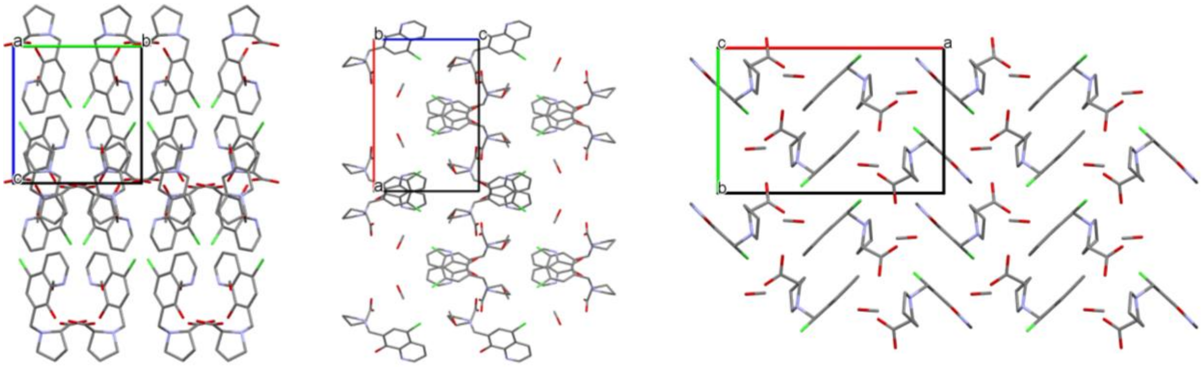

Figure S14. Packing arrangements in crystal (I) viewed from the crystallographic directions ’a’, ’b’ and ’c’.

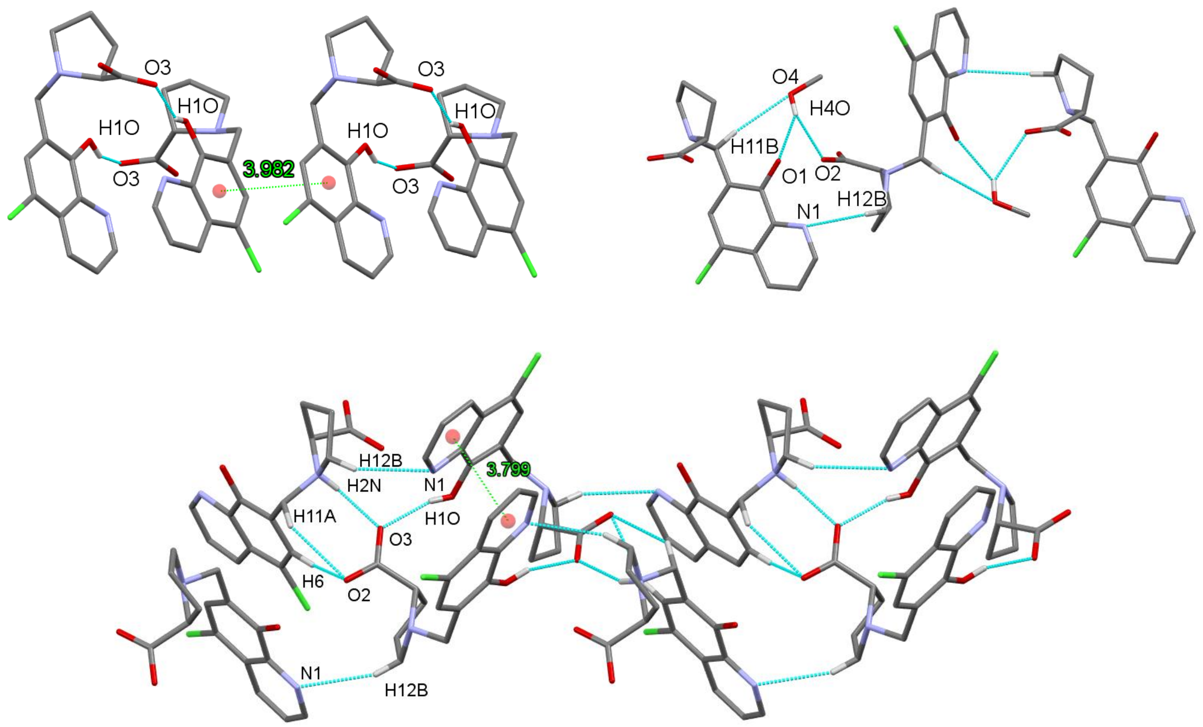

Figure S15. Packing arrangements from different view points in crystal (I) showing the main hydrogen bond connections and the stacking interactions between aromatic rings (values are collected in Table S17).

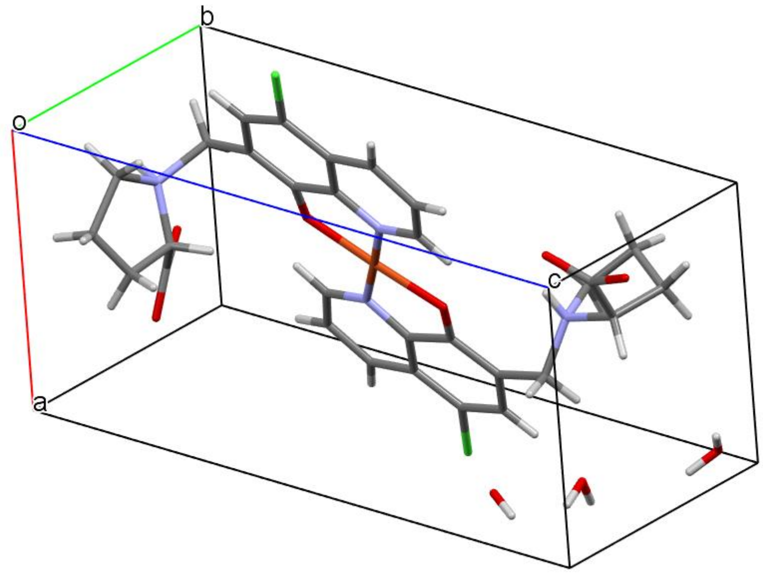

Figure S16. Unit cell in crystal (II).

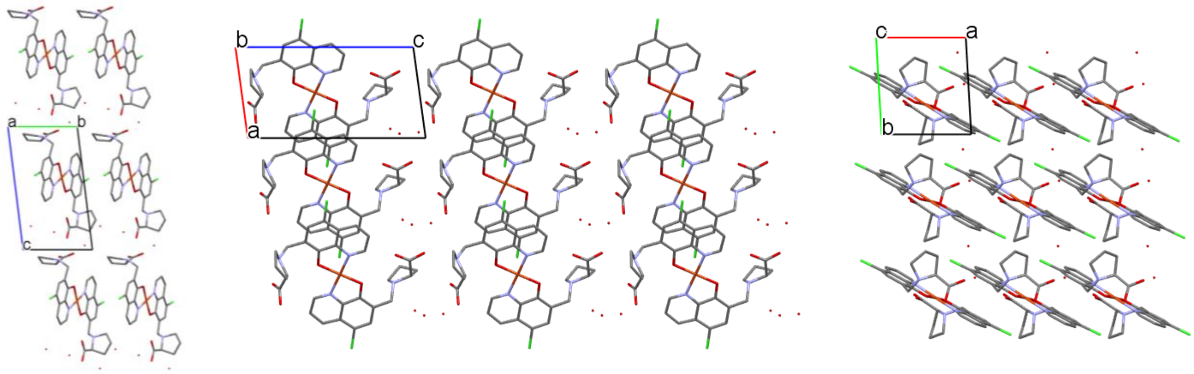

Figure S17. Packing arrangements in crystal (II) viewed from the crystallographic directions ’a’, ’b’ and ’c’.

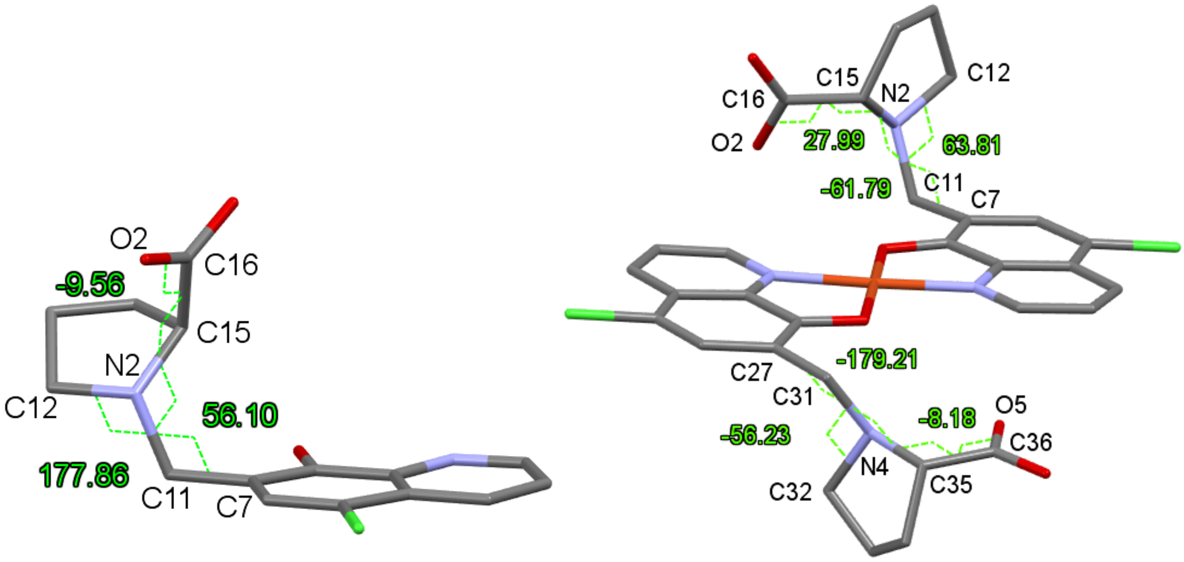

Figure S18. Comparison of the conformaiton of ligand HQCl-L-Pro in its crystals (I) and (II) with selected torsion angles. More structural data are collected in Table S6 and S7.

a)

b)

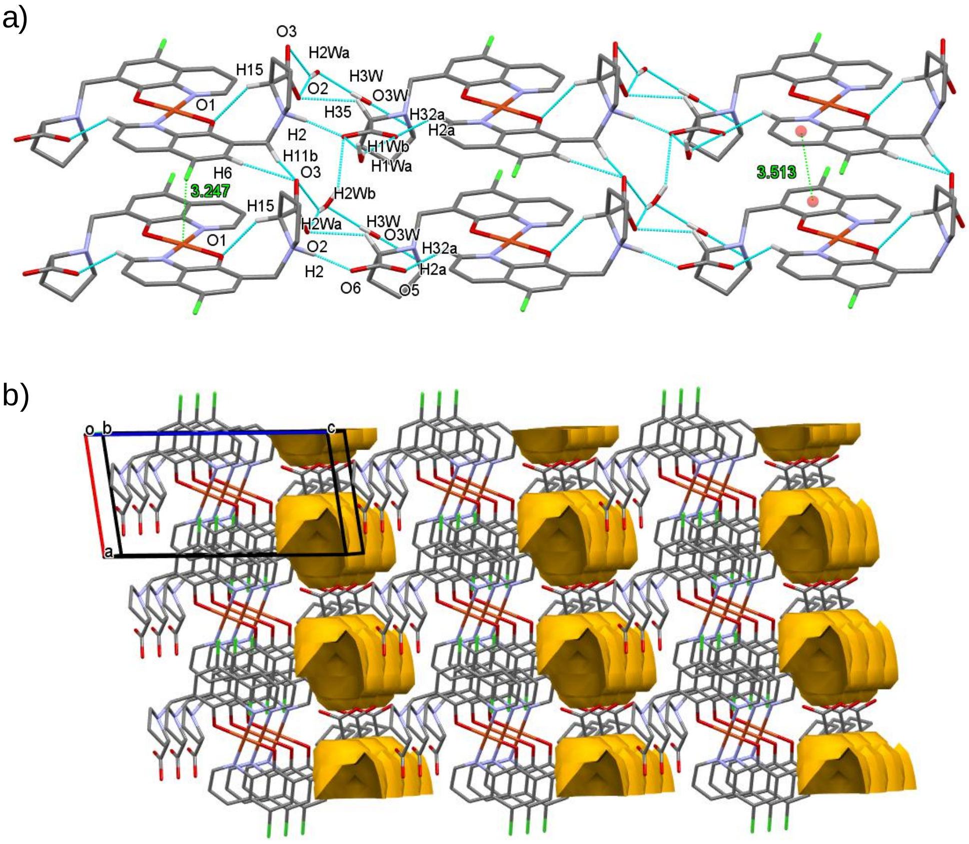

Figure S19. Crystal packing of (II) showing (a) the main hydrogen bond connections and (b) channels in the crystal filled with water of crystallization. The voids volume is 65.0 Å^3 which is 8.4% of the unit cell volume, calculated by Mercury software.

Equations for stability constant determination

Calculation of the overall stability (formation) constants (β) are based on the following equation (1): (where M: metal ion, L: fully deprotonated ligand, H: proton; p, q, r: stoichiometric coefficients):

$$\mathrm{pM} + \mathrm{qL} + \mathrm{rH} \rightleftharpoons M_pL_qH_r \qquad (1)$$

Definition of the overall stability (formation) constants (β) forming associated (metal complexes or protonated forms of the ligand) (2):

$$\beta_{pqr} = \frac{[M_pL_qH_r]}{[M]^p[L]^q[H]^r} \qquad (2)$$

The mass balance equations that can be written for components M, L, and H (3-5):

$$c_M = [M] + \sum_{i=1}^{n} p_i\beta_{pqr}[M]^p[L]^q[H]^r \qquad (3)$$

$$c_L = [L] + \sum_{i=1}^{n} q_i\beta_{pqr}[M]^p[L]^q[H]^r \qquad (4)$$

$$c_H = [H] + \sum_{i=1}^{n} r_i \beta_{pqr[M]^p} [L]^q [H]^r \quad (5)$$

References

(SI1) Pivarcsik, T.; Tóth, S.; Pósa, S. P.; May, N. V.; Kováts, É.; Spengler, G.; Kántor, I.; Rolya, A.; Feczkó, T.; Szatmári, I.; Szakács, G.; Enyedy, É. A. Organometallic Half-Sandwich Complexes of 8-Hydroxyquinoline-Derived Mannich Bases with Enhanced Solubility: Targeting Multidrug Resistant Cancer. *Inorg. Chem.* **2024**, 63 (50), 23983–23998. DOI: 10.1021/acs.inorgchem.4c04398

(SI2) Dömötör, O.; Pivarcsik, T.; Bakos É.; Özvegy-Laczka, Cs.; Nezafat Yazdi, Z, Hetényi, A.; Martinek, T.; Szatmári I.; Tóth, S.; Szakács, G.; Borics, A.; Enyedy, É. A. Comparative study of multidrug resistance-targeting 8-hydroxyquinoline-amino acid conjugates: anticancer effect, interaction with human serum albumin and organic anion transporting polypeptides. *Eur. J. Pharm. Sci.* **2025**, 212, (1), 107187. DOI: 10.1016/j.ejps.2025.107187

(SI3) Mészáros, J. P.; Poljarević, J. M.; Szatmári, I.; Csuvik, O.; Fülöp, F.; Szoboszlai, N.; Spengler, G.; Enyedy, É. A. An 8-hydroxyquinoline–proline hybrid with multidrug resistance reversal activity and the solution chemistry of its half-sandwich organometallic Ru and Rh complexes. *Dalton Trans*. **2020**, 49 (23), 7977–7992. DOI: 10.1039/D0DT01256D

(SI4) Pivarcsik, T.; Dömötör, O.; Mészáros, J. P.; May, N. V.; Spengler, G.; Csuvik, O.; Szatmári, I.; Enyedy, É. A. 8-Hydroxyquinoline-Amino Acid Hybrids and Their Half-Sandwich Rh and Ru Complexes: Synthesis, Anticancer Activities, Solution Chemistry and Interaction with Biomolecules. *Int. J. Mol. Sci.* **2021**, 22 (20), 11281. DOI: 10.3390/ijms222011281
